# Antiprotozoal Activity of Plants Used in the Management of Sleeping Sickness in Angola and Bioactivity-Guided Fractionation of *Brasenia schreberi* J.F.Gmel and *Nymphaea lotus* L. Active against *T. b. rhodesiense*

**DOI:** 10.3390/molecules29071611

**Published:** 2024-04-03

**Authors:** Nina Vahekeni, Théo Brillatz, Marjan Rahmaty, Monica Cal, Sonja Keller-Maerki, Romina Rocchetti, Marcel Kaiser, Sibylle Sax, Kevin Mattli, Evelyn Wolfram, Laurence Marcourt, Emerson Ferreira Queiroz, Jean-Luc Wolfender, Pascal Mäser

**Affiliations:** 1 Department of Medical Parasitology and Infection Biology, Swiss Tropical and Public Health Institute, 4123 Allschwil, Switzerland; monica.cal@swisstph.ch (M.C.); sonja.maerki@swisstph.ch (S.K.-M.); romina.rocchetti@swisstph.ch (R.R.); marcel.kaiser@swisstph.ch (M.K.); sibylle.sax@swisstph.ch (S.S.); pascal.maeser@swisstph.ch (P.M.); 2 Faculty of Science, University of Basel, 4002 Basel, Switzerland; 3 School of Pharmaceutical Sciences, University of Geneva, CMU, 1211 Geneva, Switzerland; theo.brillatz@unige.ch (T.B.); laurence.marcourt@unige.ch (L.M.); emerson.ferreira@unige.ch (E.F.Q.); jean-luc.wolfender@unige.ch (J.-L.W.); 4 Institute of Pharmaceutical Sciences of Western Switzerland, University of Geneva, CMU, 1211 Geneva, Switzerland; 5 Phytopharmacy & Natural Products, Institute of Chemistry and Biotechnology, Zürich University of Applied Sciences (ZHAW), 8820 Wädenswil, Switzerlandevelyn.wolfram@zhaw.ch (E.W.)

**Keywords:** ethnopharmacology, African medicinal plant, antiprotozoal, trypanosomiasis, *Brasenia schreberi*, *Nymphaea lotus*, Angola

## Abstract

Folk medicine is widely used in Angola, even for human African trypanosomiasis (sleeping sickness) in spite of the fact that the reference treatment is available for free. Aiming to validate herbal remedies in use, we selected nine medicinal plants and assessed their antitrypanosomal activity. A total of 122 extracts were prepared using different plant parts and solvents. A total of 15 extracts from seven different plants exhibited in vitro activity (>70% at 20 µg/mL) against *Trypanosoma brucei rhodesiense* bloodstream forms. The dichloromethane extract of *Nymphaea lotus* (leaves and leaflets) and the ethanolic extract of *Brasenia schreberi* (leaves) had IC_50_ values ≤ 10 µg/mL. These two aquatic plants are of particular interest. They are being co-applied in the form of a decoction of leaves because they are considered by local healers as male and female of the same species, the ethnotaxon “longa dia simbi”. Bioassay-guided fractionation led to the identification of eight active molecules: gallic acid (IC_50_ 0.5 µg/mL), methyl gallate (IC_50_ 1.1 µg/mL), 2,3,4,6-tetragalloyl-glucopyranoside, ethyl gallate (IC_50_ 0.5 µg/mL), 1,2,3,4,6-pentagalloyl-β-glucopyranoside (IC_50_ 20 µg/mL), gossypetin-7-*O*-β-glucopyranoside (IC_50_ 5.5 µg/mL), and hypolaetin-7-*O*-glucoside (IC_50_ 5.7 µg/mL) in *B. schreberi*, and 5-[(8Z,11Z,14Z)-heptadeca-8,11,14-trienyl] resorcinol (IC_50_ 5.3 µg/mL) not described to date in *N. lotus*. Five of these active constituents were detected in the traditional preparation. This work provides the first evidence for the ethnomedicinal use of these plants in the management of sleeping sickness in Angola.

## 1. Introduction

The extensive use of folk medicine in Africa, composed mainly of medicinal plants, is linked to cultural as well as economic reasons. This is why the World Health Organisation (WHO) encourages African member states to promote and integrate traditional medical practices in their health systems [1]. In Angola, 72% (Percentage given by the hosting country’s speaker at the 1st National Conference of Traditional Medicine and Complementary Practices held in Luanda in August 2012) of the population uses herbal medicines to treat various medical affections, including parasitic infections such as human African trypanosomiasis (HAT), also called sleeping sickness.

HAT is a vector-borne Neglected Tropical Disease (NTD) that is transmitted by the bite of infected tsetse flies (*Glossina* spp.). HAT is caused by two subspecies of the protozoan parasite *Trypanosoma brucei*: *T. b. gambiense* in west and central Africa including Angola is responsible for the chronic form, whereas *T. b. rhodesiense*, prevalent in eastern Africa, causes the acute form [2]. Both forms are fatal if untreated. The majority of HAT-cases are of the gambiense form (g-HAT) and 57 million people are at risk of contracting g-HAT [3]. In Angola, g-HAT is endemic in the northwestern part. It is prevalent in seven of eighteen provinces [4]. It affects mainly remote rural communities, where the health infrastructure is basic and accessibility complicated [4].

Until recently, the chemotherapy of HAT relied on only five drugs, according to disease stage and parasite subspecies. This was unsatisfactory because the clinically available drugs had limitations such as toxicity, resistance, high cost, and parenteral administration [5]. The recent approval of fexinidazole as a new oral drug for both stages of g-HAT greatly facilitates the treatment and will increase the coverage [6,7]. The current reference treatment is available for free in Angola. Nevertheless, a previous ethnobotanical study reporting the use of local herbal remedies against sleeping sickness pointed out that 40% of the infected patients had recoursed first to herbal remedies before receiving the medical reference treatment [8]. Therefore, the investigation of herbal remedies is of high practical relevance. There have been several reports on the antitrypanosomal activity of traditionally used African medicinal plants [9,10,11,12,13,14,15,16,17,18,19]; this is the first such study from Angola.

The laboratory results demonstrate that the medicinal plants in use to treat HAT possess antitrypanosomal activity. Bioassay-guided fractionation led to the identification of eight active molecules. Furthermore, the study provides evidence for the antitrypanosomal potential of a local preparation made of *B. schreberi* and *N. lotus* in the management of sleeping sickness in Angola.

## 2. Results and Discussion

### 2.1. Selection of the Candidate Plants

**Figure 1:**
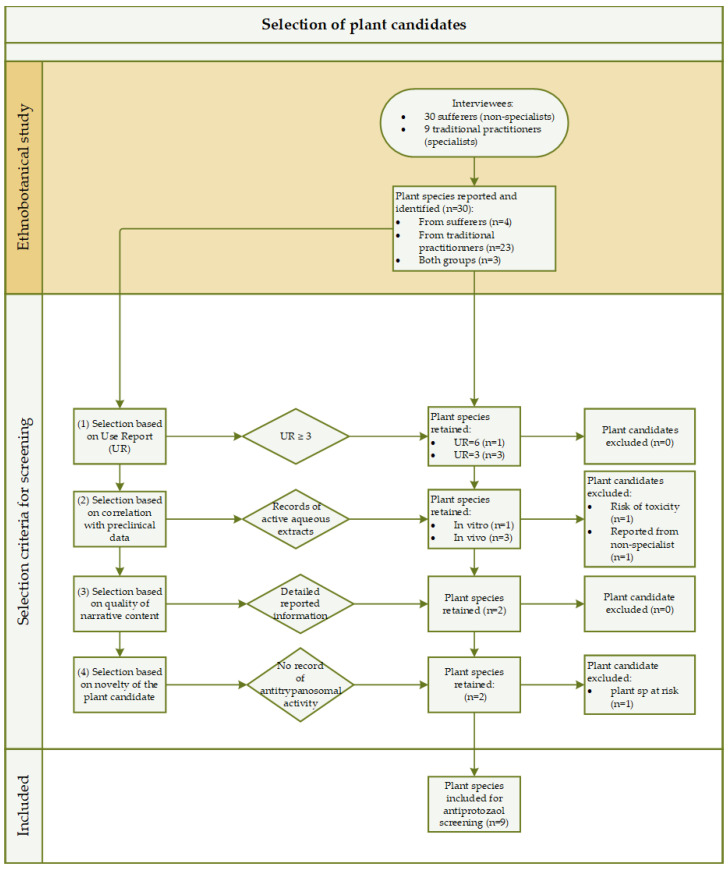
Flow diagram of the plant candidate selection process. Information in the horizontal corridor “ethnobotanical study” (highlighted in brown) arises from a previous ethnobotanical study run in Angola between 2017 and 2018, where 30 plant species had been identified [8]. Four inclusion criteria were applied (1) to (4). A total of 3 exclusion criteria were applied: potential risk of use (*n* = 1), reported from non-specialist informants (*n* = 1), and conservation status at risk (*n* = 1). Twelve plant species included: *Crossopteryx febrifuga*, *Vitex madiensis, Palisota schweinfurthii, Momordica charantia*, *Entada abyssinica*, *Sarcocephalus latifolius*, *Ocimum gratissimum, Securidaca longipedunculata*, *Nymphaea lotus*, *Brasenia schreberi*, *Brillantaisia owariensis* and *Daniellia alsteeniana*. Three plant species excluded: *Securidaca longipedunculata*, *Ocimum gratissimum* and *Daniellia alsteeniana*.

In a previous ethnobotanical study, 30 species of medicinal plants had been identified in the management of sleeping sickness in Angola [8]. Pursuing the aim to further investigate the studied plants as potential candidates for this disease, we selected nine species for pharmaco-chemical investigation. The plants were selected based on four criteria: the Use Report (UR), the correlation between traditional reported preparation and clinical data, the quality of the narrative content, and the novelty of the plant (see Figure 1).

The selected plants are summarized in Table 1.

### 2.2. Screening of Extracts against Trypanosoma brucei rhodesiense

A total of 122 extracts were prepared from different parts of the nine plant species. Each plant part was extracted consecutively with hexane, dichloromethane, ethanol, methanol, and water. The extracts were tested for their in vitro growth inhibition (GI) activity against bloodstream forms of *Trypanosoma brucei rhodesiense* STIB900, our reference strain for drug testing against African trypanosomes at a concentration of 20 µg/mL. Of the 122 extracts, 16 showed a strong activity (GI of 91–100%), 13 extracts a marked activity (71–90% GI), 14 extracts a moderate activity (51–70% GI), 19 extracts a weak activity (31–50% GI) and 60 extracts were inactive (GI < 30%). A detailed description of the plant species, the parts extracted, solvent, extraction yield, and percentage of growth inhibition (GI%) is given in Supplementary Table S1.

Only one of the nine investigated plants lacked inhibitory activity, *P. schweinfurthii*, whereas all other plants demonstrated at least one extract with a moderate antitrypanosomal activity. To the best of our knowledge, *B. schreberi* (Table S1, extracts ID 96, 98, 109, 110, 111) is reported for its antitrypanosomal activity for the first time here. Previous reports of the in vitro activity of *C. febrifuga* (leaves parts) [20], *S. latifolius* (root parts) [21], *E. abyssinica* (root parts) [22,23,24] could be correlated to the obtained results. An in vivo study had provided promising results with a 70% methanol extract of *N. lotus*, reducing the parasitemia in mice infected with *T. b. brucei* at a dose of 100 mg/kg/day [25]. However, here the 70% methanol extract of *N. lotus* only showed a moderate in vitro inhibitory activity (Table S1, extract ID 89, 114). Furthermore, the methanolic extract of *V. madiensis* (leaves) and *M. charantia* (aerial parts) were found to be weakly active or inactive with a growth inhibition lying between 7–35%, whereas two studies demonstrated an interesting in vitro activity [20,26]. Such a variation among activity results could be attributed to a combination of genetic, environmental, physiological, and methodological factors influencing the chemical composition and bioactivity of the plant extracts from different varieties of the same species. The latter was reflected by the difference in inhibitory activity within the same extract type of three different varieties of *N. lotus* collected from three different sites at different times (see Table S1, extracts IDs 88–94, 112–118, 110–121). Thus, *N. lotus* methanolic extracts IDs 89 and 114 exhibited a moderate activity, in contrast to the methanolic extract ID 121, which was inactive.

15 active extracts from seven different species displayed a growth inhibition activity > 70% at 20 µg/mL and were selected for further analysis (Table 2). Aside from activity, other considerations such as polarity and plant parts were also taken into account for the selection of the extracts. As cross-activities can be observed between different protozoa, the selected extracts were also tested against *Trypanosoma cruzi*, *Leishmania donovani*, and *Plasmodium falciparum*. In vitro 50% inhibitory concentrations (IC_50_) and selectivity indices (SI) were determined (Table 3). In general, the extracts were more active against *T. b. rhodesiense* and *P. falciparum* than against *T. cruzi* and *L. donovani*. All the extracts had selectivity indices > 1 for *T. b. rhodesiense* and *P. falciparum*. However, none of the extracts exhibited a high selectivity, which is not unusual due to the heterogeneous composition of the crude extracts. Further purification and isolation of the active constituents may highly improve the selectivity, as will also become apparent here.

The aqueous and ethanol 80% extracts of *E. abyssinica* root (extracts IDs 46, 47, 54) showed antitrypanosomal activity and the aqueous extract (extract ID 46) exhibited the most potent IC_50_ value against *T. b. rhodesiense* with 1.8 µg/mL. This is in agreement with Freiburghaus et al. [22], who had demonstrated similar in vitro activity for the root methanolic extracts of *E. abyssinica* harvested at two different periods (IC_50_ of 3.3 and 6.8 µg/mL vs. 4.1 µg/mL for ID 47, Table 3). Due to the several phytochemical studies already realized on this plant [23,24,27,28,29], we concentrated our efforts on *B. schreberi* (extracts IDs 109, 110, 111) and *N. lotus* (extracts IDs 91, 92), which displayed IC_50_ values ≤ 10 µg/mL against *T. b. rhodesiense* and *P. falciparum* (Table 3) and whose antitrypanosomal activity had remained mostly unexplored.

*B. schreberi* is a floating-leaf plant originating from North America and distributed throughout Africa, Asia and Australia. It has so far not been investigated for its antitrypanosomal activity. *B. schreberi* is used in a traditional preparation in combination with *N. lotus* in the management of sleeping sickness in Angola. Both are aquatic plants, and the invasiveness of *B. schreberi* makes it a competitor to *N. lotus* in its natural environment (Figure 4). Regarding its genus, two studies investigated the antitrypanosomal activity in the Nymphaeaceae. The first is from Nigeria and reported antitrypanosomal activity of *Nymphaea odorata* with an IC_50_ value < 5 µg/mL against *T. b. brucei* [30]. The second demonstrated in vivo antitrypanosomal potency for *N. lotus* [25]. However, no active molecules responsible for this activity have been described so far from this plant.

We first selected two midrange polarity extracts for further chemical investigation: the ethanolic extract (extract ID 110, Table 3) of the leaves of *B. schreberi* (It has to be clarified that in case of *B. schreberi* the leaves without petiole were extracted and tested, whereas for *N. lotus*, leaves and petiole were tested. In both cases, the plant part is referred to as “leaves”) (IC_50_ = 7.1 ± 4.6 µg/mL) and the dichloromethane extract (extract ID 116, Table 3) of the leaves and leaflets of *N. lotus* (IC_50_ = 12.2 ± 4.6 µg/mL). Then, we used a semi-preparative chromatography-based bioactivity-guided fractionation to tentatively identify the active constituents.

### 2.3. Isolation of Active Constituents from Brasenia schreberi J.F.Gmel and Nymphaea lotus L.

**Figure 2:**
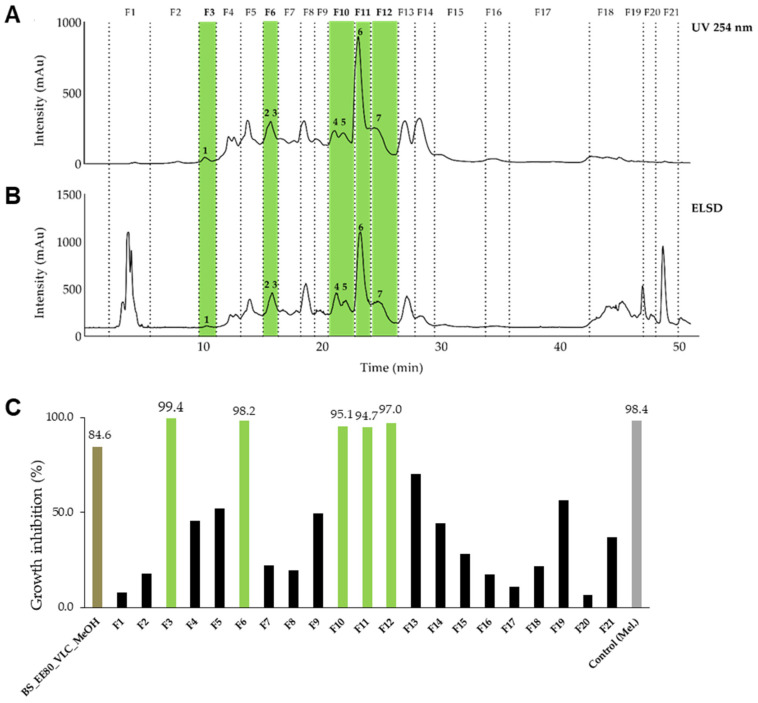
Semi-preparative HPLC chromatogram of the enriched methanolic extract of *B. schreberi* leaves with the collected fractions (F1 to F21) and the seven constituents 1 to 7. The separation of the components was detected by UV (**A**) and evaporative light scattering detectors (ELSD, (**B**)). The fractions were pooled according to UV and ELSD peaks. (**C**) Inhibitory activity of the VLC methanolic fractions against *T. b. rhodesiense* at 10 µg/mL. Five fractions (F3, F6, F10, F11, F12; green) displayed a strong activity (GI > 91%). BS_EE80_VLC_MeOH: enriched VLC methanolic extract of the ethanolic extract of *B. schreberi* (80%). Control: melarsoprol at 0.072 µg/mL (Mel.).

The 80% ethanol crude extract of *B. schreberi* leaves (extract ID 110) was first submitted to vacuum liquid chromatography (VLC) to remove the highly polar constituents (Figure S1). The VLC methanolic fraction (BS_EE80_VLC_MeOH) had demonstrated the most promising antitrypanosomal activity, with a GI value of 84.6% at 10 µg/mL (Figure S2) and was selected for fractionation. To optimize the semi-preparative fractionation, the analytical conditions were first determined by HPLC and the conditions were then geometrically transferred to the semi-preparative HPLC with a gradient transfer method [31]. The fractions were pooled according to UV and ELSD peaks (Figure 2A,B). In total, 21 fractions were collected and assayed against *T. b. rhodesiense*. Finally, five fractions (F3, F6, F10, F11, F12) displayed a strong activity (GI% > 91% at 10 µg/mL), markedly stronger than the VLC methanolic extract itself (Figure 2C and Figure S2).

**Figure 3:**
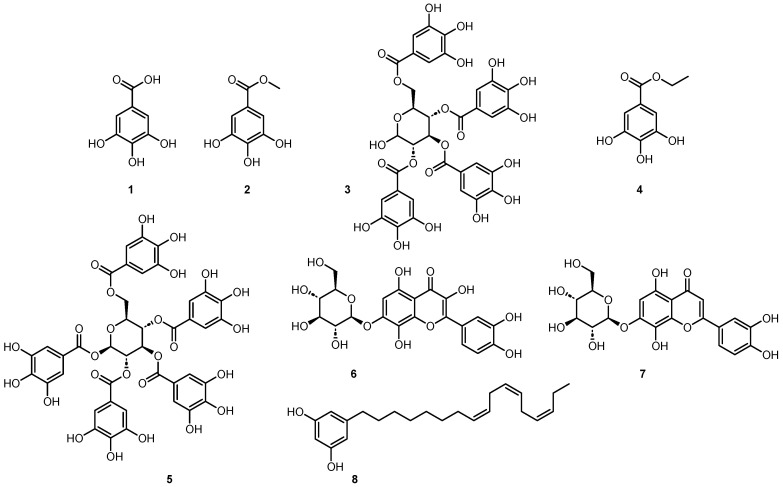
Structure of the identified compounds: gallic acid (**1**), methyl gallate (**2**), 2,3,4,6 tetragalloyl-glucopyranoside (**3**), ethyl gallate (**4**), 1,2,3,4,6 pentagalloyl-β-glucopyranoside (**5**), gossypetin-7-*O*-β-glucopyranoside (**6**), hypolaetin-7-*O*-glucoside (**7**), 5-[(8Z,11Z,14Z)-heptadeca-8,11,14-trienyl] resorcinol (**8**).

Fractions F3 and F11 yielded two single compounds, **1** and **6**. Fractions F6, F10, F12 were further purified using semi-preparative HPLC and yielded five minor compounds, **2** to **5** and **7** (Figure S3). NMR and high-resolution MS analysis resulted in identification of the seven active constituents, namely, gallic acid (**1**) [32], methyl gallate (**2**) [32], 2,3,4,6 tetragalloyl-glucopyranoside (**3**) [33] ethyl gallate (**4**) [34], 1,2,3,4,6 pentagalloyl-β-glucopyranoside (**5**) [35], gossypetin-7-*O*-β-glucopyranoside (**6**) [36], and hypolaetin-7-*O*-glucoside (**7**) [37] (Figure 3 and Figures S4–S11).

Gallic acid **1** and its ester derivatives, compounds **2** to **5**, are common natural polyphenols, widely present in plants and fungi. These secondary metabolites are known for a range of applications [38] and possess several activities such as antioxidant and neuroprotective [39], anti-inflammatory [40], anti-tumor [41,42,43,44], and anti-bacterial ones [45,46,47]. Among the compounds studied, three **1**, **4**, and **7** had already been described from *B. schreberi* [48,49] as well as gossypetin, the aglycone of compound **6**. This compound is predominantly present in the genus *Hibiscus* and has been isolated in many other plant species, like *Drosera peltata* [50] or *Equisetum fluviatile* [51]. However, the presence of compounds **2**, **3**, **5**, and **6** in the genus *Brasenia* is reported for the first time here.

**Figure 4:**
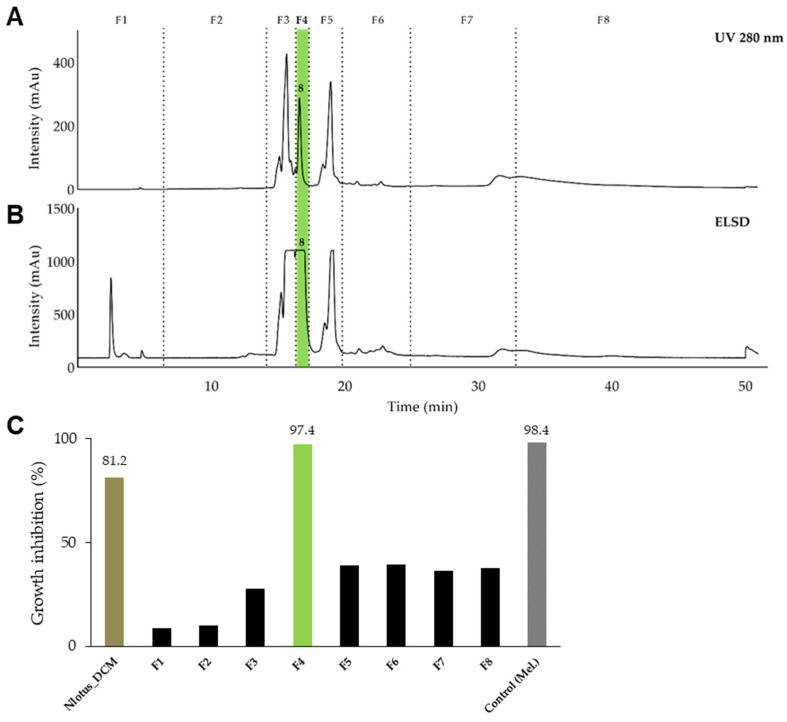
Semi-preparative HPLC chromatogram of the dichloromethane extract of *N. lotus* highlighting the collected fractions F1 to F8 and the active constituent **8**. The separation of the components was detected by UV (**A**) and evaporative light scattering detectors (ELSD, (**B**)). (**C**) Inhibitory activity against *T. b. rhodesiense* at 10 µg/mL of the fractions. Only one fraction (F4, green) displayed a strong activity (97%). Legend: Nlotus_DCM: dichloromethane extract of *N. lotus*. Control: melarsoprol (Mel.) at 0.072 µg/mL.

The dichloromethane extract of the leaves and leaflets of *N. lotus* (extract ID 116) was fractionated by normal phase semi-preparative chromatography using the same method as described previously but in normal phase. The 62 fractions generated were combined according to their UV and ELSD peaks (Figure 4A,B) in eight fractions (F1–F8) and assayed against *T. b. rhodesiense* (Figure 4C). One active fraction (F4) demonstrated a strong activity (GI% 97.4% at 10 µg/mL). Analysis of fraction F4 revealed a single constituent structurally elucidated by NMR and high-resolution MS, and identified as a known alkenyl resorcinol (**8**) [52] (Figure 3). The resorcinolic lipids have been associated with plants, bacteria and fungi [53]. They are mostly found in the members of families *Anacardiaceae* (e.g., cashew, mango), *Ginkgoaceae* (e.g., Ginkgo biloba) and *Graminaceae* (e.g., cereals) [54]; to the best of our knowledge, occurrence in the *Nymphaeaceae* is reported here for the first time. *Nymphaea odorata* was described in a study on Nigerian medicinal plants for its activity against *T. b. brucei* [30]. The structures of the resorcinol **8** identified from *N. lotus* and the seven active constituents identified from *B. schreberi* **1**–**7** are shown in Figure 3.

### 2.4. Antiprotozoal Activity of the Identified Components

Except compound **3**, for which we had insufficient plant material, the identified compounds were evaluated against *T. b. rhodesiense* and other protozoa. Compounds **1**, **2**, **4** and **5** could be purchased and were assayed against *T. cruzi*, *L. donovani* and *P. falciparum* (Table 4). Compounds **6**, **7** and **8** were assayed only against *T. cruzi* and *L. donovani* (Table 4). Ethyl gallate **4** and methyl gallate **2** had IC_50_ values against *T. b. rhodesiense* of 0.6 µg/mL and 1.1 µg/mL, respectively, as well as of 2.1 µg/mL and 3.0 µg/mL against *P. falciparum*. The highest antitrypanosomal activity was found for gallic acid **1** and ethyl gallate **4,** with IC_50_ against *T. b. rhodesiense* of 0.5 µg/mL and 0.6 µg/mL, respectively. None of the compounds demonstrated promising activity against *T. cruzi*. Resorcinol alkyl **8** had an IC_50_ of 2.5 µg/mL against *L. donovani* and a moderate selectivity (SI: 5.2). The two glycosidic flavones **6** and **7** displayed similar activities across the three trypanosomatides. The glucuronate flavones were less potent than their aglycones [55], suggesting that the antitrypanosomal activity of compounds **6** and **7** could be improved by removing the glycosidic part. The gallotannin pentagalloyl glucose **5** displayed the weakest overall antiprotozoal activity.

Our findings are in agreement with the reported activity of gallic acid **1** and ethyl gallate **4** against bloodstream forms of *T. b. brucei* [56,57]. Gallic acid and its ester derivative **2** inhibited the sn-glycerol-3-phosphate oxidase system of *T. b. brucei* in vitro [58]. Another possible mechanism of action of gallic acid is via its capacity to chelate iron and deprive the parasite [59,60]. Due to the amphiphilic nature of alkyl esters **2** and **4**, these compounds might disrupt the plasma membrane, leading to trypanosome death [61]. Yet another possible target is the trypanosome alternative oxidase TAO; intriguingly, *T. brucei* spp. aquaglyceroporin-null mutants, which are resistant to the drugs melarsoprol and pentamidine, are at the same time hypersensitive to inhibitors of TAO, including octyl gallate and propyl gallate [62].

The results with amastigote *T. cruzi* are consistent with a previous finding that gallic acid and two of its ester derivatives **2** and **4** were inactive (IC_50_ > 100 µM) against epimastigote *T. cruzi* [63]. The detected antiplasmodial activity of compounds **2** and **4** were higher than previously reported [64]. However, another study had demonstrated a strong in vitro activity against *P. falciparum* for methyl gallate **2** (IC_50_ of 2.5 ng/mL) isolated from *Alectryon serratus* leaves [65] and an IC_50_ of 1.3 ng/mL for gallic acid **1**. The finding that methyl gallate **2** and ethyl gallate **4** have a higher antiplasmodial activity than gallic acid **1** itself is corroborated by a previous report [66]. The gallotannin pentagalloylglucose **5** has demonstrated several biological activities [67]. The antileishmanial activity obtained here was, however, lower than in a previous report [68]. The resorcinol alkyl **8** displayed an encouraging inhibitory activity when tested against axenic amastigotes of *L. donovani* (IC_50_ of 2.5 µg/mL). However, it did not demonstrate conclusive activity when tested in an intramacrophage assay (IC_50_ > 11 µg/mL). Interestingly, an isomer of compound **8**, 5-heptadeca-8′Z,11′Z,16-trienylresorcinol, was isolated from the mushroom *Merulius incarnatus* and had a similar activity against leishmania (IC_50_ of 3.6 µg/mL) [69], as found here. The saturation degree of the alkyl chain impacts the bioactivity, resulting in loss of activity when saturated [69] as well as the stereochemical orientation of the double bond system. In addition to the presence of unsaturation in the alkyl chain, a free phenolic hydroxyl group is required for bioactivity of resorcinol alkyls [70,71]. Besides its interesting activity against leishmania, compound **8** displayed the best activity against *T. cruzi* among the isolated constituents (IC_50_ of 9.1 µg/mL). This is in agreement with Matutino Bastos et al. (2019), who had assayed two derivates of cardol against *T. cruzi* trypomastigote and amastigote forms [72]. Our results, together with these previous findings, ask for further investigation on resorcinol alkyls as potential compounds against *L. donovani* and *T. cruzi*.

### 2.5. Active Constituents in Local Herbal Preparation

**Figure 5:**
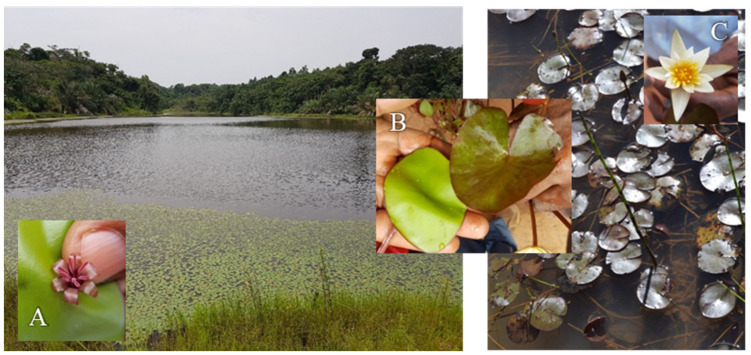
*Brasenia schreberi* (**left**) and *Nymphaea lotus* (**right**) in their natural environment in Angola, province of Uíge. Insets show the leaves ((**B**), *B. schreberi* left and *N. lotus* **right**) and flowers ((**A**), *B. schreberi* “female”; (**C**), *N. lotus* “male”). The two species are collected together, prepared as a decoction, and administered in the management of sleeping sickness.

In the northern province Uíge of Angola, the ethnotaxon “Longa dia simbi” is used for the treatment of sleeping sickness in the form of a decoction. “Longa dia simbi” in the local language Kikongo means “a tray”, referring to the leaves lying as a tray on the surface of the water. “Longa dia simbi” is made of *Brasenia schreberi* and *Nymphaea lotus*. The two species are considered by local traditional healers as the same plant; *B. schreberi* as “female” and *N. lotus* as “male” (see Figure 5). To validate the potential antitrypanosomal activity of the traditional preparation, the crude extracts of the decoction of *B. schreberi* (the leaves) and of *N. lotus* (leaves and leaflets) were analyzed by Ultra High-Performance Liquid Chromatography (UHPLC-MS) to detect the presence of the previously identified active constituents.

Resorcinol alkyl, the major active component of *N. lotus*, was not detected in the decoction (extract ID 112, Table S1), which was to be expected given the lipophilic structure of this compound. Nevertheless, the presence of compounds **1**, **4**, **6** and **7** was confirmed by LC-MS and UV analysis. These four constituents can account for the observed in vitro activity (GI% value 31–50%; Table S1) of the aqueous extract of *N. lotus* (ID 112) against *T. b. rhodensiense*. The combined UV-MS detection and analysis of *B. schreberi* decoction (extract ID 109) revealed the presence of five active components, **1**, **2**, **5**, **6** and **7**. These findings confirm the first activity screening, where the decoction extract of the leaves of *B. schreberi* (ID 109) displayed a strong inhibitory activity against *T. b. rhodesiense* (GI% value > 91%; see Table S1). A quantification of the identified active compounds **1**, **2**, **4**, and **5** was realized by UHPLC-UV Single Quadrupole MS analysis using pure reference substance (Table 5).

## 3. Materials and Methods

### 3.1. Chemicals

LC/MS-grade acetonitrile was obtained from VWR International (Dietikon, Switzerland), and formic acid (99%) via Thommen-Furler AG (Rüti b. Büren, Switzerland) from Carlo Erba Reagents. Ultrapure water was obtained from an in-house ultrapure water system from Sartorius AG. The reference compounds gallic acid **1**, methyl gallate **2**, and ethyl gallate **4** were purchased from Sigma-Aldrich; 1,2,3,4,6-pentagalloyl-β-glycopyranoside **5** was obtained via Lucerna-Chem from MedChem Express. The reference compounds used as positive controls for drug efficacy testing were melarsoprol (Arsobal Sanofi-Aventis, received from WHO, Geneva, Switzerland), benznidazole (Epichem Pty Ltd., received from DNDi, Geneva, Switzerland), miltefosine (Sigma Aldrich, Buchs, Switzerland), chloroquine (Sigma Aldrich, Buchs, Switzerland), artesunate (Mepha Pharma AG, Aesch, Switzerland), and podophyllotoxin (Sigma Aldrich, Buchs, Switzerland).

### 3.2. Selection of the Candidate Plants

In an ethnobotanical study led by the authors over a 5-month period, from October 2016 to March 2017 in endemic regions of Angola, 30 plant species used in the management of sleeping sickness had been identified [8]. The data gathered through the ethnobotanical study together with a literature review on antitrypanosomal activity of the candidate plants provided information as pre-screen to select the plants for further pharmacological investigations. A total of 9 species were selected according to four inclusion criteria and three exclusion criteria (see Figure 1).

The four inclusion criteria were (1) the Use Report (UR), (2) the correlation between traditional reported preparation and clinical data, (3) the quality of the narrative content, and (4) the novelty of the plant. Briefly, the UR enabled quantifying the importance of the reported plant species among the interviewees. A higher UR value indicates that a plant species is more frequently mentioned within the informants, suggesting its greater importance or relevance to the cultural practices. Plant candidates with a UR ≥ 3 were retained (*n* = 4, namely *Crossopteryx febrifuga* (Afzel.ex G.Don) Benth, *Vitex madiensis* Oliv., *Palisota schweinfurthii* C.B.Clarke, and *Momordica charantia* L.). Based on a correlation between the traditionally reported herbal preparation (most often a decoction) and the preclinical results (in vitro and in vivo studies) of the extracts that mimicked most closely the traditional preparation (aqueous extracts), four plant species were included (*n* = 4, namely *Entada abyssinica* A.Rich., *Sarcocephalus latifolius* (Sm.) E.A.Bruce, *Ocimum gratissimum* L., and *Securidaca longipedunculata* Fresen). The quality of the narrative content referred to a qualitative appreciation of the information entrusted during the interview with the informant such as (a) the level of a detailed information, (b) a same response to a repeated question, (c) the veracity of the facts and (d) a collaborative attitude. Based on this parameter, one species was retained (*n* = 1, namely *Nymphaea lotus* L.). However, as explained under 2.5, *N. lotus* relates to the ethnotaxon “Longa dia simbi” that referred to two plant species, *N. lotus* and *B. schreberi* J.F. Gmel. Therefore, two plant candidates had to be considered for this third inclusion criteria (*n* = 2, namely *Nymphaea lotus* L. and *Brasenia schreberi* J.F. Gmel). The novelty criterion included plants species with an UR = 2 that had not been so far investigated for their antitrypanosomal activity (*n* = 2, namely *Brillantaisia owariensis* P.beauv. and *Daniellia alsteeniana* P.A.Duvign).

Three exclusion criteria were applied: (a) plant species known for their potential risk of use (*n* = 1, namely *Securidaca longipedunculata* Fresen.), (b) plant candidates reported from non-specialist informants (sufferers of trypanosomiasis) (*n* = 1 namely *Ocimum gratissimum* L.) or (c) species with a conservation status at risk (*n* = 1 namely *Daniellia alsteenianaP.A.Duvign*) [73].

### 3.3. Plant Collection, Identification and Exportation

The plant material was collected in the northern province Uíge of Angola. The nine plant species were authenticated by the Center of Studies and Scientific Investigation on Botanic of the Faculty of Science from University of Agostinho Neto, Luanda, Angola. The corresponding voucher specimen were deposited at the herbarium of the Center of Studies and Scientific Investigation on Botanic (Table 1).

Nagoya clearance was obtained by establishing a “Mutually Agreed Terms” (MAT) between the involved Parties. The MAT granted in the framework of this collaboration in July 2018, was the first official MAT of Angola, as has been notified in the Interim National Report on the Implementation of the Nagoya Protocol published on May 2019 on the platform of the ABS-Clearing House “https://absch.cbd.int/countries/AO (accessed on 15 February 2024)”.

### 3.4. Extract Preparation

Different types of extraction procedure were carried out on the powdered dry material. A detailed description of plant species, parts extracted, solvents, drug-solvent ratio, and extraction yields is given in Table S1.

For increasing polarity extraction, the plant material was successively extracted for 18 ± 2 h at room temperature under constant stirring with hexane, dichloromethane (DCM), methanol (MeOH), and distilled water (H_2_O). After filtration, the extracts were evaporated under vacuum (Büchi Rotavapor, Büchi, Switzerland) and dried under nitrogen stream. The solvent-free extracts were stored at 4 °C until use.

To replicate traditional preparations, a 20-fold quantity of water in relation to plant material was used for the extraction and boiled for 15 min. The decoction was filtrated with a Büchner funnel under vacuum or with a filter paper (Macherey-Nagel, Düren, Germany). The filtrates (AqDec) were freeze-dried and stored at 4 °C until use. Additionally, a 10% ethanolic extract (MetT) was produced by maceration at room temperature for 2 h. Filtration and drying were performed similarly as for the decoction.

For alcoholic extraction, an 80% ethanol extract (EtOH80%) was prepared by adding a ten-fold quantity of solvent in relation to plant material and extracted at room temperature for 2 h under constant agitation. Extracts were filtered through a filter paper (Macherey-Nagel), concentrated on a rotavapor (Büchi, Switzerland) at 40 °C until 60 mbar, freeze-dried, and stored at 4 °C until use. In order to assess previously referenced activity of some plant species, the extraction procedure was reproduced as published (MeOH70%, AqMac, MeOH80%).

### 3.5. General Chromatographic Procedures

NMR spectroscopic data were recorded on a Bruker Avance III HD 600 MHz NMR spectrometer equipped with a QCI 5 mm Cryoprobe and a SampleJet automated sample changer (Bruker BioSpin, Rheinstetten, Germany). Chemical shifts (δ) were measured in parts per million (ppm) using the CD_3_OD signal as internal standard for all spectra (δH 3.31; δC 49.0), and coupling constants (J) are reported in Hz. Complete assignment was performed based on two-dimensional experiments (COSY, NOESY, HSQC and HMBC). High-resolution tandem mass spectrometry (HRMS/MS) data were obtained on a Q Exactive Focus quadrupole-orbitrap mass spectrometer (Thermo Scientific, Bremen, Germany) using heated electrospray ionization (HESI-II) in the positive and negative modes. Reverse and normal phase analysis were performed on a high-performance liquid chromatography (HPLC) Agilent 1260 Infinity LC and Agilent 1100 series system, respectively, both consisting of a degasser, a mixing pump, an autosampler, and a diode array detector (DAD) (Agilent Technologies, Santa Clara, CA, USA) connected to an evaporative light scattering detector (ELSD) Sedex LT-ELSD 85 or ELSD Sedex 55 (Sedere, Alfortville, France) to detect non-UV absorbing compounds. Fractionation of the enriched ethanolic extract of *B. schreberi* (BS_EE80_VLC_MeOH) and the dichloromethane extract of *N. lotus* (NLotus_DCM) were performed on a semi-preparative HPLC equipment (Armen modular spot prep II, Saint-Avé, France) connected to a ELSD Sedex 55 (Sedere, Alfortville, France). Sub-fractions of the VLC_MeOH extracts of *B. schreberi* were purified with a Shimadzu system equipped with a LC-20 A module pumps, an SPD-20 A UV/VIS detector, a 7725I Rheodyne^®^ valve and an FRC-10 A fraction collector (Shimadzu, Kyoto, Japan).

### 3.6. Fractionation and Isolation of Active Constituents

The 80% ethanolic extract of *B. schreberi* leaves and the dichloromethane extract of *N. lotus* leaves were fractionated and purified in order to isolate eight active constituents. The ethanolic extract of *B. schreberi* was first subjected to a vacuum liquid chromatography (VLC) to remove very polar compounds. A 500 mL sintered-glass Büchner funnel attached to a vacuum line was packed with a C18 reverse phase Zeoprep^®^ 40–63 μm (Lobar^®^ Merck, Darmstadt, Germany), activated with methanol (4 × 250 mL) and equilibrated with distilled water (4 × 250 mL). The dry load composed of 3.53 g of the grinded extract mixed with the same stationary phase (1:1 *w*/*w*) was then loaded uniformly on the top of the stationary phase. The sample was eluted using water (6 × 250 mL) followed by methanol (6 × 250 mL) and washed with ethyl acetate (6 × 250 mL). The water fraction was lyophilized, while the methanol and ethyl acetate fractions were evaporated, to yield BS_EE80_VLC_H_2_O (2.5 g), BS_EE80_VLC_MeOH (2.6 g) and BS_EE80_VLC_EtOAc (85 mg), respectively. The optimized analytical conditions for BS_EE80_VLC_MeOH were determined by HPLC as a step gradient from 5% to 14% of B in 5 min, then 14% to 30% of B in 5 min, 30% to 60% of B in 30 min and 60% to 100% of B in 5 min held during 10 min. Then, a geometrical gradient transfer was applied from analytical to semi-preparative scale using chromatographic calculations to ensure the same selectivity. The fractionation was performed on 80 mg of the extract (BS_EE80_VLC_MeOH) on a semi-preparative HPLC system (Armen modular spot prep II, Saint-Avé, France) using an Interchim C18 column (250 × 21.2 mm, 10 μm; Interchim, Montluçon, France), with water (A) and methanol (B) containing 0.1% formic acid as mobile phase. The purification was performed using the same step gradient as the analytical conditions with a flow rate fixed at 17 mL/min. The UV detection was set at 254 nm and ELSD detection was performed under the following conditions: 40 °C, 3.1 bar N_2_ and gain 8. The separation led to 61 fractions combined in 21 fractions according to their UV and ELSD signal (Figure 1A,B). All fractions were evaporated and submitted to the in vitro growth inhibition assay (Figure 1C). Fractions F3 and F11 exhibited an activity, and compounds **1** (0.7 mg) and **6** (4 mg) were identified as major compounds of these two fractions. The fractions F6, F10 and F12, which displayed an activity but could not be identified, were further purified on a Shimadzu semi-preparative equipment using a X-bridge C18 column (250 × 10 mm, 5 μm; Waters, Milford, MA, USA), with water (A) and methanol (B) containing both 0.1% formic acid as mobile phase. The purification of F6, F10 and F12 was performed using a step gradient from 17% to 25% of B in 60 min, held during 10 min. Briefly, F6 (5.4 mg), F10 (6.5 mg) and F12 (3.6 mg) were dissolved separately in 300 μL of methanol, added to a spatula of Zeoprep C18 silica (40–63 μm) and dried gently under N_2_ stream. The mixture was loaded in a cartridge for dry load injection according to the method developed by Queiroz et al. [31]. The flow rate was fixed at 5 mL/min. The UV detection was set at 254 nm (F12) and 280 nm (F6, F10). The separation led, respectively, to 27 sub-fractions for F6, 21 for F21and 12 for F12. Sub-fractions were combined according to their UV detections (Figure S3). Using this approach, compound **2** (0.1 mg) and **3** (0.1 mg) from F6, compound **4** (0.1 mg) and **5** (0.5 mg) from F10, and compound **7** (0.6 mg) from F12 were isolated.

The dichloromethane extract of *N. lotus* was fractionated on a semi-preparative system (Armen modular spot prep II, Saint-Avé, France) using an Interchim SIHP column (21.2 × 250 mm, 10 μm; Interchim, Montluçon, France) equipped with a Universal Guard Selectivity (UGS) SI pre-column cartridge holder (3 × 6 mm i.d., 10 µm); with hexane (A) and ethyl acetate (B) as mobile phase. The purification was performed using a linear gradient from 5% to 100% of B in 40 min, held during 10 min. The flow rate was fixed at 17 mL/min, the UV detection at 280 nm. This fractionation led to 62 fractions combined in 8 fractions according to their UV detection (Figure 2A,B). Using this approach, compound **8** (1.6 mg) was isolated from F4. The fraction was evaporated and submitted to the in vitro growth inhibition assay (Figure 2C).

The four isolated compounds **3**, **6**, **7**, **8** tested for their antitrypanosomal activity had their identity confirmed by MS data and NMR spectra, which were in accordance with published data [33,36,37,52]. The purity of the compounds was estimated by ^1^H-NMR and found to be >80% in all cases.

### 3.7. UHPLC_-HRMS/MS Analysis

UHPLC-HRMS/MS analysis was performed for the active extracts and pure compounds using a Waters^®^ Acquity UPLC system connected to a Q Exactive Focus mass spectrometer (Thermo Scientific, Bremen, Germany) with a heated electrospray ionization (HESI-II) in the positive and negative modes. The optimized HESI-II parameters were as follows: source voltage, 3.5 kV (pos), 3.8 kV (neg); sheath gas flow rate (N_2_), 55 units; auxiliary gas flow rate, 15 units; spare gas flow rate, 3.0; capillary temperature, 275 °C (pos), 320 °C (neg); S-Lens RF Level, 45. The mass analyzer was calibrated using a mixture of caffeine, methionine–arginine–phenylalanine–alanine–acetate (MRFA), sodium dodecyl sulfate, sodium taurocholate and Ultramark 1621 in an acetonitrile/methanol/water solution containing 1% formic acid by direct injection. The data-dependent MS/MS events were performed on the four most intense ions detected in full scan MS (Top 3 experiment). The MS/MS isolation window width was 1 Da, and the normalized collision energy (NCE) was set to 35 units. In data-dependent MS/MS experiments, full scans were acquired at a resolution of 35,000 FWHM (at m/z 200) and MS/MS scans at 17, 500 FWHM both with a maximum injection time of 50 ms. After being acquired in a MS/MS scan, parent ions were placed in a dynamic exclusion list for 2.0 sec. Separation was achieved on an Acquity BEH C18 column (2.1 × 50 mm; 1.7 µm; Waters, Milford, MA, USA) with water (A) and acetonitrile (B) as the mobile phase. The temperatures in the autosampler and in the column oven were fixed at 25 and 40 °C, respectively. Separation was performed with a linear gradient from 5% to 95% of B in 7 min, held during 1 min and then 1 min isocratic step at 5% of B for column reconditioning. The injection volume was set to 2 µL, the flow rate was fixed at 0.6 mL/min. An Acquity UPLC photodiode array detector (PDA) was used to acquire PDA spectra, which were collected from 210 to 450 nm. In positive ion mode, the di-isooctyl phthalate C_24_H_38_O_4_ [M + H]^+^ ion (*m/z* 391.28429) was used as an internal lock mass.

### 3.8. HPLC-DAD-ELSD Analysis

The extracts of *B. schreberi* were analyzed by HPLC with DAD and ELSD detection on an Interchim C18 column (250 × 4.6 mm i.d., 10 μm; Interchim, Montluçon, France) equipped with a Nova-Pak^®^ C18 pre-column cartridge holder (4 µm, 60 Å), using a mobile phase consisting of water (A) and methanol (B) containing both 0.1% formic acid; separation was performed with a linear gradient from 5% to 100% of B in 40 min, held during 5 min; flow rate: 1 mL/min; injection volume: 10 μL. The samples were diluted in methanol to 10 mg/mL. The UV detection was recorded at 210, 254, 280 and 366 nm. ELSD conditions: 45 °C, 3.5 bar N_2_ and gain 8.

The DCM extract of *N. lotus* was analyzed by normal-phase HPLC with UV and ELSD detections on a Interchim SIHP column (250 × 4.6 mm, 10 µm; Interchim, Montluçon, France) equipped with a Universal Guard Selectivity (UGS) SI pre-column cartridge holder (3 × 6 mm i.d., 10 µm) using a mobile phase consisting of hexane (A) and ethyl acetate (B); separation was performed as described in the previous paragraph except that the samples were diluted in ethyl acetate.

### 3.9. NMR Spectroscopic Data

The recorded spectroscopic data were compared with the ones available in the literature to identify unambiguously compound **1** as gallic acid [32], **2** as methyl gallate [32], **3** as a mixture of 2 tetragalloylglucose [33], **4** as ethyl gallate [34], **5** as 1,2,3,4,6-pentagalloyl-β-glucopyranoside [35], **6** as gossypetin 7-*O*-glucopyranoside [36], **7** as hypolaetin-7-*O*-glucoside [37] and **8** as an alkenyl resorcinol [52].

### 3.10. Quantification of Active Pure Compounds

The analysis was performed with an UHPLC-MS (UPLC with QDa detector, Waters) equipped with an Acquity column (BEH C18 2. 1 mm × 100 mm, 1.7 µm with the following parameters: mobile phase water/formic acid (1000:1 *v/v*) (A) and acetonitrile (B); flow rate 0.3 mL/min; column temperature 35 °C; temperature of the sample chamber 15 °C; injection volume 5 µL. The gradient used was set at 1% for 2 min, then 1–5% in 1 min, then 5–15% in 9 min held during 1 min, followed by 5 min from 15 to 95% maintained for 2.5 min. The analysis was carried out with the QDa detector in negative mode. The cone voltage was set to −15 V, ESI Capillary: 0.81 kV and the capillary temperature to 600 °C. The quantification was performed over their respective mass traces in SIR mode (selected ion recording): 169 Da (gallic acid), 183 Da (methyl gallate), 197 Da (ethyl gallate) and 469 Da (1,2,3,4,6 pentagalloyl-β-glucopyranoside).

Gallic acid was quantified with a PDA detector at 270 nm. A standard curve was used for the quantification from 0.240–245.7 mg/L (R^2^ = 0.9998). Methyl gallate, ethyl gallate and 1,2,3,4,6 pentagalloyl-β-glucopyranoside were quantified by UHPLC-MS with a QDa detector and standard curves were established at 0.004–0.132 mg/L (R^2^ = 0.9978), 0.094–94.080 mg/L (R^2^ = 0.9999), and 0.28–175 mg/L (R^2^ = 0.9993), respectively. Each sample was filtered (0.2 µm) and prepared at 1 mg/mL in distilled water.

### 3.11. Antiprotozoal Activity and Cytotoxicity Testing

Growth inhibition (GI) activity against *T. b. rhodesiense* STIB 900 was determined as follows: in a 96-well microtiter plate, 50 µL of HMI-9 medium supplemented with 15% heat-inactivated horse serum were added to each well. A total of 10 µL of the plant extract stock solution was added to each well. Then, 50 µL of bloodstream-form trypanosomes were added, adjusted with a cell counter (CASY, Schärfe System, Germany) to 4 × 10^4^ cells/mL. Another 50 µL of HMI-9 medium supplemented with 15% heat-inactived horse serum was added to each well of the microtiter plate. The final concentration of the tested extract was 20 µL/mL. The plate was incubated at 37 °C under a 5% CO_2_ atmosphere for 72 h. A total of 10 µL of Alamar blue solution (12.5 mg resazurin dissolved in 100 mL distilled water) was added to each well and the plate incubated for another 2 to 4 h. Then, the plate was read with a Spectramax Gemini XS microplate fluorometer (Molecular Devices Corporation, Sunnyvale, CA, USA) using an excitation wavelength of 530 nm and an emission wavelength of 590 nm. Fluorescence was expressed as percentage of the untreated control. A GI > 91% was considered a strong inhibitory activity, between 71–90% a marked activity, between 51–70% a moderate activity, between 31–50% as a weak activity, <30% as not active. IC_50_ determination was performed in a similar way, but with serial dilutions of the plant extract (or pure compound) covering a range from 90 to 0.123 µg/mL. IC_50_ values were calculated by linear interpolation selecting values above and below the 50% inhibition mark.

In vitro growth inhibitory activity of the extracts and pure compounds against *T. cruzi* (intracellular amastigote forms grown in L6 rat myoblasts), *L. donovani* (axenic amastigote forms in acidic medium or intracellular amastigotes in mouse primary macrophages), and *P. falciparum* (erythrocytic stages in culture) was determined as described previously [74]. Testing against intracellular *L. donovani* was performed as follows: mouse peritoneal macrophages (4 × 104 in 100 µL RPMI 1640 medium with 10% heat-inactivated FBS) in 96-well plates were infected with amastigotes (2 × 105 in 100 µL medium). After 24 h, the medium was exchanged twice to remove free amastigotes. Test compounds were added, and the plates incubated for 96 h at 37 °C under 5% CO_2_. Then, the medium was removed, the cells were fixed with 50 µL 4% formaldehyde, and stained with 5 µM DRAQ5. Nine images were taken per well on a ImageXpress XLS (MD) microscope (Molecular Devices, San Jose, CA, USA) using a 20× air objective (635 nm excitation: 690/50 emission) and analyzed with a script developed on Meta Xpress (MD). Cytotoxicity against L6 cells was assessed by using a similar protocol as outlined for IC_50_ determination with *T. b. rhodesiense*, except that rat skeletal myoblasts (L6 cells) were used. The medium was RPMI 1640 medium supplemented with 1% L-glutamine (200 mM) and 10% fetal bovine serum. Reference compounds were melarsoprol for *T. b. rhodesiense*, benznidazole for *T. cruzi*, miltefosine for *L. donovani*, chloroquine and artesunate for *P. falciparum*, and podophyllotoxin for L6 cells.

## 4. Conclusions

Aiming to provide preliminary safety and efficacy validation of traditional herbal preparations, we investigated the cytotoxicity and antitrypanosomal activity of different extracts from medicinal plants that are being used in Angola in the treatment of sleeping sickness. After a preliminary activity screening, 15 active extracts were retained. Two extracts of two different aquatic plants, *Brasenia schreberi* and *Nymphaea lotus*, displayed IC_50_ values ≤ 10 µg/mL. Interestingly, these two Nymphaeales are being used in combination in a traditional preparation for the management of sleeping sickness in the northern part of Angola. While this is the first investigation of their antitrypanosomal constituents, *B. schreberi* and *N. lotus* have been investigated for several other bioactivities such as antioxidant [75] and anti-inflammatory [48,76], anti-bacterial [77,78,79], anti-algal [80], anti-adipogenic [49] as well as lowering cholesterol [81] and inhibition of HIV-1 reverse transcriptase [82,83].

In the present study, we report on the bioactivity-guided fractionation of the dichloromethane extract of *N. lotus* and the VLC methanolic extract of *B. schreberi* with the identification of eight active constituents **1–8**. The presence of several antitrypanosomal compounds, gallic acid, methyl gallate, ethyl gallate and 1,2,3,4,6-pentagalloyl-β-glucopyranoside in the traditional preparation made of the leaves and leaflets of *B. schreberi* and *N. lotus* provides first evidence of the potential of the local preparation in the management of sleeping sickness in Angola. However, toxicity and in vivo efficacy remain to be further investigated.

## Figures and Tables

**Table 1:** Medicinal plants from Angola analyzed in this study. Collection number of the National Botanical Center in Luanda, Angola. n.d.: not determined.

Plant	Family	Collection Number
*Brillantaisia owariensis* P.beauv.	Acanthaceae	7925
*Brasenia schreberi* J.F. Gmel	Cabombaceae	n.d.
*Palisota schweinfurthii* C.B.Clarke	Commelinaceae	894
*Momordica charantia* L.	Cucurbitaceae	8591
*Entada abyssinica* A.Rich.	Fabaceae	3468
*Vitex madiensis* Oliv.	Lamiaceae	7186
*Nymphaea lotus* L.	Nymphaeaceae	2513
*Crossopteryx febrifuga* (Afzel.ex G.Don) Benth	Rubiaceae	8212
*Sarcocephalus latifolius* (Sm.)E.A.Bruce	Rubiaceae	8231

**Table 2:** The 15 most promising extracts and their activity against *T. b. rhodesiense*. GI, growth inhibition; Ri, rhizomes; R, roots; AeP, aerial parts; Rb, root barks; L, leaves; EtOH, ethanol; MeOH, methanol; DCM, dichloromethane.

Extract ID	Plant Name	Plant Part	Solvent	GI (%) ^1^
46	*E. abyssinica*	Ri	Aqueous	103
47	*E. abyssinica*	Ri	EtOH 80%	101
91	*N. lotus*	AeP	Hexane	98
54	*E. abyssinica*	Rb	EtOH 80%	98
109	*B. schreberi*	L	Aqueous	99
110	*B. schreberi*	L	EtOH 80%	96
111	*B. schreberi*	L	MeOH 70%	96
92	*N. lotus*	AeP	DCM	74
115	*N. lotus*	AeP	Hexane	96
116	*N. lotus*	AeP	DCM	81
69	*V. madiensis*	R	Hexane	79
20	*C. febrifuga*	L	Hexane	85
28	*V. madiensis*	L	Hexane	96
64	*M. charantia*	AeP	DCM	72
35	*B. owariensis*	L	Hexane	96

^1^ measured at 20 µg/mL, mean of three independent replicates.

**Table 3:** Antiprotozoal activities of the 15 selected active extracts, ranked by decreasing activity against *T. b. rhodesiense*. IC_50_ value and the selectivity index (SI), defined. Antitrypanosomal data and SI index represent the mean of three independent determinations and antiplasmodial data of two independent values. The IC_50_ values are in µg/mL. *L. donovani*: axenic amastigote. n.d: not determined. Ri = rhizomes; Rb = Root barks; AeP = aerial parts; L = leaves; Wp = leaves and stems; R = roots.

Extract ID	Plant ^plant part^	*T. b. rhodesiense*		*T. cruzi*		*L. donovani*		*P. falciparum*		L6
		IC_50_	SI ^1^	IC_50_	SI	IC_50_	SI	IC_50_	SI	IC_50_
46	*E. abyssinica ^Ri^*	1.8	4.5	14.0	0.6	29.9	0.3	6.5	1.2	6.3
47	*E. abyssinica ^Ri^*	4.1	4.0	16.1	1.0	43.4	0.4	12.7	1.3	16.3
91	*N. lotus ^AeP^*	4.8	5.8	36.8	0.8	44.2	0.6	10.3	2.7	32.9
54	*E. abyssinica ^Rb^*	5.1	3.6	26.4	0.7	45.8	0.4	10.4	1.8	16.0
109	*B. schreberi ^L^*	5.9	2.9	26.7	0.6	53.0	0.3	3.5	4.9	33.8
110	*B. schreberi ^L^*	7.1	4.3	61.5	0.5	48.1	0.6	8.1	3.8	33.8
111	*B. schreberi ^L^*	7.9	4.0	65.9	0.5	42.4	0.7	7.5	4.2	36.0
92	*N. lotus ^L^*	9.8	3.8	56.7	0.7	14.5	2.5	6.3	5.9	42.4
115	*N. lotus ^L^*	11.9	2.5	45.5	0.6	20.1	1.5	14.7	2.0	34.5
116	*N. lotus ^L^*	12.2	3.6	56.3	0.8	17.7	2.5	7.9	5.5	49.7
69	*V. madiensis ^R^*	12.8	2.2	53.0	0.5	11.7	2.4	20.7	1.4	41.9
20	*C. febrifuga ^L^*	13.1	3.5	64.1	0.7	46.9	1.0	21.2	2.2	47.0
28	*V. madiensis ^L^*	13.6	1.7	42.2	0.6	23.2	1.0	23.9	1.0	22.8
64	*M. charantia ^Wp^*	30.5	1.1	48.1	0.7	25.5	1.3	8.7	3.9	26.0
35	*B. owariensis ^L^*	40.2	1.2	55.9	0.9	62.1	0.8	>50	n.d	48.2

^1^ Selectivity Index, defined as the IC_50_ towards mammalian L6 cells divided by the IC_50_ towards the parasite.

**Table 4:** Antiprotozoal activity of the active compounds identified from *N. lotus* (extract ID 116, Table 2) and *B. schreberi* (extract ID 110, Table 2). IC_50_ values are in µg/mL and represent the mean of two independent experiments. *L. donovani*: axenic amastigote. n.d.: not determined.

	*T. b. rhodesiense*		*T. cruzi*		*L. donovani*		*P. falciparum*		L6
	IC_50_	SI	IC_50_	SI	IC_50_	SI	IC_50_	SI	IC_50_
Gallic acid (**1**)	0.5	34	66	0.2	56	0.3	>10	n.d.	16
Methyl gallate (**2**)	1.1	15	16	1.0	8.5	1.9	2.1	7.8	16
Ethyl gallate (**4**)	0.6	25	16	0.9	6.8	2.2	3.0	4.9	15
Pentagalloyl-β-glucopyranoside (**5**)	20.0	1.0	44	0.5	15	1.4	6.7	3.1	21
Gossypetin-7-*O*-β-glucopyranoside (**6**)	5.5	1.6	12	0.8	53	0.2	n.d.	n.d.	8.9
Hypolaetin-7-*O*-glucoside (**7**)	5.7	3.2	49	0.4	52	0.4	n.d.	n.d.	19
Resorcinol-alkyl (**8**)	5.3	2.5	9.1	1.4	2.5	5.2	n.d.	n.d.	13

**Table 5:** Quantification of the main constituents in the decoctions of *B. schreberi* and of *N. lotus*. The values are in relation to the dried raw plant material (mg/g) and to the dry extract (mg/g). n.d: not determined.

Active Component	*B. schreberi* Decoction		*N. lotus* Decoction	
	Raw Material	Extract	Raw Material	Extract
Gallic acid (**1**)	8.8	50	5.6	22
Methyl gallate (**2**)	0.007	0.04	0.005	0.022
Ethyl gallate (**4**)	n.d.	<19 ppm	n.d.	<19 ppm
Pentagalloyl-β-glucopyranoside (**5**)	0.39	2.3	0.09	0.36

## Data Availability

The original contributions presented in the study are included in the article/Supplementary Materials, further inquiries can be directed to the corresponding author/s.

## Supplementary Materials

The following are available online at https://www.mdpi.com/article/10.3390/molecules29071611/s1, Table S1: Overview of the all the plants extracts, their preparation and antitrypanosomal activity, Figure S1: ELSD chromatograms for the ethanolic extract of *B. schreberi* before and after VLC enrichment, Figure S2: Growth inhibition activity (%) against T. b. rhodesiense of *B. schreberi* leave extracts at 20 and 10 µg/mL, Figure S3: Separation of fraction F6 (chromatogram A), F10 (chromatogram B) and F12 (chromatogram C) of VLC methanolic extract of the leaves of *B. schreberi*, Figure S4: 1H NMR data and spectrum of compound **1** in CD3OD at 600 MHz, Figure S5: NMR data and spectra of compound **2** in CD3OD at 600 MHz, Figure S6: 1H NMR data and spectrum of compound **3** in CD3OD at 600 MHz, Figure S7: NMR data and spectra of compound **4** in CD3OD at 600 MHz, Figure S8: NMR data and spectra of compound **5** in CD3OD at 600 MHz, Figure S9: NMR data and spectra of compound **6** in DMSO-d6 at 600 MHz, Figure S10: NMR data and spectra of compound **7** in CD3OD at 600 MHz, Figure S11: NMR data and spectra of compound **8** in CD3OD at 600 MHz, Figure S12: UHPLC-HRMS chromatogram showing the presence of compounds **1** and **7** in the decoction of *N. lotus* and correspondence of their MS/MS spectra, Figure S13: UHPLC-HRMS chromatogram showing the presence of compounds **1**, **2**, **5**, **6** and **7** in the decoction of *B. schreberi*, [32,34,36,37,52,84,85].
