# Supplementary material for: Antiprotozoal Activity of Plants Used in the Management of Sleeping Sickness in Angola and Bioactivity-Guided Fractionation of Brasenia schreberi J.F.Gmel and Nymphaea lotus L. Active against T. b. rhodesiense
Source: Molecules. 2024 Apr 3;29(7):1611. doi: 10.3390/molecules29071611 (PMC11013945; doi:10.3390/molecules29071611)
Supplement: Supplementary file 1 [file molecules-29-01611-s001.zip › molecules-2849972-supplementary.pdf]

Supplementary Materials

Antiprotozoal activity of plants used in the management of sleeping sickness in Angola and bioactivity-guided fractionation of *Brasenia schreberi* J.F.Gmel and *Nymphaea lotus* L. active against *T. b. rhodesiense*

Nina Vahekeni [1,2*], Théo Brillatz [3], Marjan Rahmaty [3], Monical Cal [1,2], Sonja Keller-Maerki [1,2], Romina Rocchetti [1,2], Marcel Kaiser [1,2], Sibylle Sax [1,2], Kevin Mattli [4], Evelyn Wolfram [4], Laurence Marcourt [3], Emerson F. Queiroz [3], Jean-Luc Wolfender [3] and Pascal Mäser [1,2]

***Corresponding author:**
vahe@zhaw.ch; Tel.: +41 078 908 83 86

Summary

Table S1: Overview of all the plants extracts, their preparation and antitrypanosomal activity.

Figure S1: ELSD chromatograms for the ethanolic extract of *B. schreberi* before and after VLC enrichment.

Figure S2: Growth inhibition activity (%) against *T. b. rhodesiense* of *B. schreberi* leave extracts at 20 and 10 μg/mL.

Figure S3: Separation of fraction F6 (chromatogram A), F10 (chromatogram B) and F12 (chromatogram C) of VLC methanolic extract of the leaves of *B. schreberi*.

Figure S4: ^{1}H NMR data and spectrum of compound **1** in CD_3OD at 600 MHz.

Figure S5: NMR data and spectra of compound **2** in CD_3OD at 600 MHz.

Figure S6: ^{1}H NMR data and spectrum of compound **3** in CD_3OD at 600 MHz.

Figure S7: NMR data and spectra of compound **4** in CD_3OD at 600 MHz.

Figure S8: NMR data and spectra of compound **5** in CD_3OD at 600 MHz.

Figure S9: NMR data and spectra of compound **6** in DMSO-d_6 at 600 MHz.

Figure S10: NMR data and spectra of compound **7** in CD_3OD at 600 MHz.

Figure S11: NMR data and spectra of compound **8** in CD_3OD at 600 MHz.

Figure S12: UHPLC-HRMS chromatogram showing the presence of compounds **1** and **7** in the decoction of *N. lotus* and correspondence of their MS/MS spectra.

Figure S13: UHPLC-HRMS chromatogram showing the presence of compounds **1**, **2**, **5**, **6** and **7** in the decoction of *B. schreberi*.

References

Table S1: Overview of all the plants extracts, their preparation and antitrypanosomal activity. Growth inhibition activity is categorized in five groups: **strong activity** in green (GI of 91% - 100%), **marked activity** in brown (71% - 90% GI), **moderate activity** in dark blue (51% - 70% GI), **weak activity** in light blue (31% - 50% GI) and **inactive** in greyish blue (GI <30%).

Extract ID	Plant name	Plant part	Sample labeling	Solvent	Raw mass of dried plant material (g)	Drug/ solvent ratio	Weight of dried extract (g)	Yield of extract (%)	Growth inhibition (%) at 20 µg/ml
1	*N. lotus & B. schreberi (mixture)*	leaves	11	AqDec	10.18	1:20	1.703	16.7	97
2			12	EtOH80%	15.20	1:10	2.27	14.9	95
3			13	MeOH70%	14.8	1:8	1.95	13.2	96
4	*C. febrifuga*	trunk bark	46	AqDec	30.09	1:20	3.794	12.6	17
5			47	EtOH80%	15.1	1:10	1.88	12.5	26
6			4a	Hexane	100.04	1:3	0.075	0.1	38
7			4b	DCM		1:3	0.088	0.1	31
8			4c	MeOH		1:3	9.139	9.1	26
9			4d	H20		1:3	1.479	1.5	17
10	*C. febrifuga*	root	58	AqDec	15.09	1:20	1.673	11.1	16
11			59	AqMac	15.1	1:20	1	6.6	18
12			510	EtOH80%	13.7	1:10	0.92	6.7	22
13			5a	Hexane	50.52	1:3	0.051	0.1	37
14			5b	DCM		1:3	0.080	0.2	37
15			5c	MeOH		1:3	4.576	9.1	31
16			5d	H20		1:3	0.589	1.2	12
17	*C. febrifuga*	leaves	611	AqDec	15.08	1:20	3.749	24.9	22
18			612	EtOH80%	15.114	1:10	2.4	15.9	28
19			613	MeOH80%	15.1	1:10	2.95	19.5	26
20			6a	Hexane	100	1:3	1.247	1.2	85
21			6b	DCM		1:3	0.571	0.6	56
22			6c	MeOH		1:3	11.191	11.2	21
23			6d	H20		1:3	2.656	2.7	12
24	*V. madiensis*	leaves	714	AqDec	15.0928	1:20	2.442	16.2	28
25			715	EtOH80%	15.2	1:10	1.82	12.0	33
26			716	MetT	15	1:20	2.656	17.7	29
27			717	MeOH	10.5	1:4.2	1.2	11.4	35
28			7a	Hexane	83.94	1:3	0.688	0.8	96
29			7b	DCM		1:3	0.746	0.9	62
30			7c	MeOH		1:3	12.191	14.5	22
31			7d	H20		1:3	2.667	3.2	17
32	*B. owariensis*	leaves	818	AqDec	19.995	1:20	5.864	29.3	10
33			819	EtOH80%	20.3	1:10	2.681	13.2	23
34			820	MetT	15.1	1:20	3.509	23.2	11
35			8a	Hexane	100.13	1:3	1.007	1.0	96
36			8b	DCM		1:3	1.621	1.6	74
37			8c	MeOH		1:3	5.123	5.1	34
38			8d	H20		1:3	8.353	8.3	22
39	*E. abyssinica*	trunk bark	1021	AqDec	30.259	1:20	5.539	18.3	61
40			1022	EtOH80%	50.19	1:10	3.4	6.8	70
41			10a	Hexane	100.18	1:8	0.404	0.4	75
42			10b	DCM		1:8	0.560	0.6	52
43			10c	MeOH		1:8	13.405	13.4	57
44			10d	H20		1:8	0.579	0.6	13

45	*E. abyssinica*	root inside	11a23	AqDec	15.01	1:20	1.431	9.5	85
46			11a24	AqMac	15	1:20	2.1	14.0	103
47			11a25	EtOH80%	20.123	1:10	2.133	10.6	101
48			11a1	Hexane	100.08	1:8	2.608	2.6	58
49			11a2	DCM		1:8	0.167	0.2	33
50			11a3	MeOH		1:8	12.731	12.7	35
51			11a4	H20		1:8	1.828	1.8	0
52	*E. abyssinica*	root bark	11b26	AqDec	20.329	1:20	4.349	21.4	88
53			11b27	AqMac	10.2	1:20	1.6	15.7	89
54			11b28	EtOH80%	15.121	1:10	2.249	14.9	98
55		root (all)	11b1	Hexane	100.08	1:4	0.395	0.4	20
56			11b2	DCM		1:4	0.317	0.3	53
57			11b3	MeOH		1:4	17.425	17.4	93
58			11b4	H20		1:4	0.574	0.6	29
59	*M. charantia*	whole plant	1229	AqDec	15.015	1:20	2.822	18.8	16
60			1230	AqMac	5	1:20	0.97	19.4	16
61			1231	EtOH80%	24.2	1:10	2.514	10.4	27
62			1232	MeOH80%	9.9	1:10	1.27	12.8	22
63			12a	Hexane	40.81	1:3	0.152	0.4	58
64			12b	DCM		1:3	0.588	1.4	72
65			12c	MeOH		1:3	1.52	3.7	30
66			$12d_1$	H20		1:3	3.949	9.7	22
67	*V. madiensis*	root	1333	AqDec	30.4	1:20	4.858	16.0	26
68			1334	EtOH80%	15.1	1:10	1.423	9.4	24
69			13a	Hexane	100.13	1:3	0.102	0.1	79
70			13b	DCM		1:3	0.069	0.1	50
71			13c	MeOH		1:3	7.818	7.8	7
72			13d	H20		1:3	3.174	3.2	0
73	*S. latifolius*	root	1435	AqDec	15.136	1:20	2.357	15.6	18
74			1436	AqMac	5	1:20	0.47	9.4	16
75			1437	EtOH80%	29.8	1:10	1.922	6.4	18
76			1438	MeOH	10.1	1:10	0.64	6.3	32
77			14a	Hexane	52.08	1:3	1.911	3.7	18
78			14b	DCM		1:3	0.264	0.5	60
79			14c	MeOH		1:3	2.866	5.5	36
80			$14d_1$	H20		1:3	1.285	2.5	24
81	*P. schweinfurthii*	leaves	1539	AqDec	15.7	1:20	2.178	13.9	10
82			1540	aqMac	5.3	1:20	0.91	17.2	8
83			1541*	EtOH80%	30	1:10	2.208	7.4	15
84			15a	Hexane	50.06	1:5	0.234	0.5	41
85			15b	DCM		1:5	0.387	0.8	17
86			15c	MeOH		1:5	1.085	2.2	13
87			$15d_1$	H20		1:5	5.373	10.7	6
88	*N. lotus*	whole plant	1642	AqDec	14.7	1:20	3.8	25.9	35
89			1643	MeOH70%	15.1	1:8	1.73	11.5	61
90			1644**	EtOH80%	15	1:10	2.196	14.6	58
91			16a	Hexane	21	1:3	0.616	2.9	98
92			16b	DCM		1:3	0.282	1.3	74
93			16c	MeOH		1:3	0.716	3.4	13
94			$16d_1$	H20		1:3	2.018	9.6	22
95	*B.schreberi*	whole plant	1745	AqDec	10.1	1:20	1.19	11.8	41
96			1746	EtOH80%	20.394	1:10	2.163	10.6	92
97			1747	MeOH70%	15	1:8	1.02	6.8	8
98			17a	Hexane	50.08	1:2	0.179	0.4	94
99			17b	DCM		1:2	0.096	0.2	79
100			17c	MeOH		1:2	1.954	3.9	76
101			$17d_1$	H20		1:2	1.585	3.2	16

102	B.schreberi	stem (sub aquatic part)	1848	AqDec	10.51	1:20	1.232	11.7	20
103			1849	EtOH80%	20.08	1:10	1.804	9.0	31
104			1850	MeOH70%	15.1	1:8	0.79	5.2	21
105			18a	Hexane	19.72	1:5	0.181	0.9	75
106			18b	DCM		1:5	0.079	0.4	70
107			18c	MeOH		1:5	1.645	8.3	23
108			18d$_1$	H20		1:5	1.225	6.2	27
109	B. schreberi	leaves	1951	AqDec	20	1:20	3.5	17.5	99
110			1952	EtOH80%	25.2	1:10	4.164	16.5	96
111			1953	MeOH70%	15.1	1:8	1.83	12.1	96
112	N. lotus (bungo batch)	whole plant	2054	AqDec	15.1	1:20	3.9	25.8	32
113			2055	EtOH80%	19.806	1:10	2.31	11.7	46
114			2056	MeOH70%	14.96	1:8	2.07	13.8	51
115			20a	Hexane	20.12	1:5	0.404	2.0	96
116			20b	DCM		1:5	0.199	1.0	81
117			20c	MeOH		1:5	1.719	8.5	23
118			20d$_1$	H20		1:5	1.544	7.7	9
119	N. lotus (Damba batch)	whole plant	2157	AqDec	15.1	1:20	3.5	23.2	7
120			2158*	EtOH80%	19.806	1:10	2.31	11.7	23
121			2159	MeOH70%	13.02	1:8	1.29	9.9	18
122	MIX	whole plant	Mix60	AqMac	5.08	1:20	2.33	15.4	6
		root			5.12				
		leaves			4.93				

Figure S1: ELSD chromatograms for the ethanolic extract of *B. schreberi* before and after VLC enrichment.

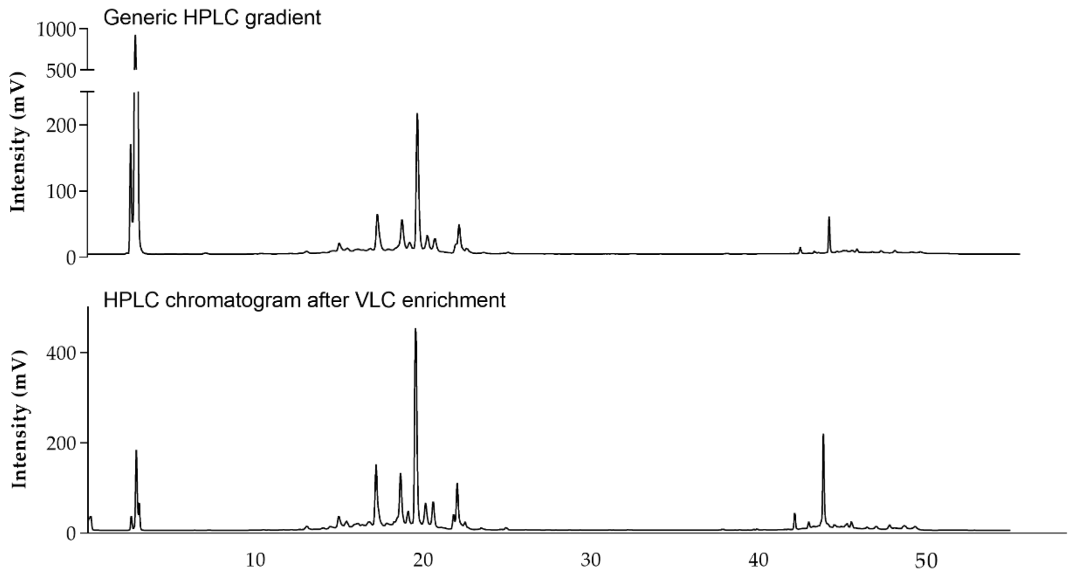

Figure S2: Growth inhibition activity (%) against *T. b. rhodesiense* of *B. schreberi* leave extracts at 20 and 10 µg/mL. The inhibitory activity of the crude ethanol 80% extract is compared to its enriched VLC fractions. VLC methanolic fraction (VLC_MeOH) displayed most promising antitrypanosomal activity with a GI (%) of 84.6 at 10 µg/mL and 95.1% at 20 µg/mL. Legends for *B. schreberi* leave extracts: ethanol 80% crude extract (BS_EE80), VLC aqueous fraction ((BS_EE80_VLC_H2O), VLC methanolic fraction ((BS_EE80_VLC_MeOH), VLC ethyl acetate fraction ((BS_EE80_VLC_EtoAc).

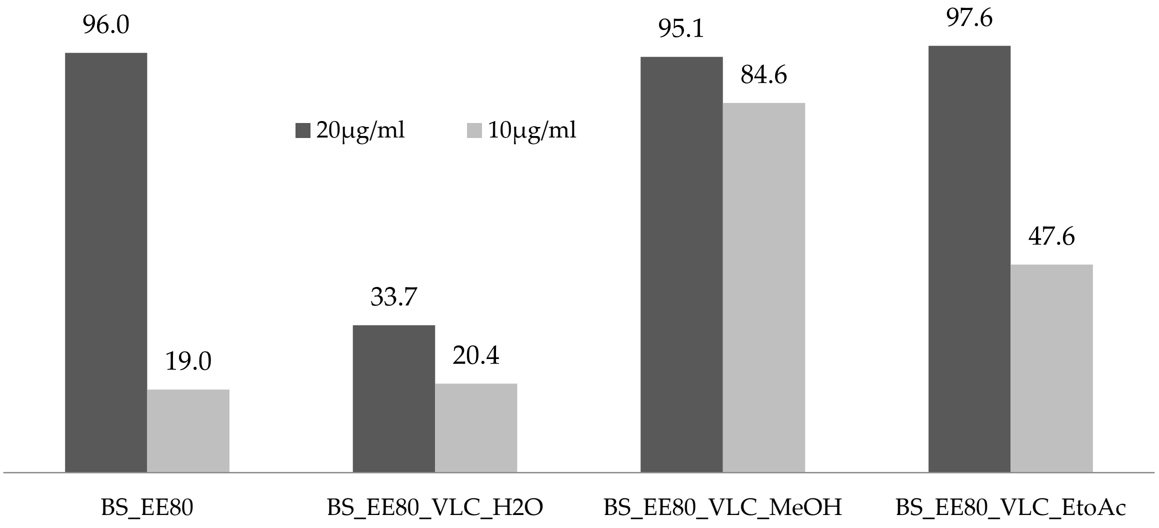

Figure S3: Separation of fraction F6 (chromatogram A), F10 (chromatogram B) and F12 (chromatogram C) of VLC methanolic extract of the leaves of *B. schreberi*. F6 yielded methyl gallate (**2**) and 2,3,4,6 tetragalloyl-glucopyranoside (**3**). UV detector at 280 nm. Subfractionation of F10 yielded ethyl gallate (**4**) and 1,2,3,4,6 pentagalloyl-β-glucopyranoside (**5**); UV detector at 280 nm. F12 yielded hypolaetin-7-*O*-glucoside (**7**); UV detector at 254 nm. P19_EE80_VLC_MeOH = VLC ethanolic extract of the leaves of *B. schreberi*.

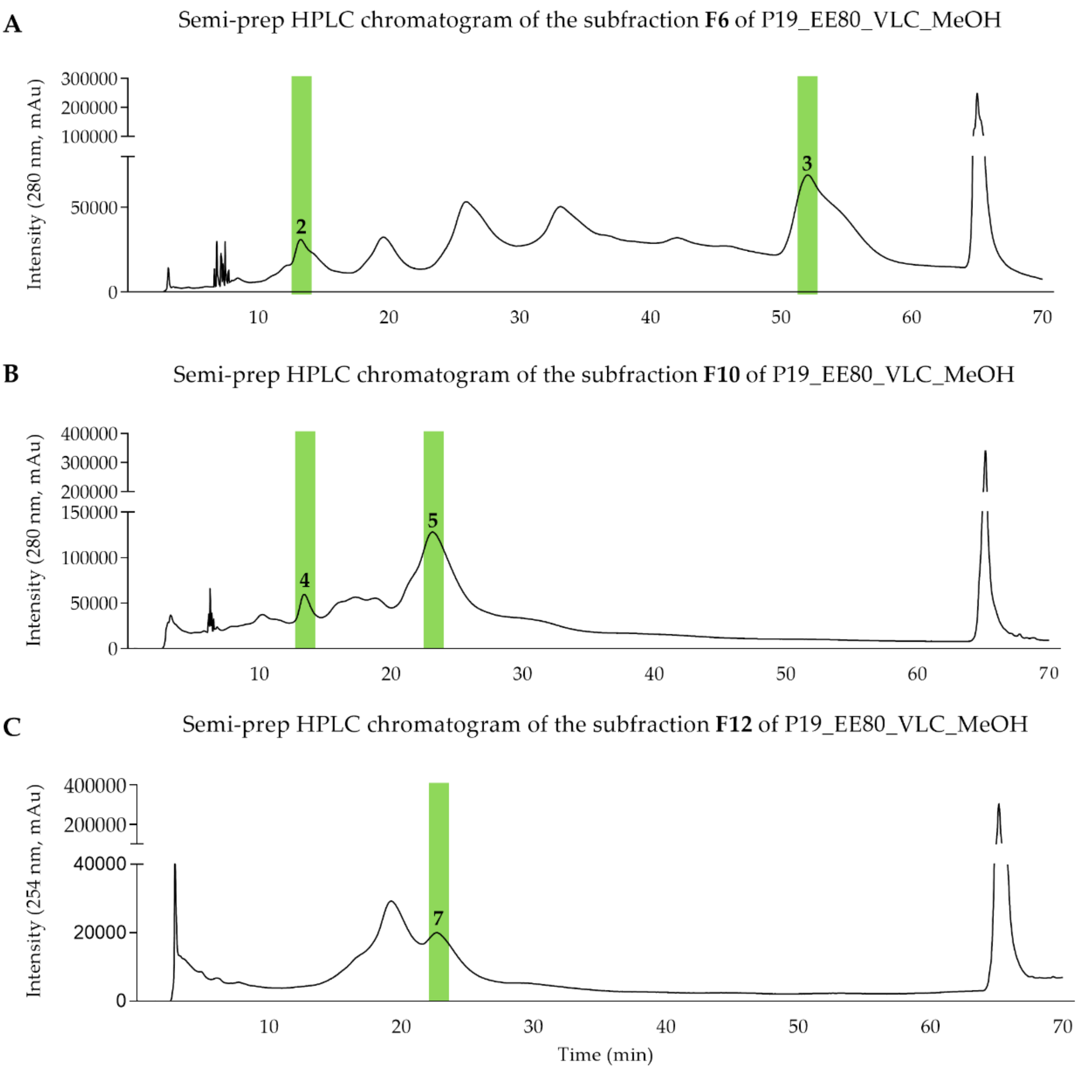

NMR data

Figure S4: ^{1}H NMR data and spectrum of compound **1** in CD_3OD at 600 MHz.

Gallic acid (**1**) (Kamatham *et al.*, 2015): ^{1}H NMR (CD_3OD, 600 MHz) δ 7.04 (2H, s, H-2, H-6). HRESIMS *m/z* 169.0136 $[M-H]^-$ (calcd for $C_7H_6O_5^-$, 169.01370, Δ = -3.7 ppm).

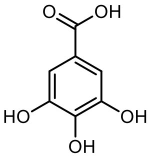

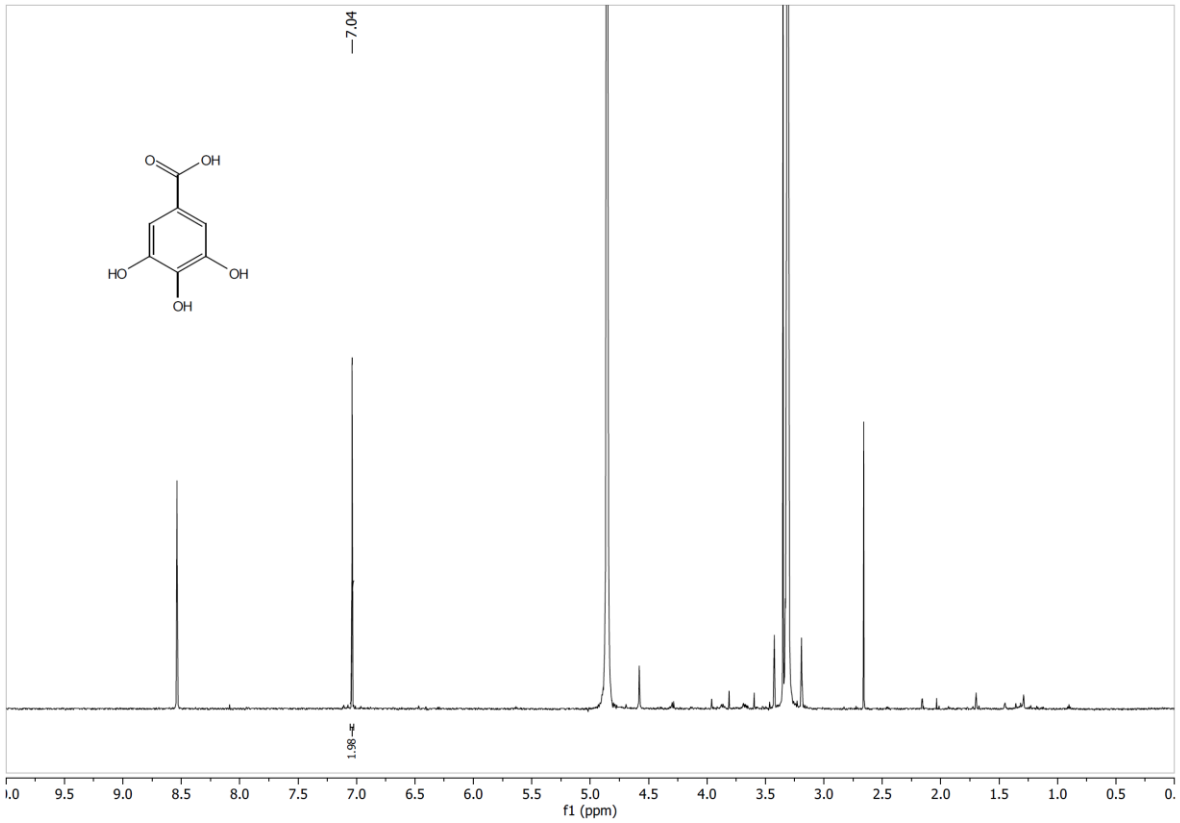

Figure S5: NMR data and spectra of compound **2** in CD_3OD at 600 MHz.

Methyl gallate (**2**) (Kamatham *et al.*, 2015): ^{1}H NMR (CD_3OD, 600 MHz) δ (3H, s, CH_3-8), 7.04 (2H, s, H-2, H-6); ^{13}C NMR (CD_3OD, 151 MHz) δ 51.9 (CH_3-8), 109.7 (C-2, C-6), 139.6 (C-4), 163.2 (C-3, C-5), 168.8 (C-7). HRESIMS m/z 183.0302 [M-H]- (calcd for $C_8H_7O_5^-$, 183.02935, Δ = 1.8 ppm).

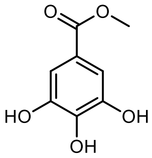

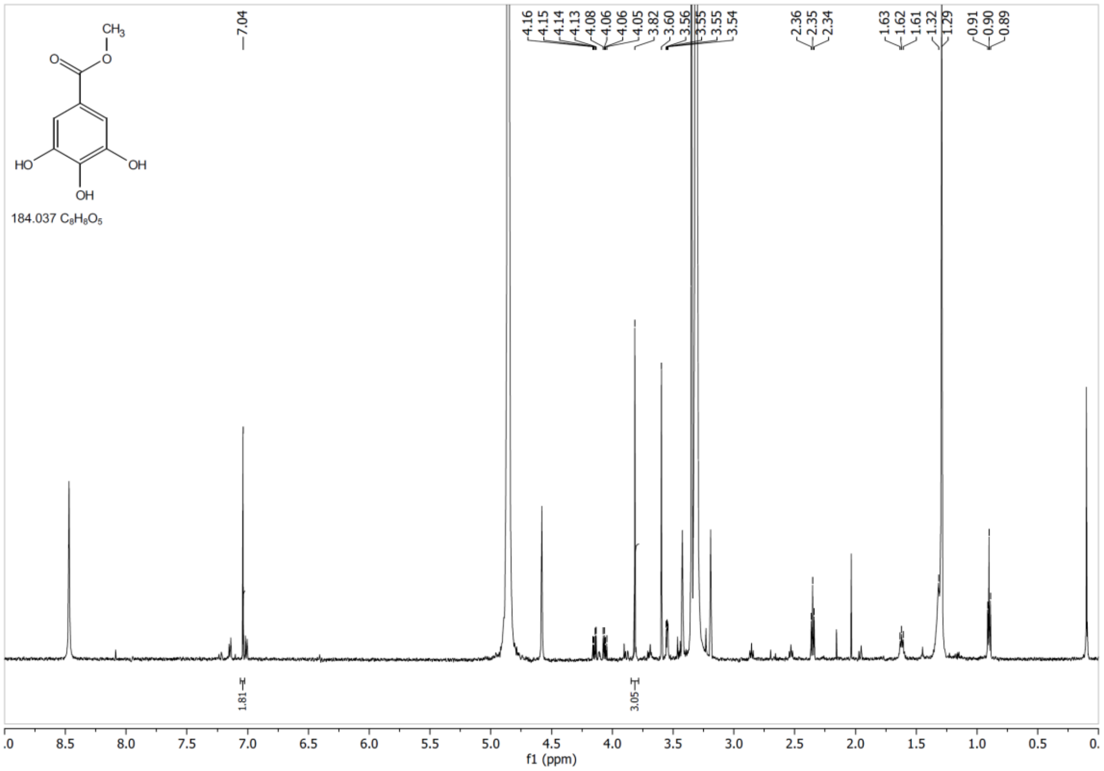

^{1}H NMR spectrum of compound **2** in CD_3OD at 600 MHz.

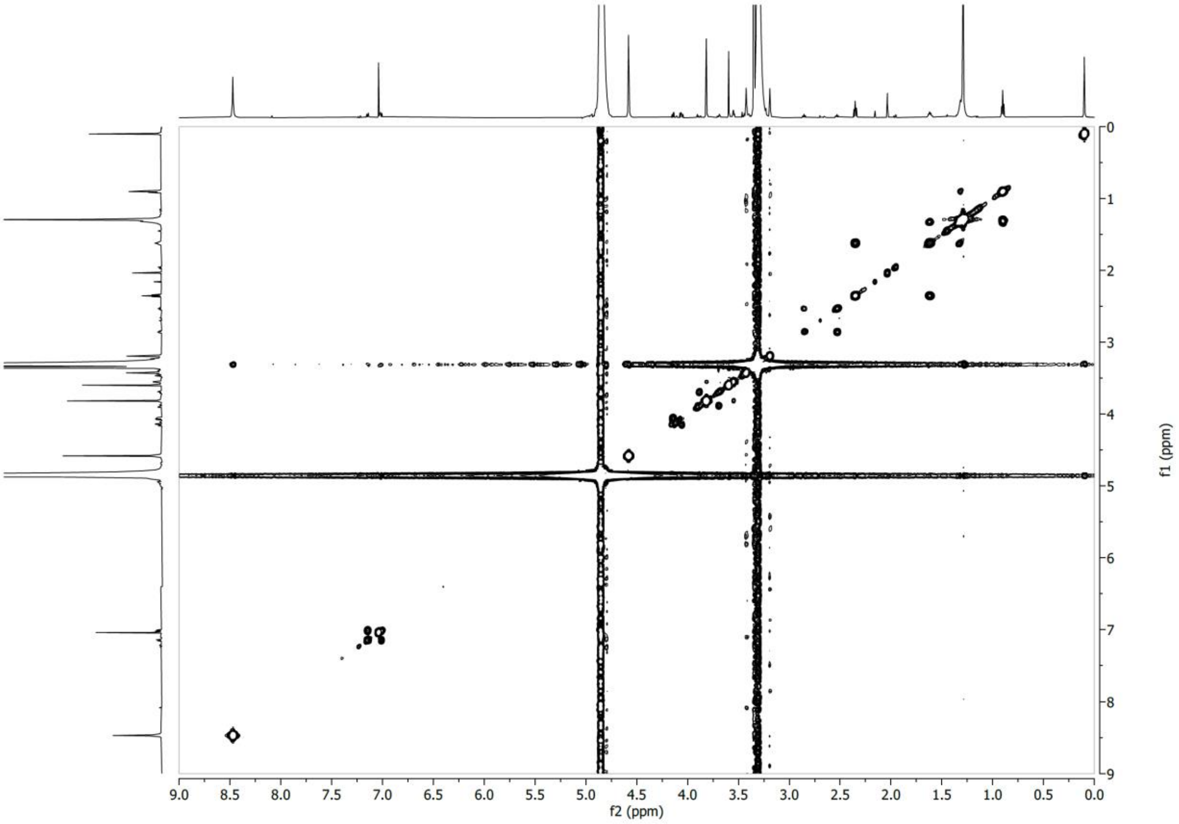

COSY NMR spectrum of compound **2** in CD_3OD.

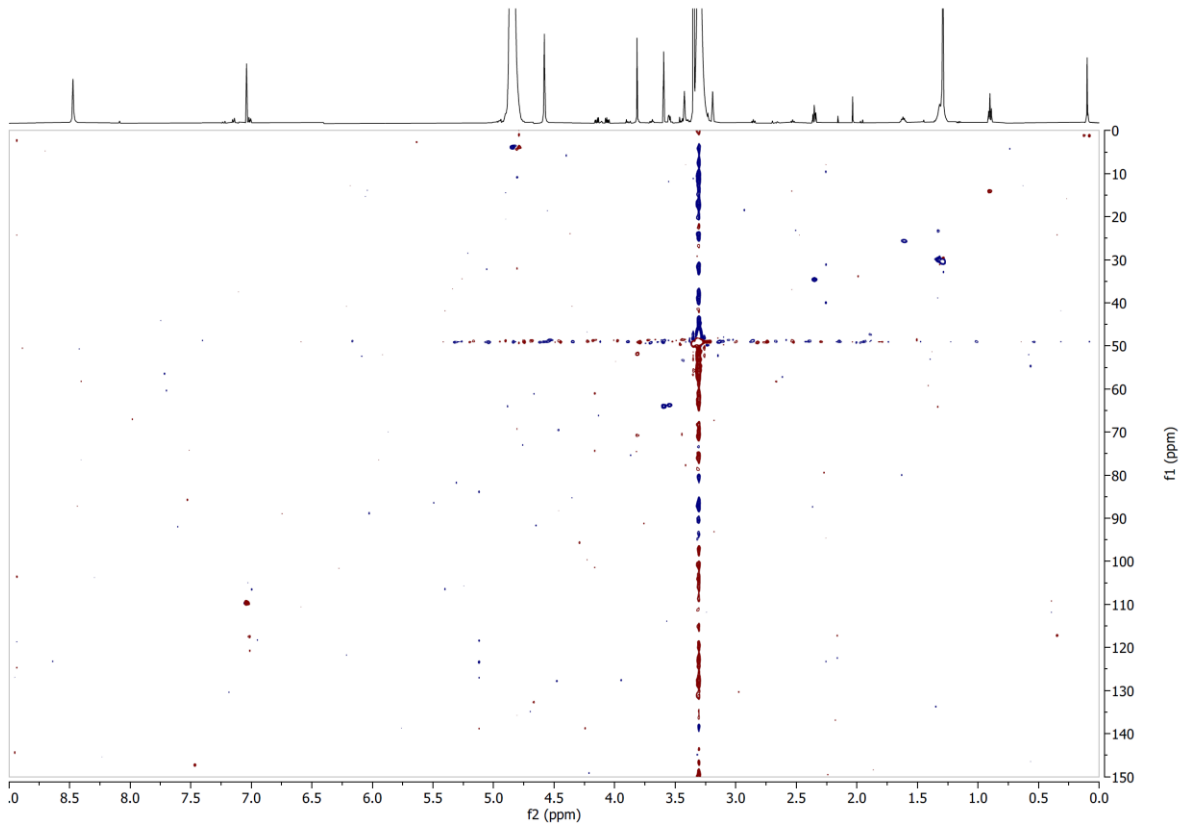

Edited-HSQC NMR spectrum of compound **2** in CD_3OD.

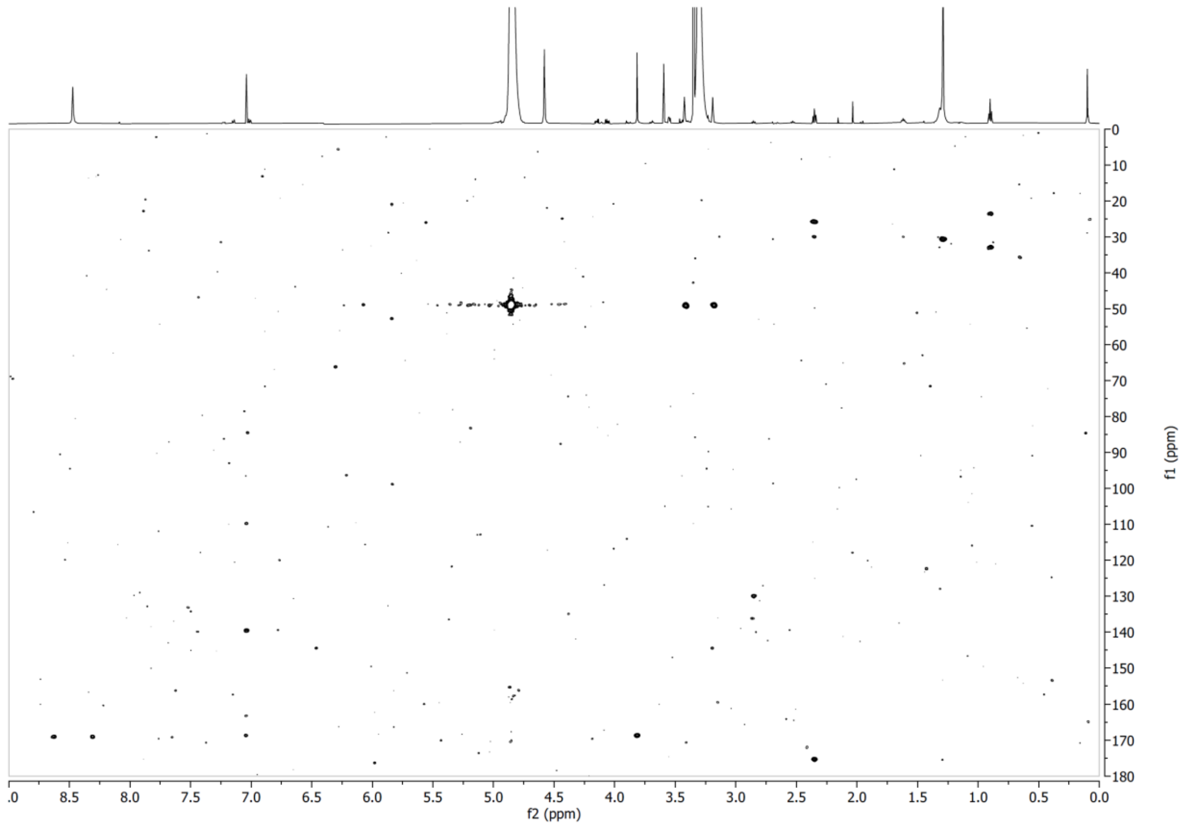

HMBC NMR spectrum of compound **2** in CD_3OD.

Figure S6: ^{1}H NMR data and spectrum of compound **3** in CD_3OD at 600 MHz.

2,3,4,6 tetragalloyl-glucopyranoside (**3**) (Tanaka *et al.*, 1983): Mixture of α and β-glucopyranoside (1.0/0.7). ^{1}H NMR (CD_3OD, 600 MHz) δ 4.32 (2H, m, α-H-6b, β-H-6b), 4.44 (1H, d, *J* = 11.1 Hz, α-H-6a), 4.50 (1H, dd, *J* = 12.3, 2.4 Hz, β-H-6a), 5.08 (1H, d, *J* = 7.9 Hz, β-H-1), 5.15 (1H, dd, *J* = 10.0, 3.5 Hz, α-H-2), 5.24 (1H, dd, *J* = 9.8, 7.9 Hz, β-H-2), 5.52 (3H, m, α-H-1, α-H-4, β-H-4), 5.74 (1H, t, *J* = 9.8 Hz, β-H-3), 6.01 (1H, t, *J* = 10.0 Hz, α-H-3), H-2'/H-6' of galloyl from α-form: 6.91 (2H, s), 6.98 (2H, s), 7.02 (2H, s), 7.12 (2H, s), H-2'/H-6' of galloyl from β-form: 6.87 (2H, s), 6.96 (2H, s), 7.00 (2H, s), 7.11 (2H, s). HRESIMS *m/z* 787.1049 and 787.1051 [M-H]$^-$ (calcd for $C_{34}H_{27}O_{22}^-$, 787.09940, Δ = 6.3 ppm).

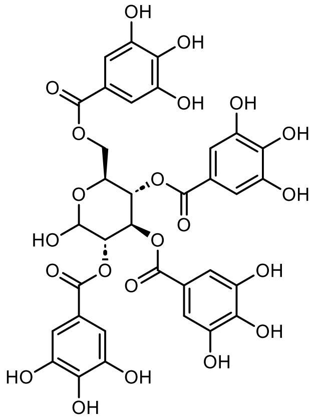

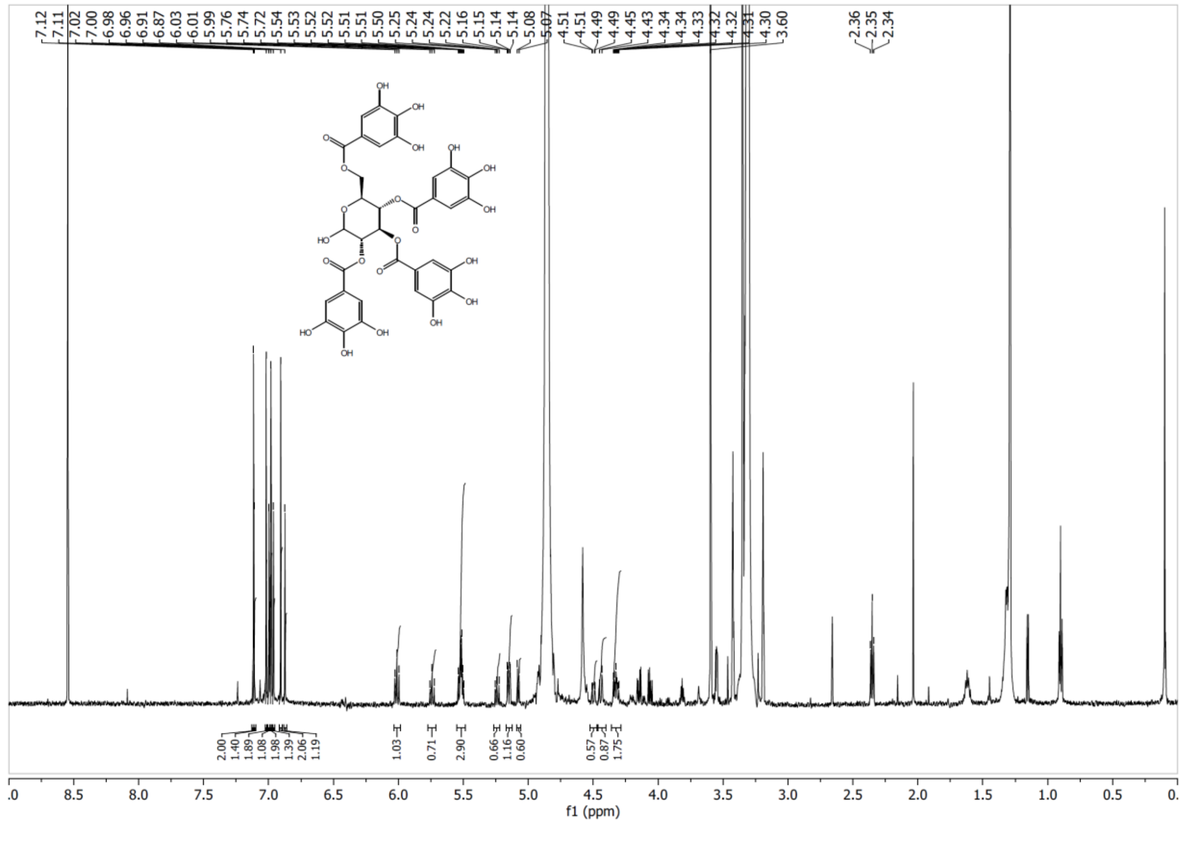

^{1}H NMR spectrum of compound **3** in CD_3OD at 600 MHz.

Figure S7: NMR data and spectra of compound **4** in CD_3OD at 600 MHz.

Ethyl gallate (**4**) (Leela *et al.*, 2013): ^{1}H NMR (CD_3OD, 600 MHz) δ 1.35 (3H, t, *J* = 7.1 Hz, CH_3-9), 4.27 (2H, q, *J* = 7.1 Hz, H-8), 7.04 (2H, s, H-2, H-6); ^{13}C NMR (CD_3OD, 151 MHz) δ 14.2 (CH_3-9), 61.2 (C-8), 109.7 (C-2, C-6), 121.5 (C-1), 139.6 (C-4), 168.2 (C-7). HRESIMS *m/z* 197.0459 $[M-H]^-$ (calcd for $C_9H_9O_5^-$, 197.04500, Δ = 1.9 ppm).

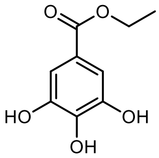

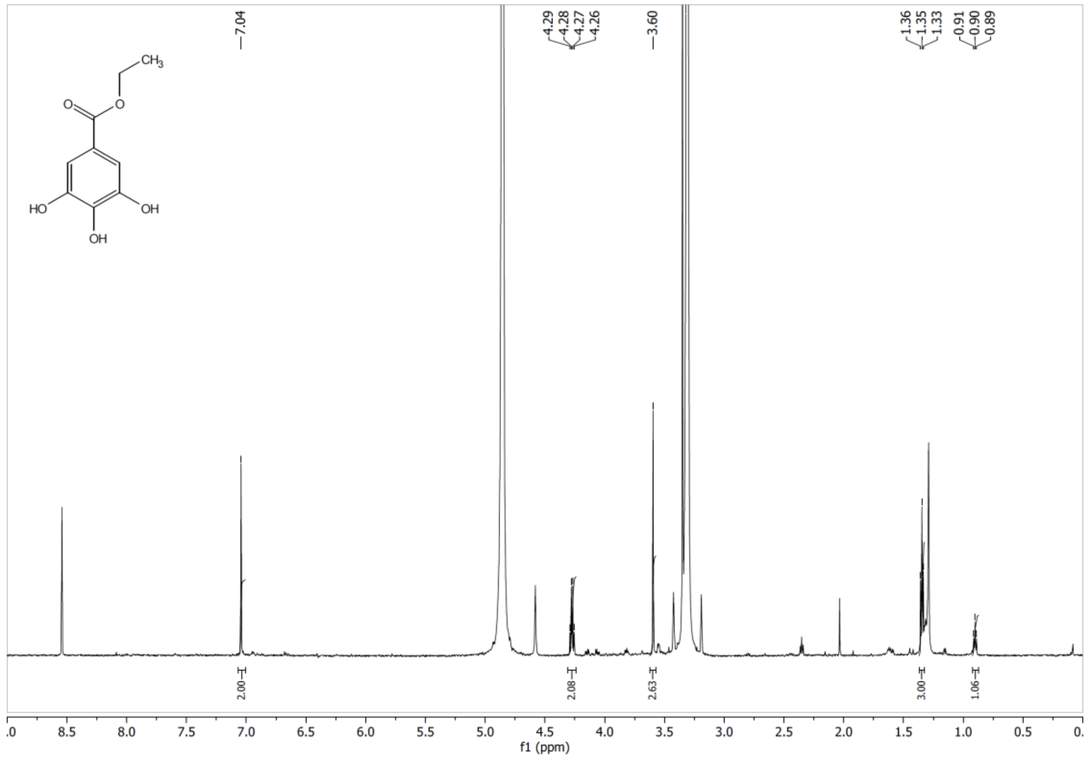

^{1}H NMR spectrum of compound **4** in CD_3OD at 600 MHz.

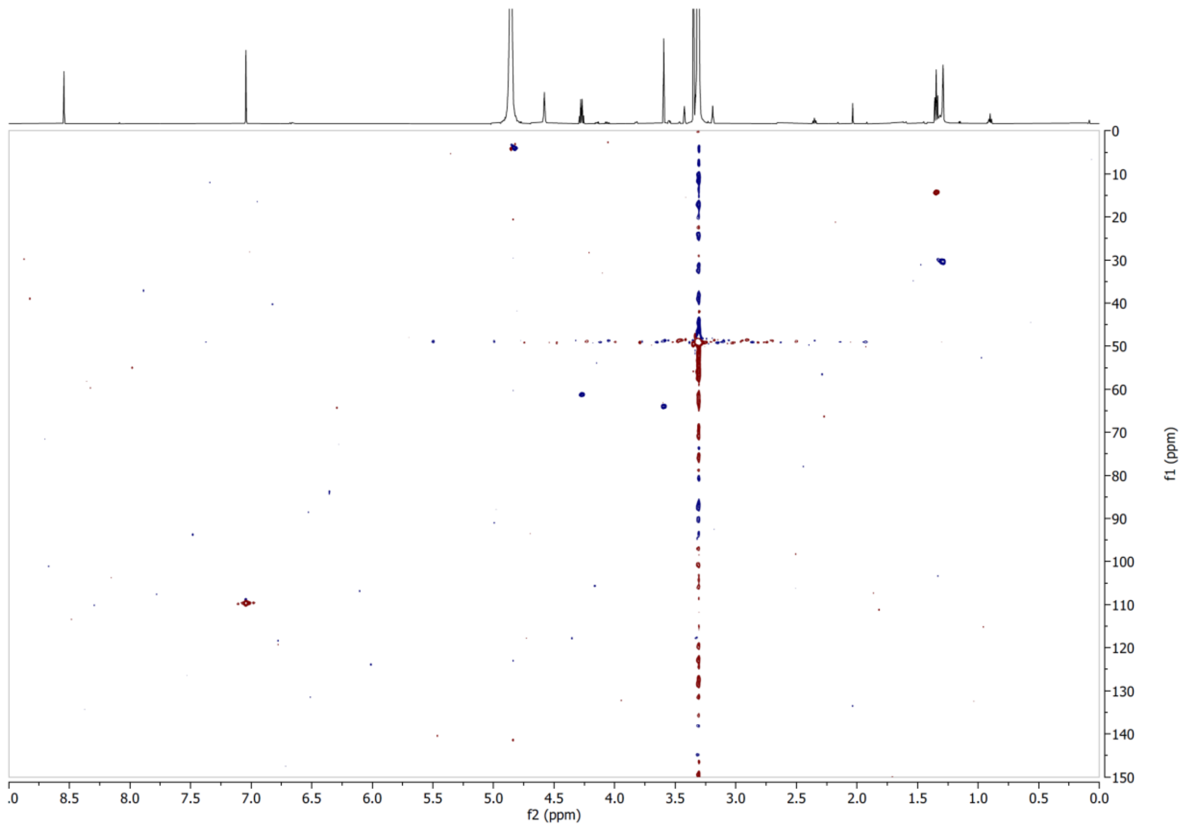

Edited-HSQC NMR spectrum of compound **4** in CD_3OD.

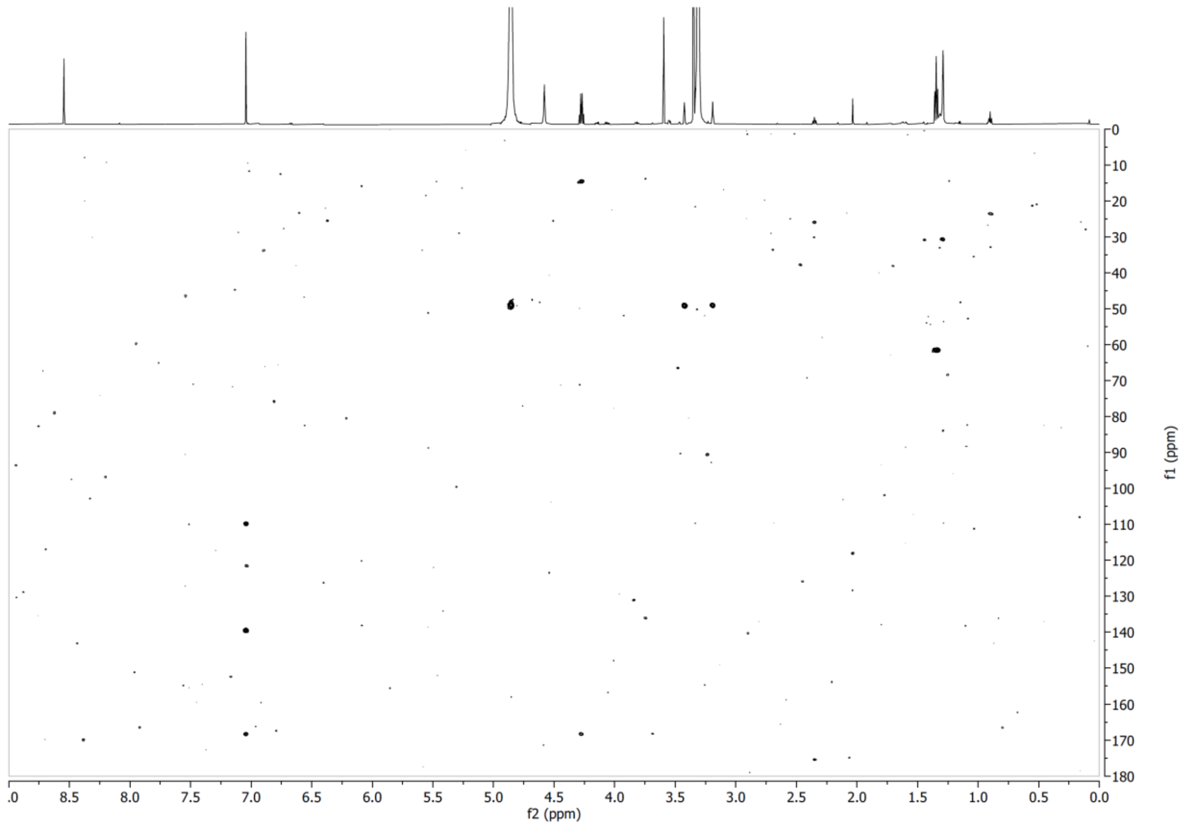

HMBC NMR spectrum of compound **4** in CD_3OD.

Figure S8: NMR data and spectra of compound **5** in CD_3OD at 600 MHz.

1,2,3,4,6 pentagalloyl-β-glucopyranoside (**5**) (Cui *et al.*, 2002): ^{1}H NMR (CD_3OD, 600 MHz) δ 4.38 (1H, dd, *J* = 12.1, 4.4 Hz, H-6b), 4.41 (1H, m, H-5), 4.51 (1H, dd, *J* = 12.1, 1.6 Hz, H-6a), 5.59 (1H, dd, *J* = 9.7, 8.3 Hz, H-2), 5.62 (1H, t, *J* = 9.7 Hz, H-4), 5.90 (1H, t, *J* = 9.7 Hz, H-3), 6.24 (1H, d, *J* = 8.3 Hz, H-1), 6.90 (2H, s, H-2'-3, H-6'-3), 6.95 (2H, s, H-2'-2, H-6'-2), 6.98 (2H, s, H-2'-4, H-6'-4), 7.05 (2H, s, H-2'-1, H-6'-1), 7.11 (2H, s, H-2'-6, H-6'-6); ^{13}C NMR (CD_3OD, 151 MHz) δ 63.1 (C-6), 69.8 (C-4), 72.2 (C-2), 74.1 (C-3), 74.4 (C-5), 93.8 (C-1), 110.3 (C-2'-6, C-6'-6), 110.4 (C-2'-3, C-6'-3), 110.4 (C-2'-2, C-6'-2), 110.5 (C-2'-4, C-6'-4), 110.6 (C-2'-1, C-6'-1), 140.0 (C-4'-6), 140.1 (C-4'-3), 140.3 (C-4'-4), 140.4 (C-4'-2), 140.8 (C-4'-1), 146.3 (C-3'-3, C-5'-3), 146.4 (C-3'-2, C-5'-2), 146.5 (C-3'-4, C-5'-4), 146.5 (C-3'-6, C-5'-6), 146.6 (C-3'-1, C-5'-1), 166.2 (C-7'-1), 166.9 (C-7'-4), 167.0 (C-7'-2), 167.3(C-7'-3), 167.9 (C-7'-6). HRESIMS *m/z* 939.1157 $[M-H]^{-}$ (calcd for $C_{41}H_{31}O_{26}^{\ -}$, 939.11036, Δ = 5.1 ppm), only observed as *m/z* 469.0521 $[M-H]^{2-}$ in the decoction extract of *B. schreberi*.

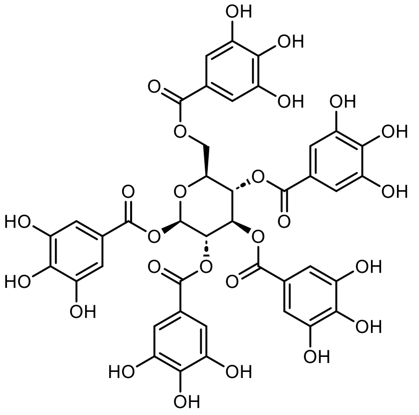

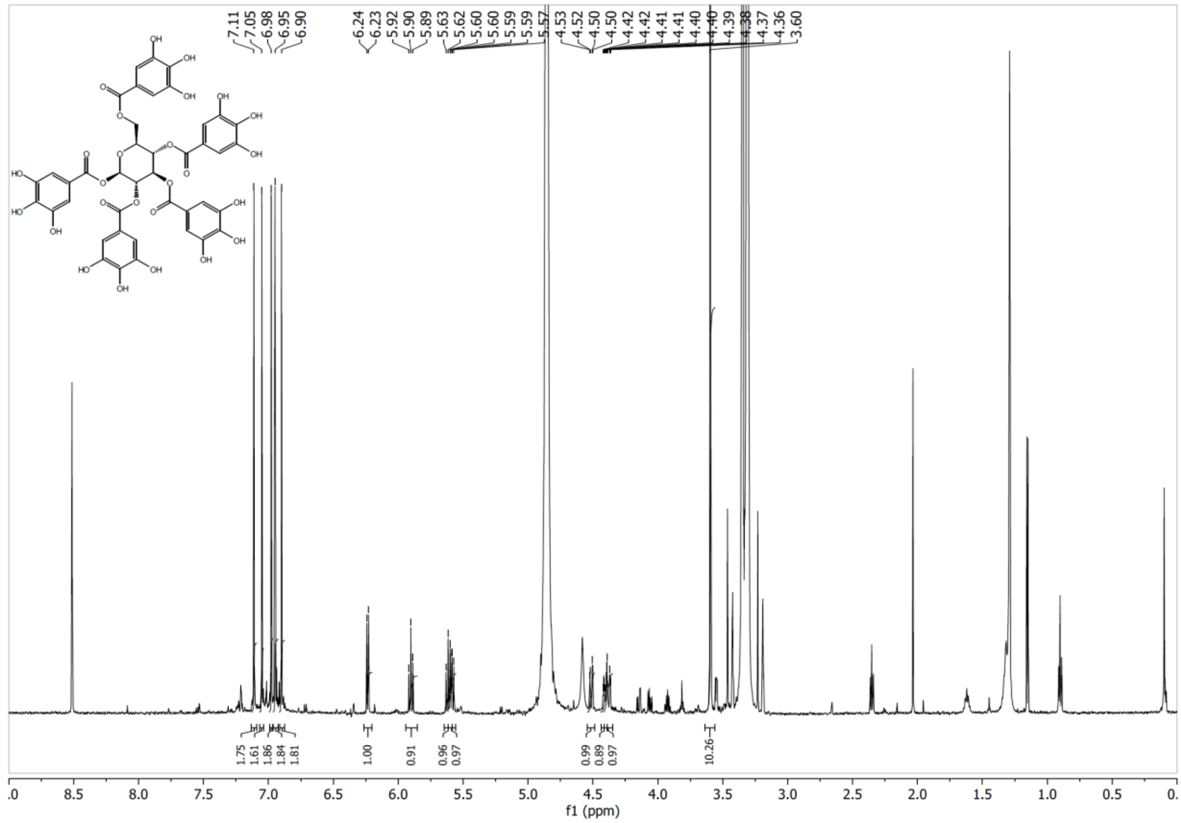

^{1}H NMR spectrum of compound **5** in CD_3OD at 600 MHz.

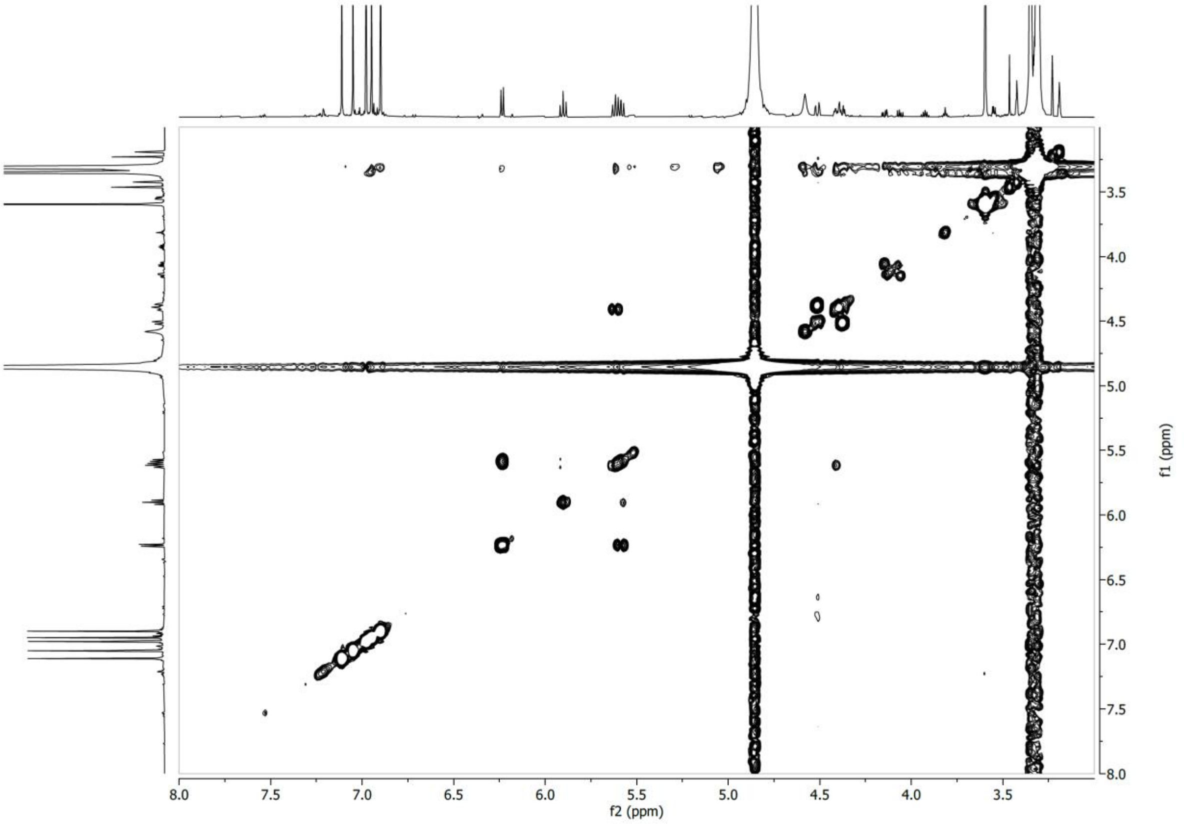

COSY NMR spectrum of compound **5** in CD_3OD.

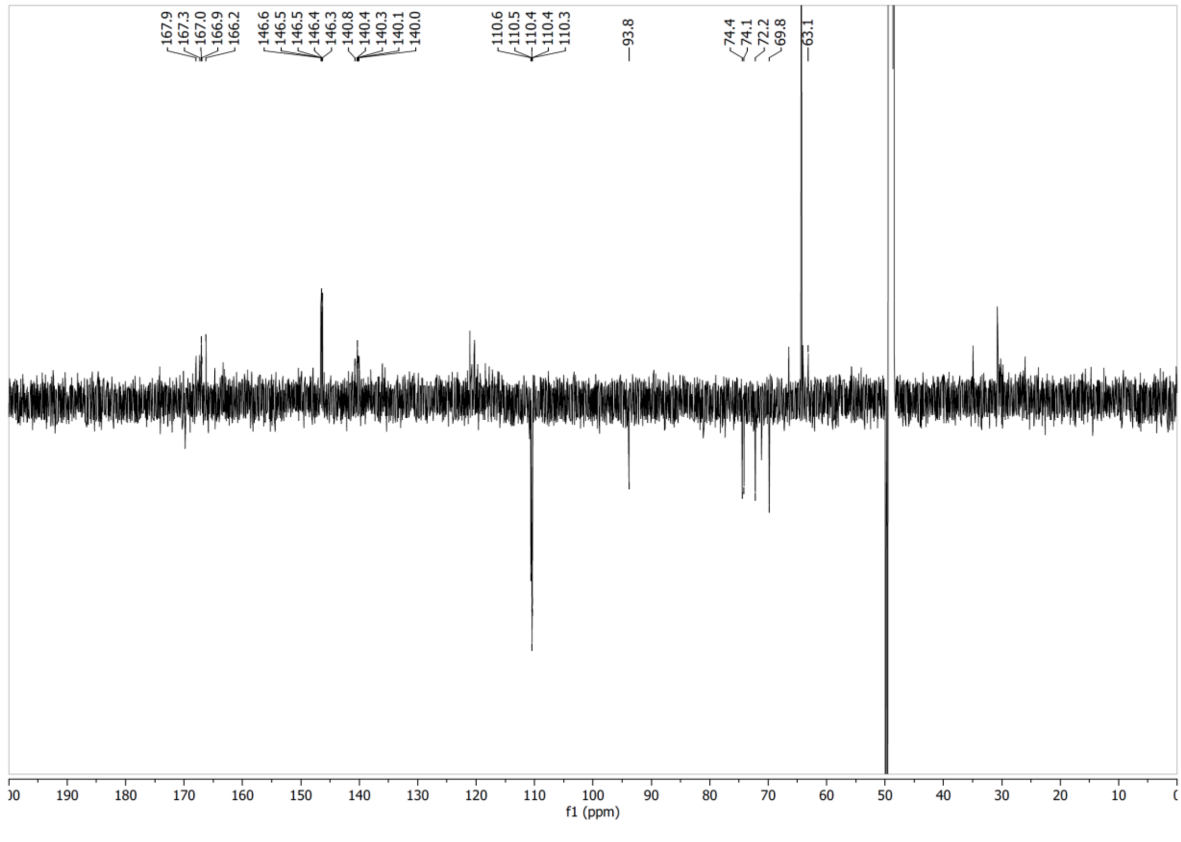

^{13}C-DEPTQ NMR spectrum of compound **5** in CD_3OD at 151 MHz.

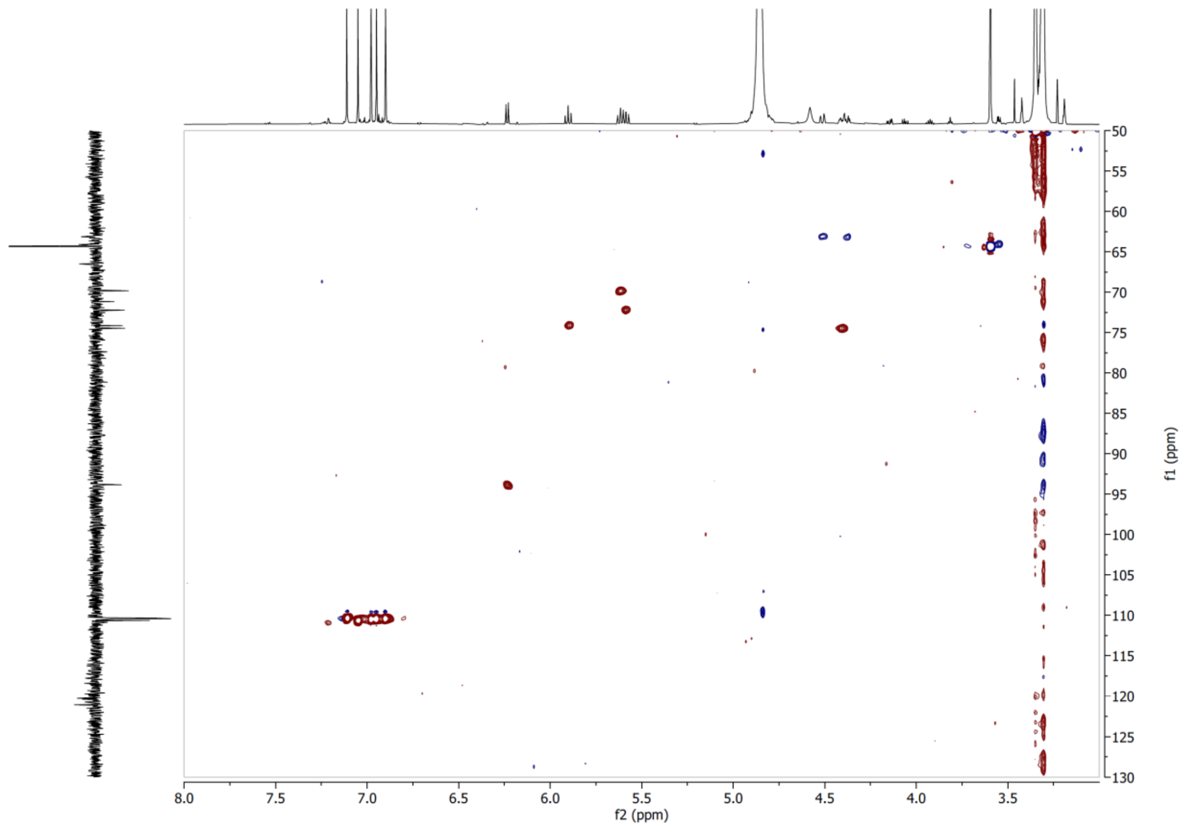

Edited-HSQC NMR spectrum of compound **5** in CD3OD.

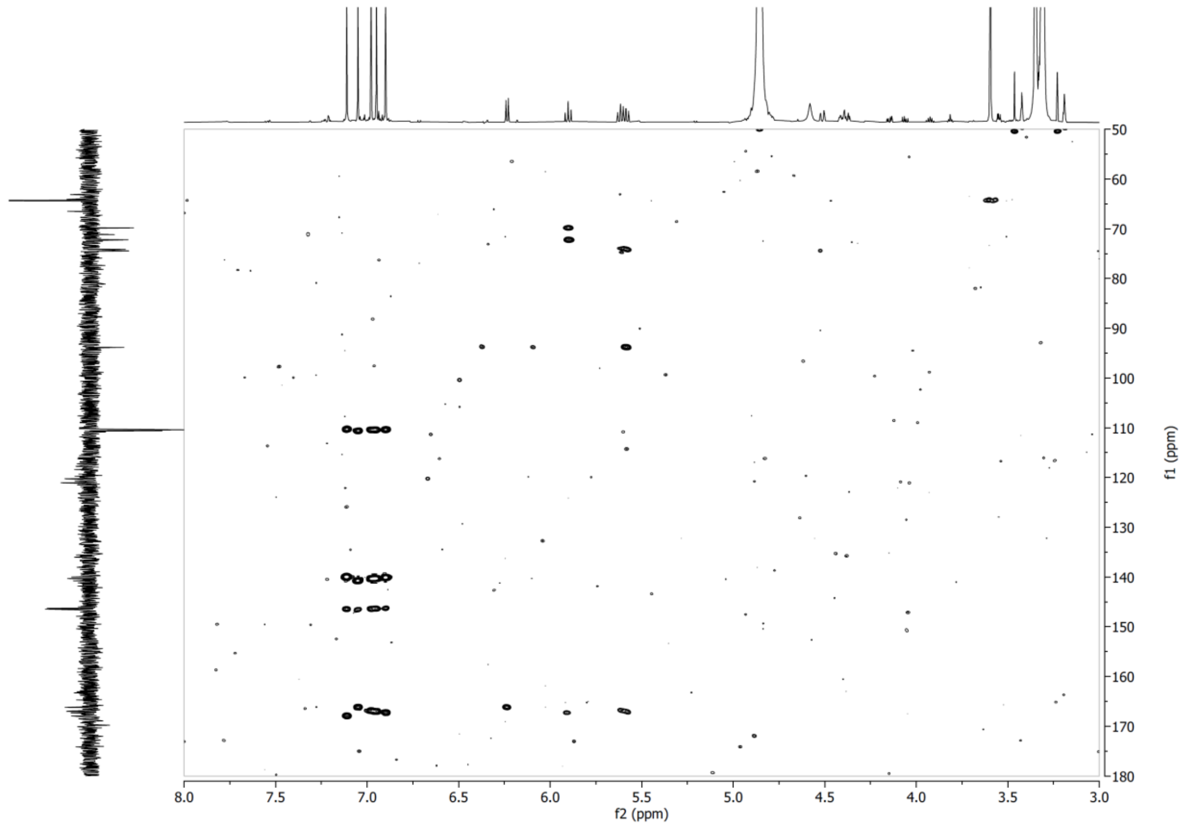

HMBC NMR spectrum of compound **5** in CD3OD.

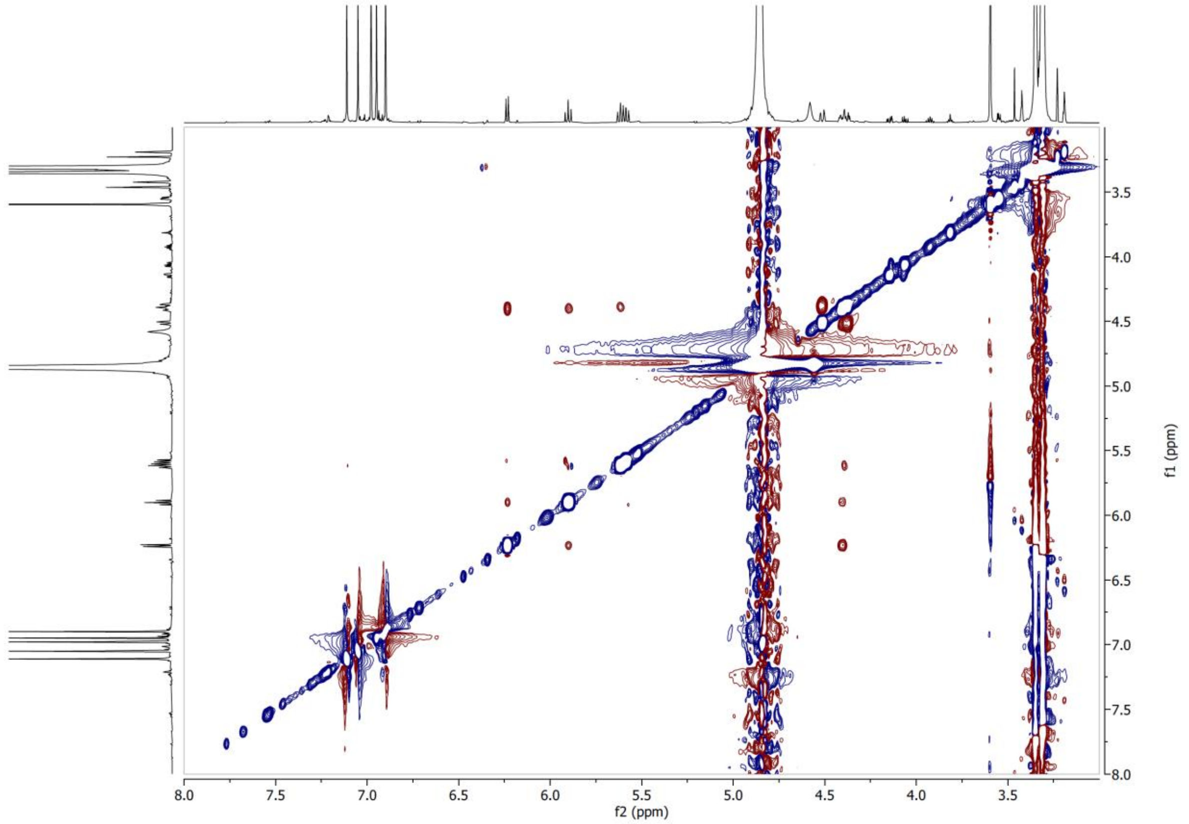

ROESY NMR spectrum of compound **5** in CD_3OD.

Figure S9: NMR data and spectra of compound **6** in DMSO-d_6 at 600 MHz.

Gossypetin-7-*O*-β-glucopyranoside (**6**) (Yang *et al.*, 2003): ^{1}H NMR (DMSO-d_6, 600 MHz) δ 3.18 (1H, t, *J* = 9.8, 8.8 Hz, H-4''), 3.31 (1H, t, *J* = 9.2, 8.8 Hz, H-3''), 3.36 (1H, dd, *J* = 9.2, 7.6 Hz, H-2''), 3.42 (1H, ddd, *J* = 9.8, 6.0, 2.1 Hz, H-5''), 3.48 (1H, dd, *J* = 11.8, 6.0 Hz, H-6''b), 3.73 (1H, dd, *J* = 11.8, 2.1 Hz, H-6''a), 4.91 (1H, d, *J* = 7.6 Hz, H-1''), 6.62 (1H, s, H-6), 6.90 (1H, d, *J* = 8.5 Hz, H-5'), 7.63 (1H, dd, *J* = 8.5, 2.2 Hz, H-6'), 7.76 (1H, d, *J* = 2.2 Hz, H-2'), 11.92 (1H, s, 5OH); ^{13}C NMR (DMSO-d_6, 151 MHz) δ 60.7 (C-6''), 69.7 (C-4''), 73.3 (C-2''), 75.7 (C-3''), 77.3 (C-5''), 97.8 (C-6), 101.4 (C-1''), 104.7 (C-10), 115.3 (C-2'), 120.3 (C-6'), 122.1 (C-1'), 126.8 (C-8), 145.0 (C-3'), 147.9 (C-4'), 150.4 (C-7), 151.5 (C-5). HRESIMS *m/z* 479.0845 [M-H]$^-$ (calcd for $C_{21}H_{19}O_{13}^-$, 479.08257, Δ = 2.9 ppm).

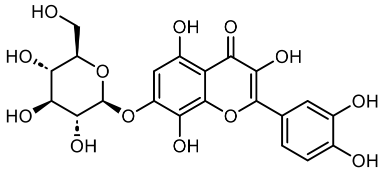

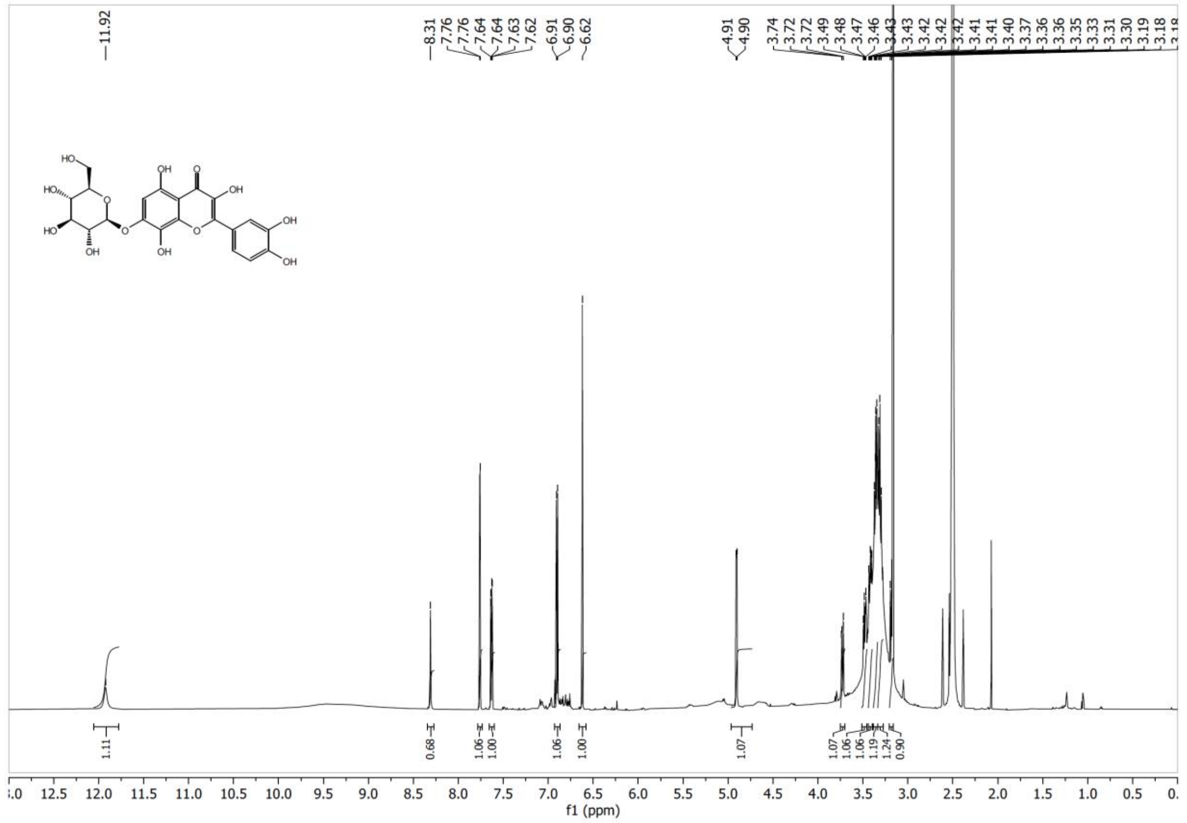

^{1}H NMR spectrum of compound **6** in DMSO-d_6 at 600 MHz.

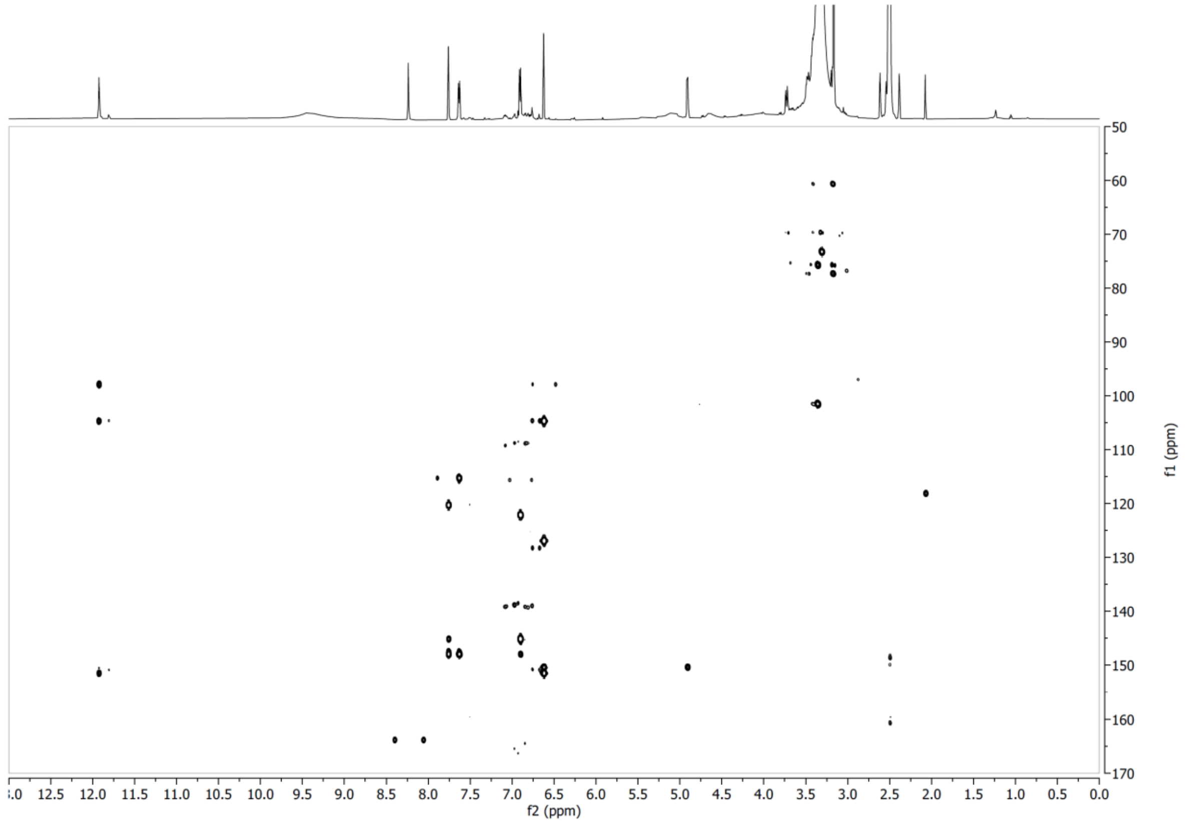

HMBC NMR spectrum of compound **6** in DMSO-d_6.

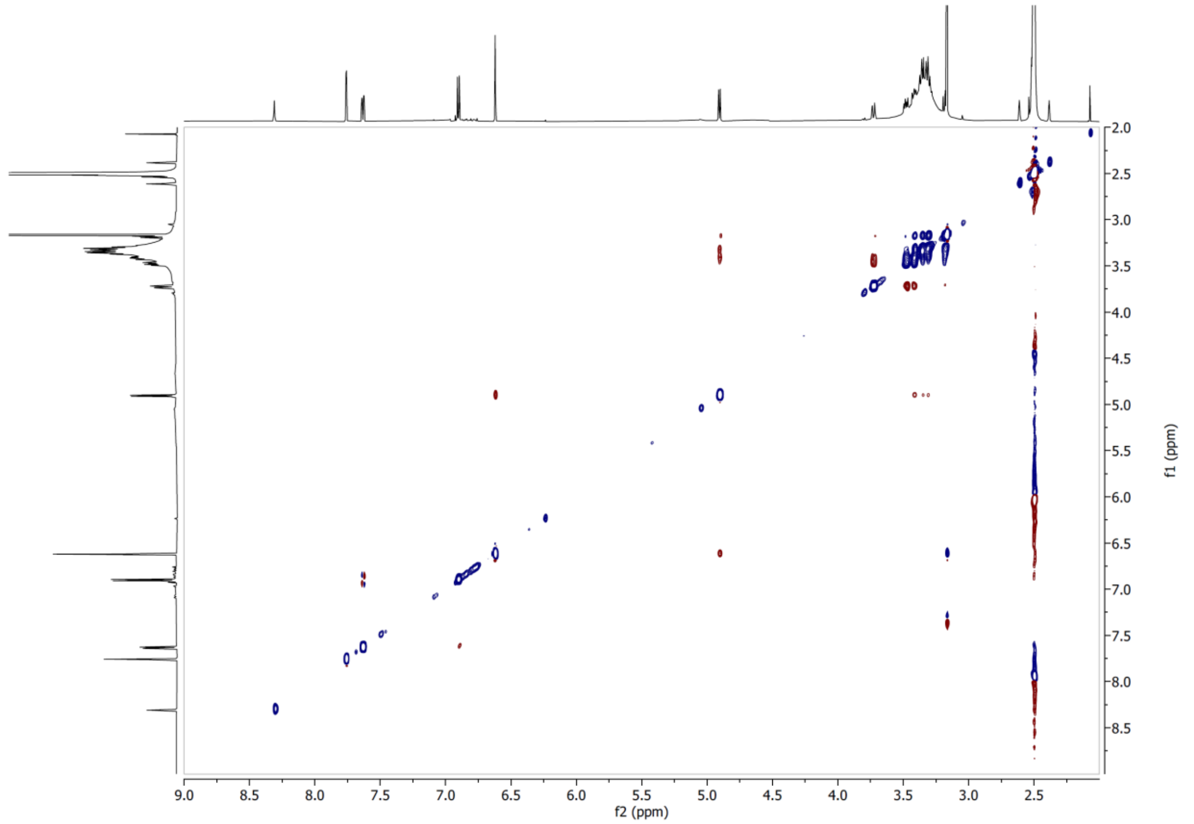

ROESY NMR spectrum of compound **6** in DMSO-d_6.

Figure S10: NMR data and spectra of compound **7** in CD_3OD at 600 MHz.

Hypolaetin-7-*O*-glucoside (**7**) (Zapesochnaya et al., 1973): ^{1}H NMR (CD_3OD, 600 MHz) δ 3.55 (4H, m, H-2'', H-3'', H-4'', H-5''), 3.75 (1H, dd, *J* = 12.2, 5.3 Hz, H-6''b), 3.92 (1H, dd, *J* = 12.2, 2.3 Hz, H-6''a), 5.00 (1H, d, *J* = 7.6 Hz, H-1''), 6.61 (1H, s, H-3), 6.70 (1H, s, H-6), 6.92 (1H, d, *J* = 8.2 Hz, H-5'), 7.50 (1H, dd, *J* = 8.2, 2.3 Hz, H-6'), 7.51 (1H, d, *J* = 2.3 Hz, H-2'); ^{13}C NMR (CD_3OD, 151 MHz) δ 61.9 (C-6''), 71.0 (C-4''), 74.4 (C-2''), 77.2 (C-3''), 78.2 (C-5''), 100.0 (C-6), 102.7 (C-1''), 103.4 (C-3), 107.0 (C-10), 114.1 (C-2'), 116.4 (C-5'), 120.3 (C-6'), 123.4 (C-1'), 128.6 (C-8), 146.8 (C-3'), 150.8 (C-4'), 166.6 (C-2). HRESIMS *m/z* 463.0897 $[M\text{-}H]^-$ (calcd for $C_{21}H_{19}O_{12}^{\,-}$, 463.08765, Δ = 3.3 ppm).

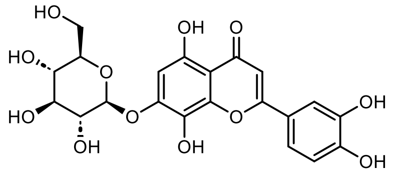

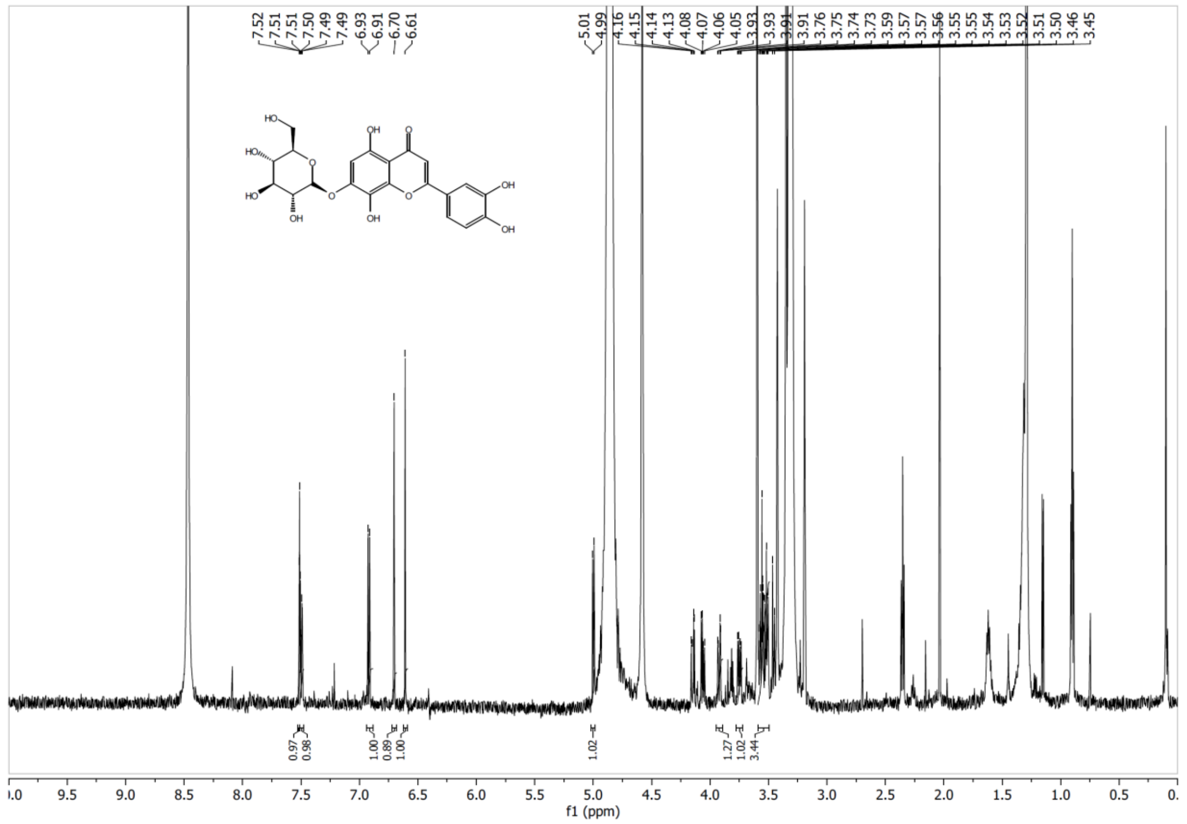

^{1}H NMR spectrum of compound **7** in CD_3OD at 600 MHz.

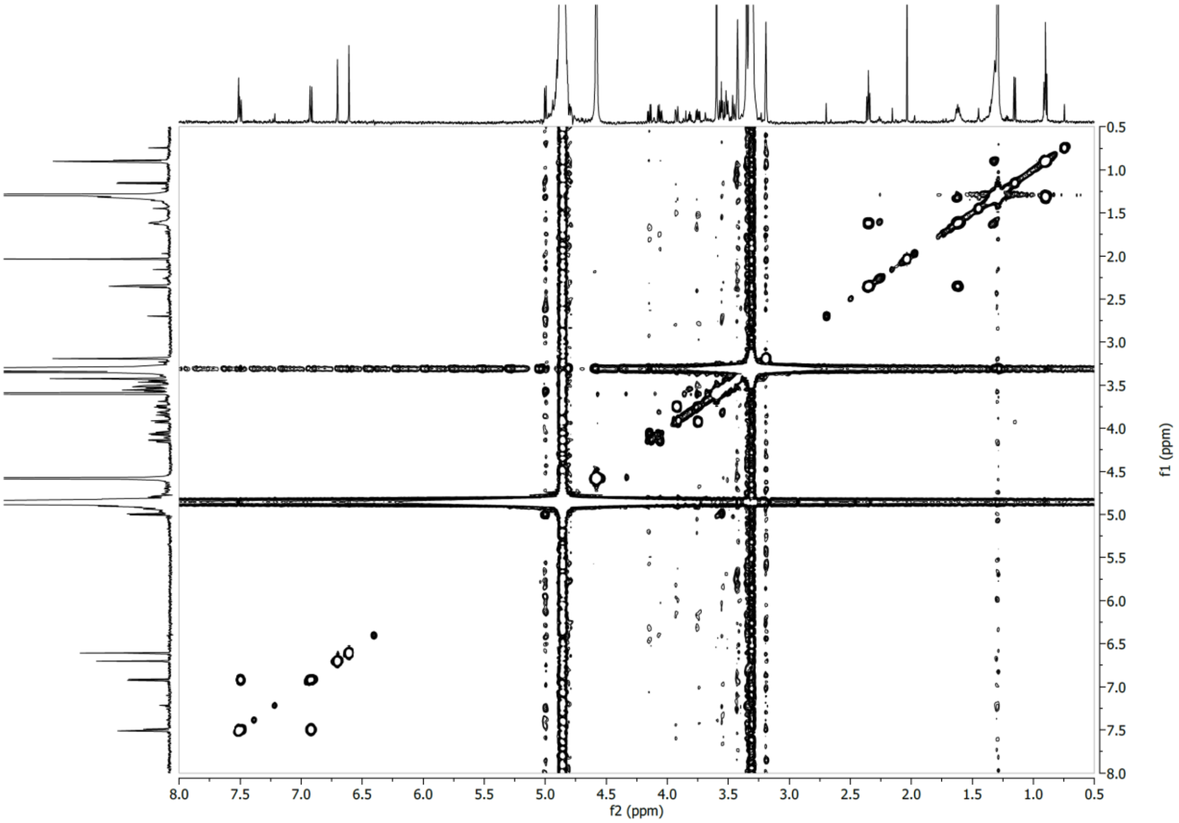

COSY NMR spectrum of compound **7** in CD_3OD.

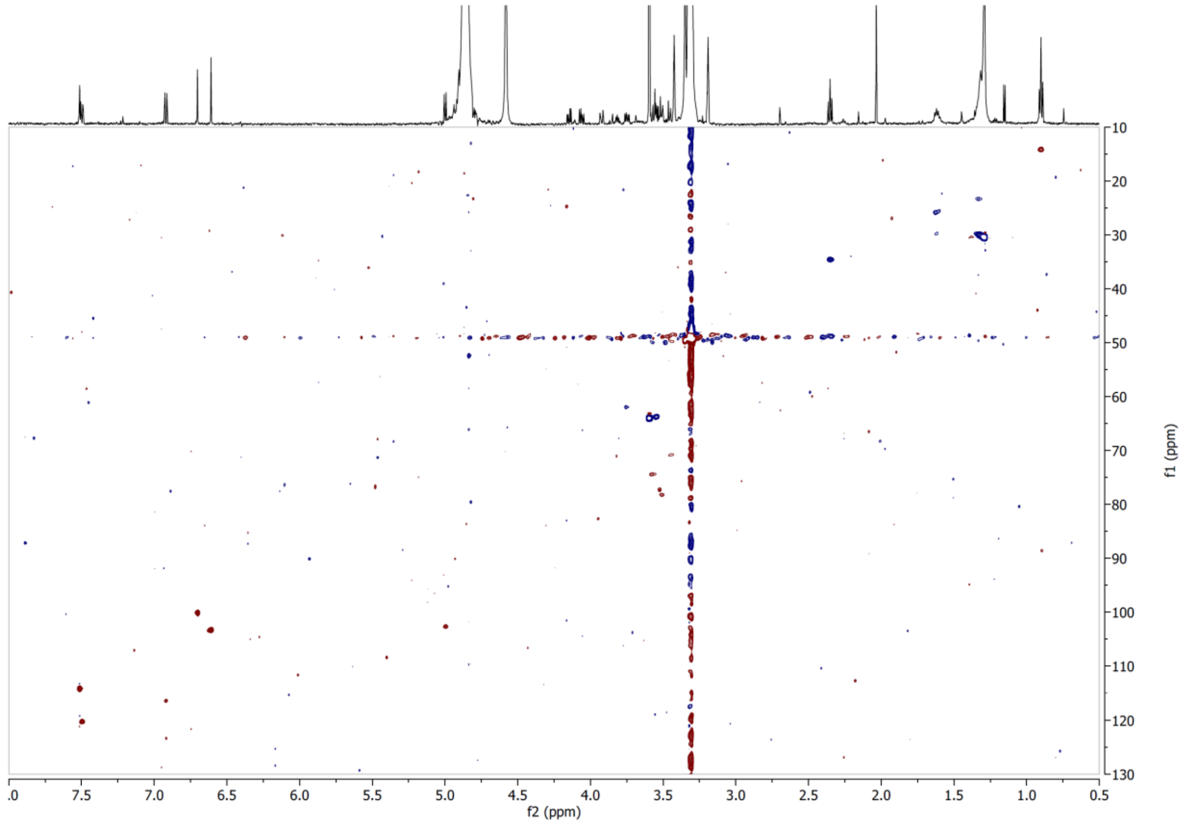

Edited-HSQC NMR spectrum of compound **7** in CD_3OD.

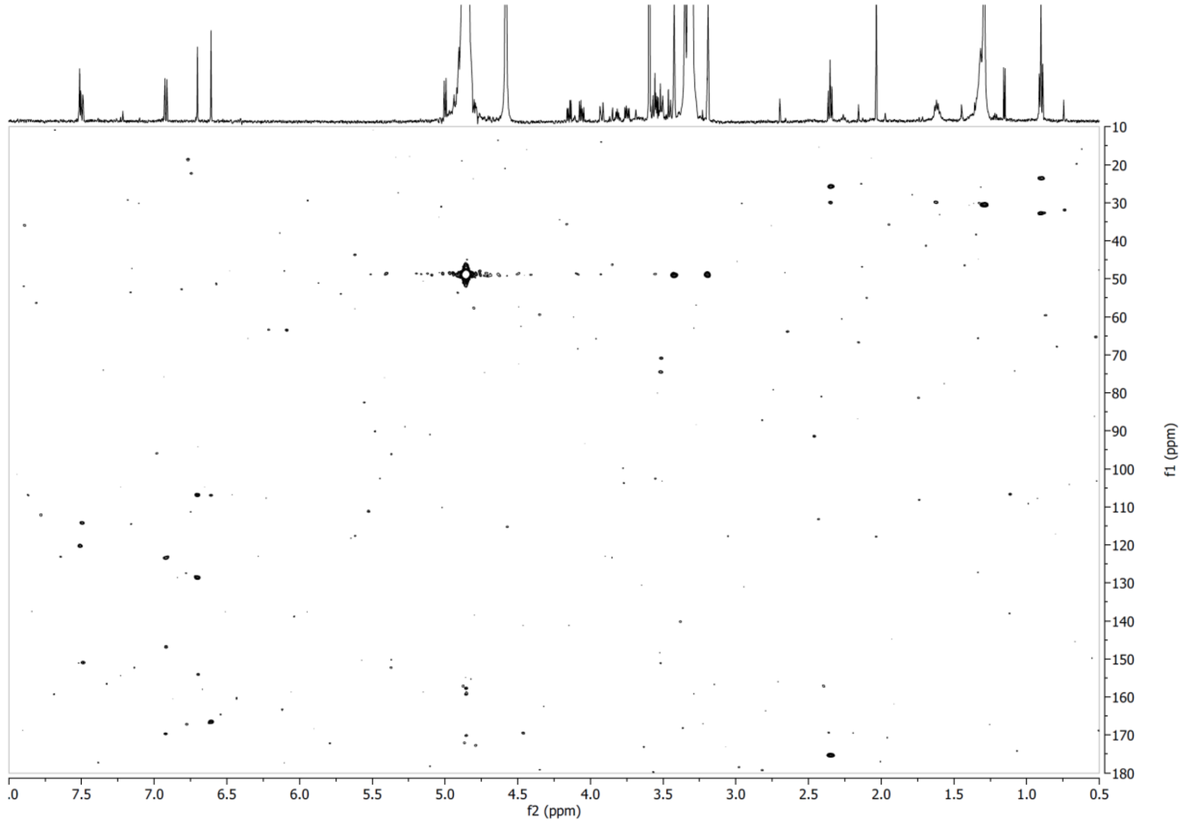

HMBC NMR spectrum of compound **7** in CD_3OD.

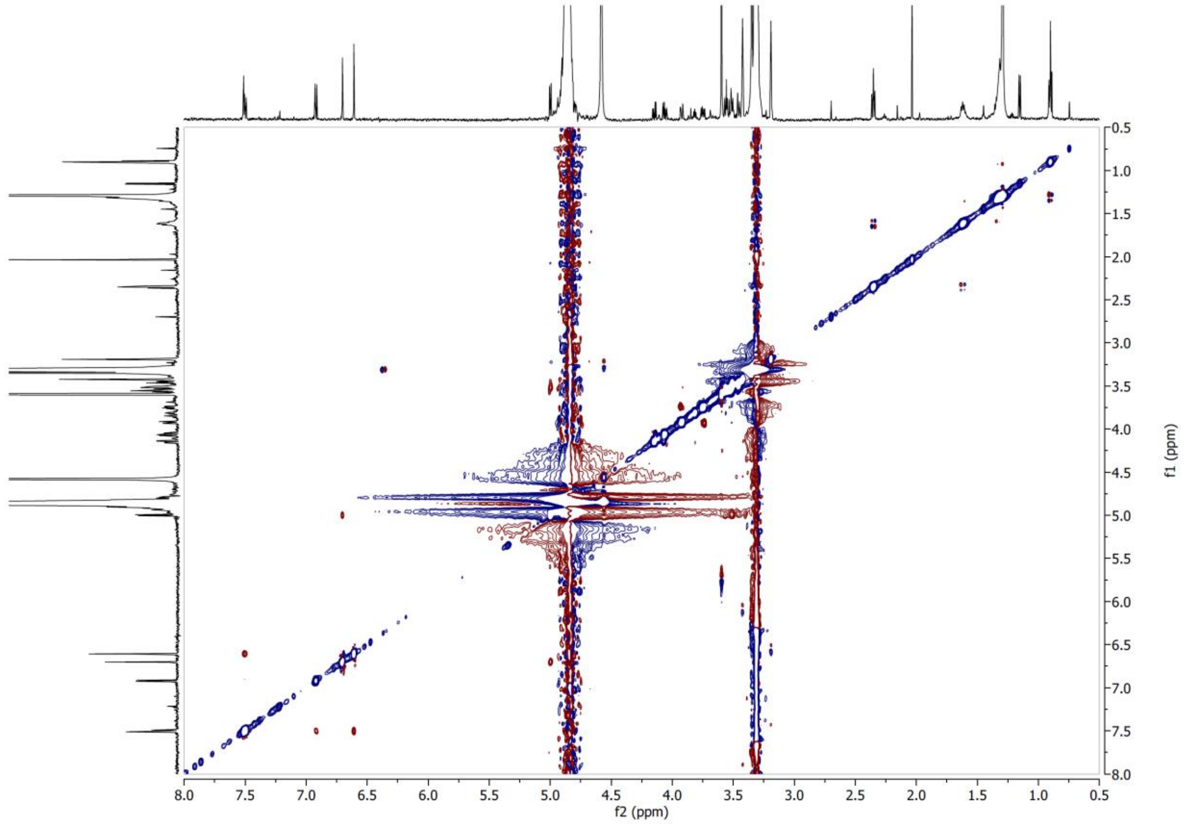

ROESY NMR spectrum of compound **7** in CD_3OD.

Figure S11: NMR data and spectra of compound **8** in CD_3OD at 600 MHz.

5-[(8Z,11Z,14Z)-heptadeca-8,11,14-trienyl] resorcinol (**8**) (Barrow and Capon, 1991): ^{1}H NMR (CD_3OD, 600 MHz) δ 0.97 (3H, t, *J* = 7.5 Hz, CH_3-25), 1.34 (8H, m, H-9, H-10, H-11, H-12), 1.57 (2H, m, H-8), 2.08 (4H, m, H-13, H-24), 2.44 (2H, m, H-7), 2.81 (4H, m, H-16, H-19), 5.35 (6H, m, H-14, H-15, H-17, H-18, H-22, H-23), 6.08 (1H, t, *J* = 2.2 Hz, H-4), 6.12 (2H, d, *J* = 2.2 Hz, H-2, H-6); ^{13}C NMR (CD_3OD, 151 MHz) δ 14.6 (CH_3-25), 21.5 (C-24), 26.4 (C-16), 26.5 (C-19), 28.2 (C-13), 30.3 (C-9), 30.3 (C-11), 30.5 (C-10), 30.7 (C-12), 32.4 (C-8), 37.0 (C-7), 100.9 (C-4), 107.9 (C-2, C-6), 128.2 (C-22), 128.8 (C-15), 129.2 (C-18), 129.2 (C-17), 131.1 (C-14), 132.7 (C-23), 146.3 (C-1), 159.3 (C-3, C-5). HRESIMS *m/z* 341.24808 $[M-H]^-$ (calcd for $C_{23}H_{33}O_2^{\,-}$, 341.24806, Δ = 1.7 ppm).

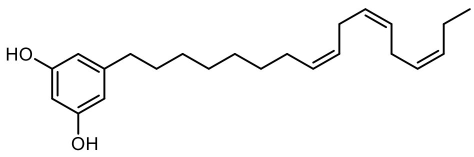

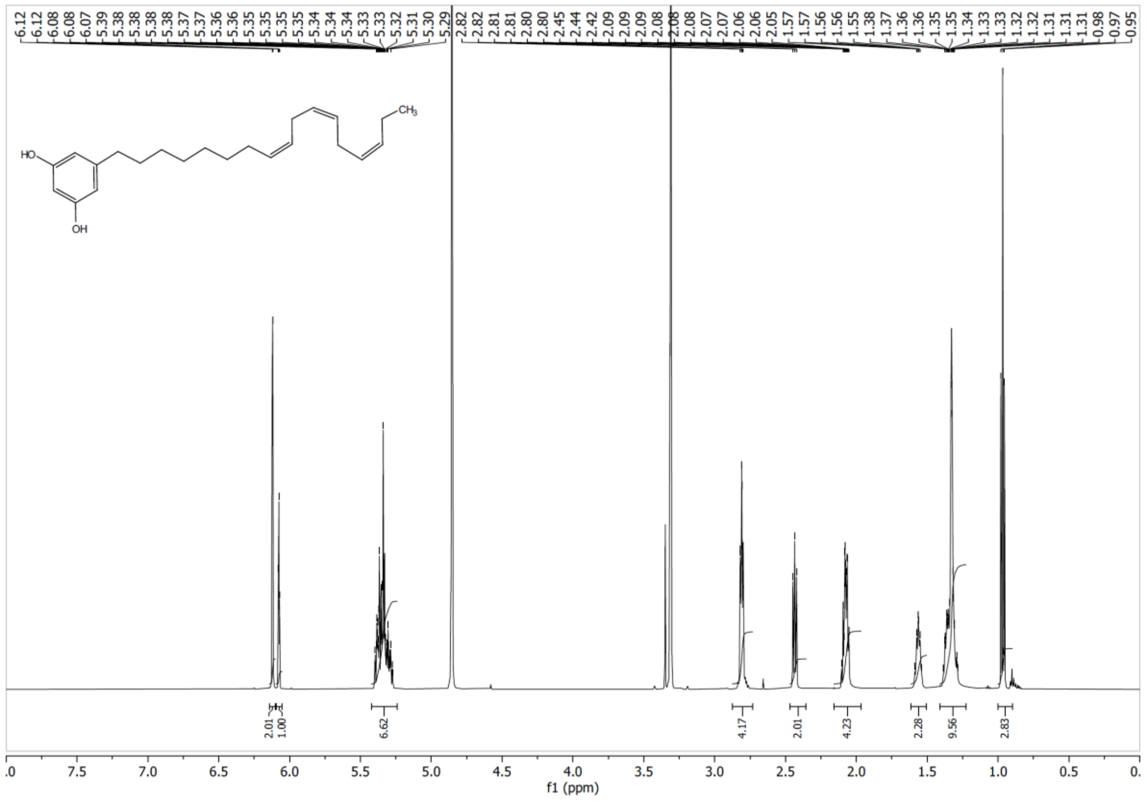

^{1}H NMR spectrum of compound **8** in CD_3OD at 600 MHz.

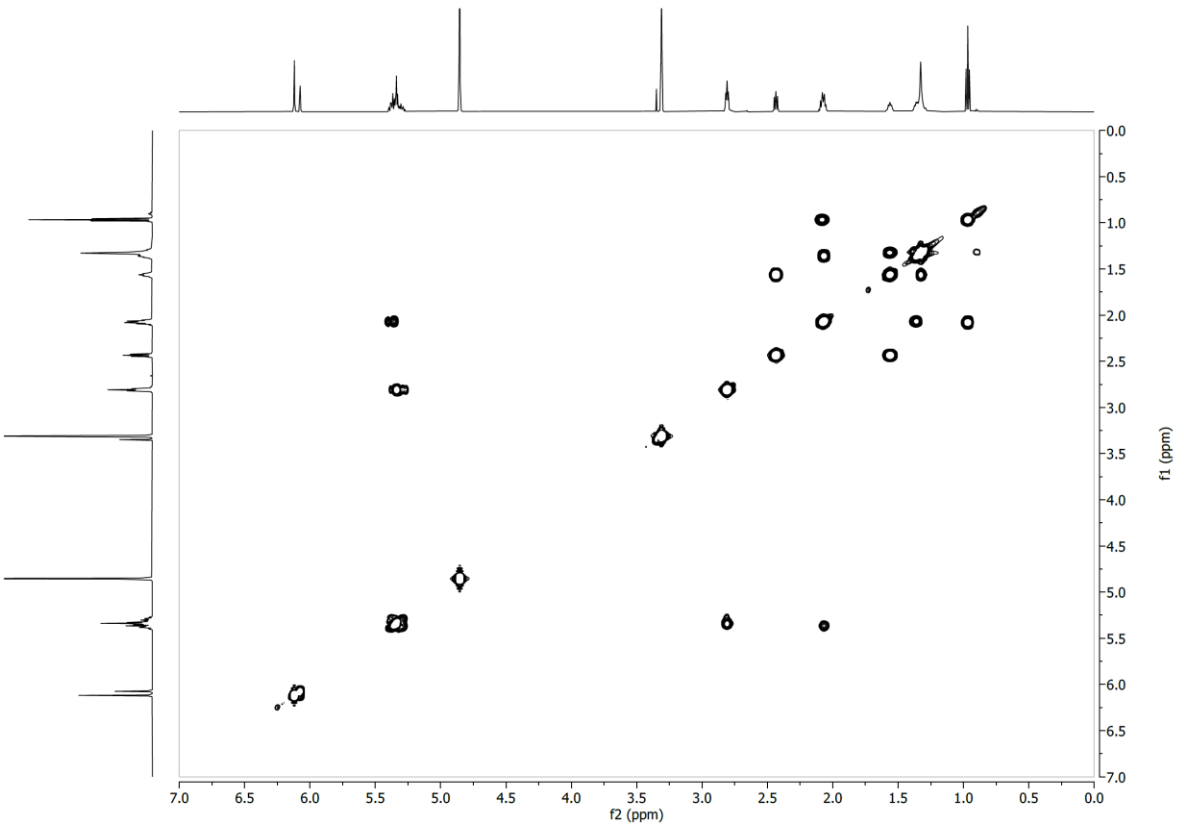

COSY NMR spectrum of compound **8** in CD_3OD.

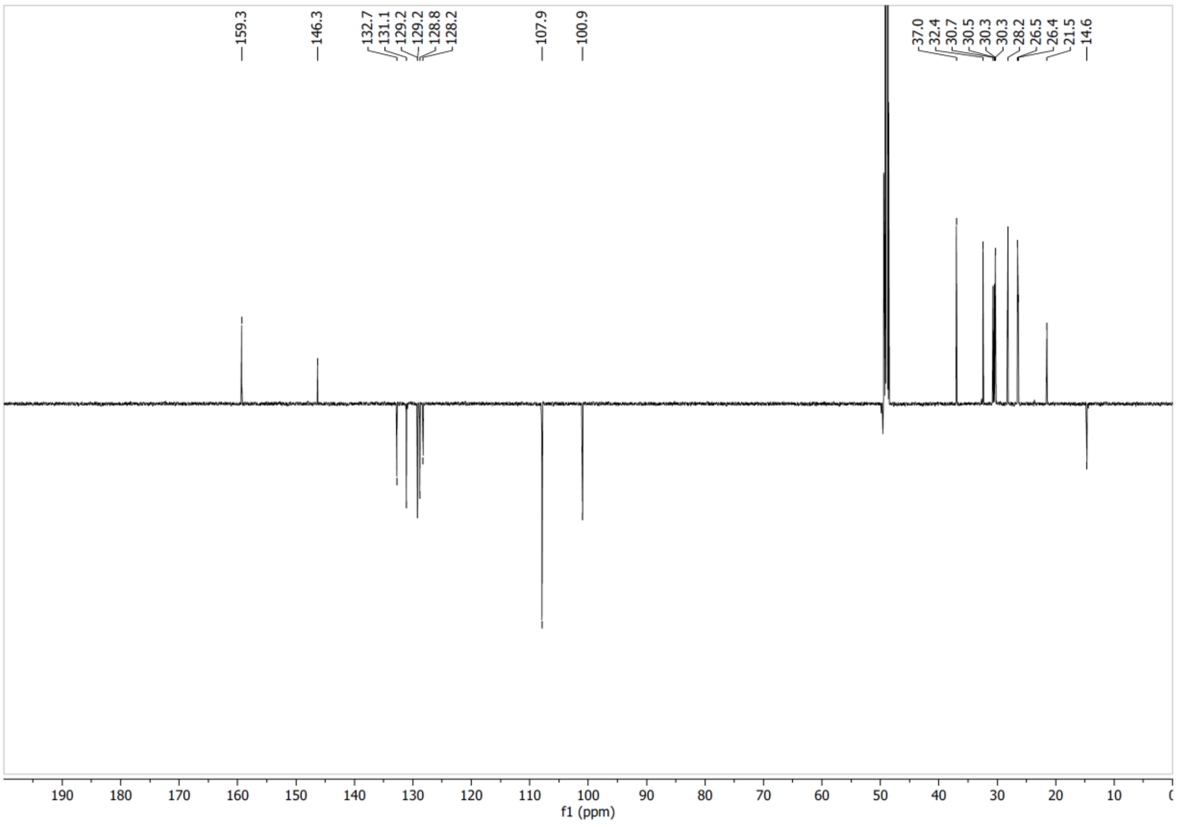

^{13}C-DEPTQ NMR spectrum of compound **8** in CD_3OD at 151 MHz.

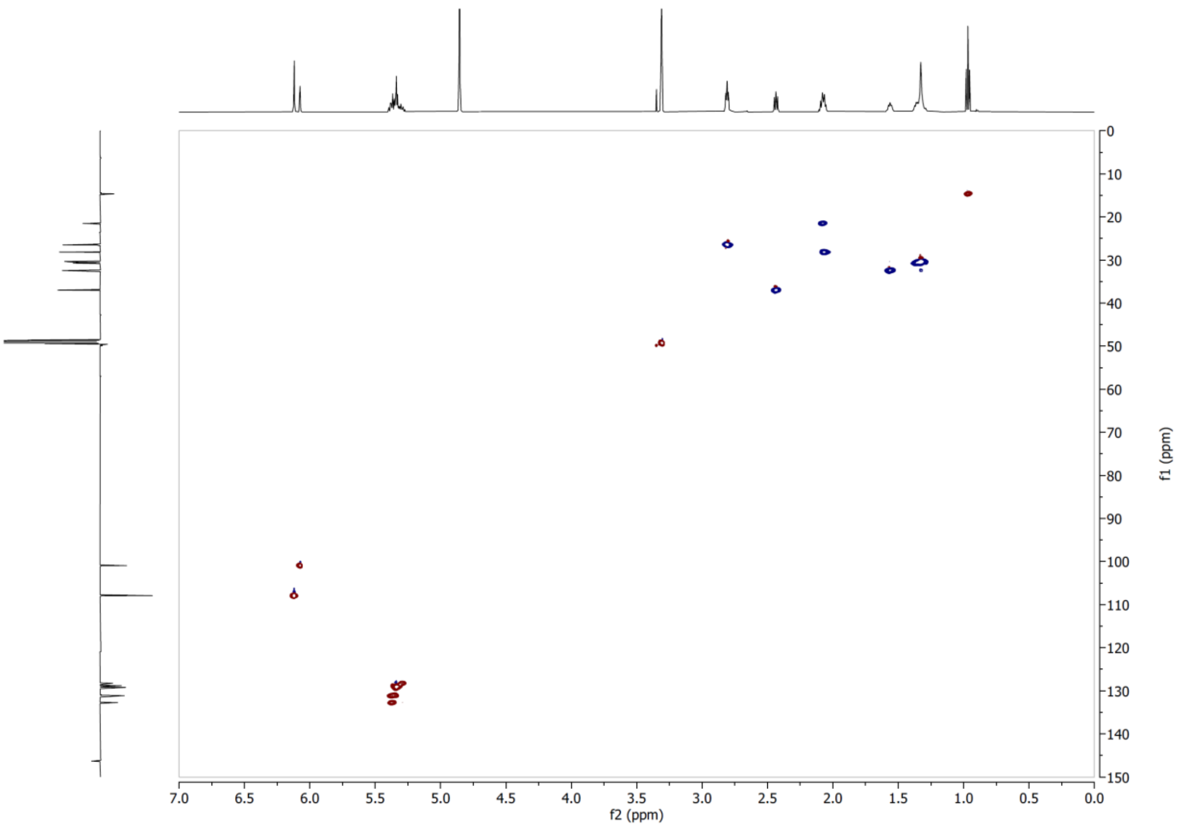

Edited-HSQC NMR spectrum of compound **8** in CD_3OD.

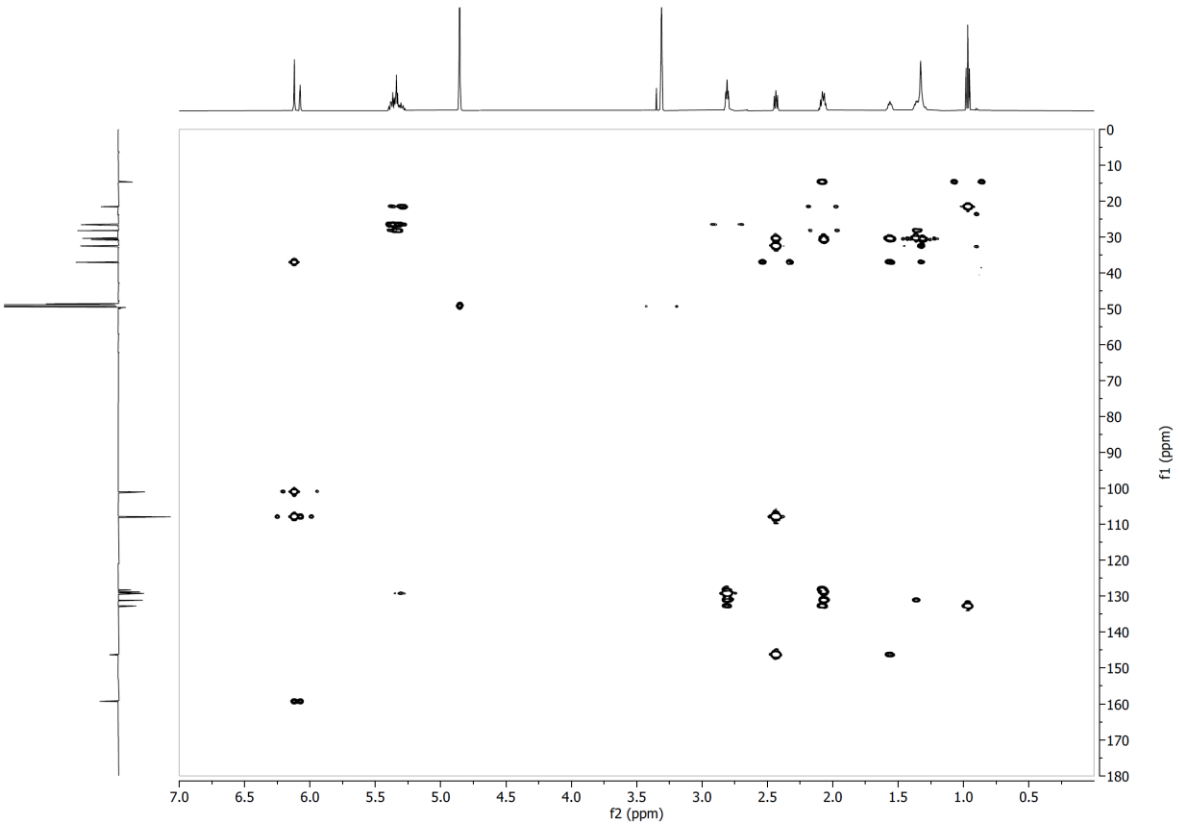

HMBC NMR spectrum of compound **8** in CD_3OD.

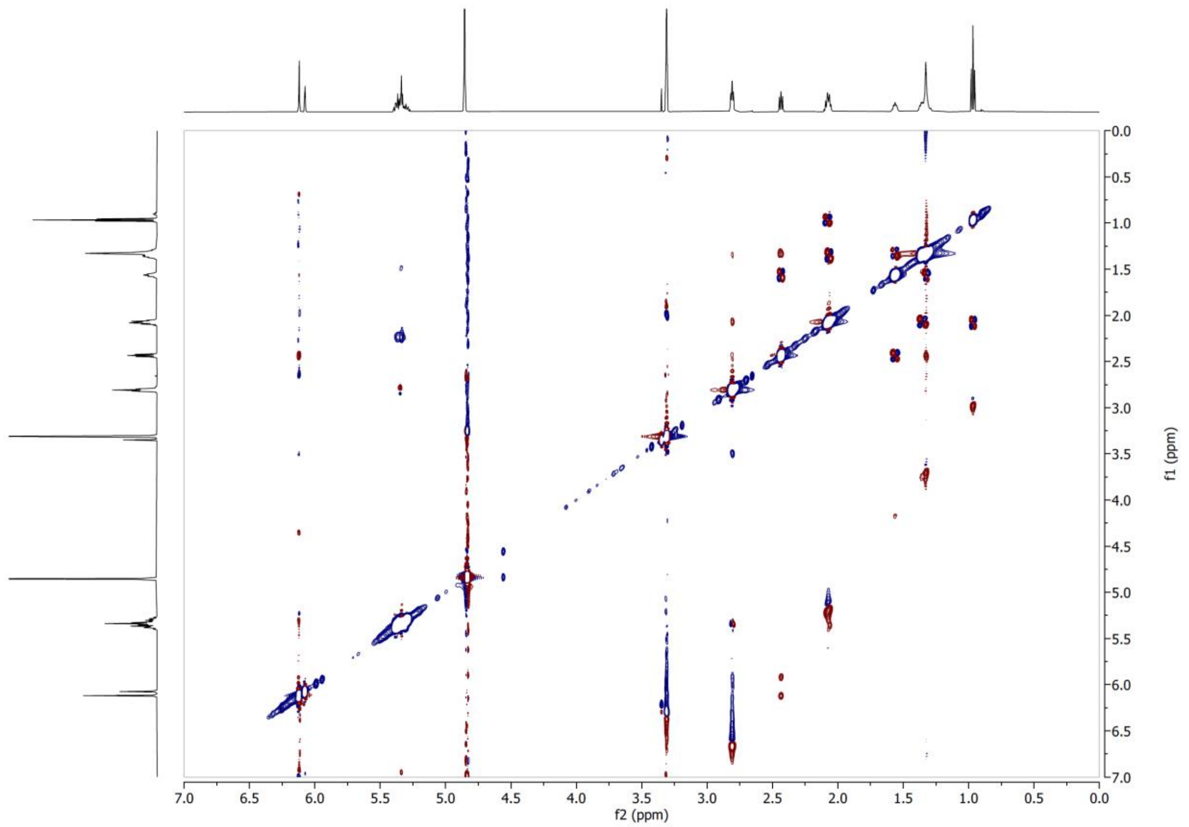

ROESY NMR spectrum of compound **8** in CD_3OD.

Figure S12: UHPLC-HRMS chromatogram showing the presence of compounds **1** and **7** in the decoction of *N. lotus* and correspondence of their MS/MS spectra.

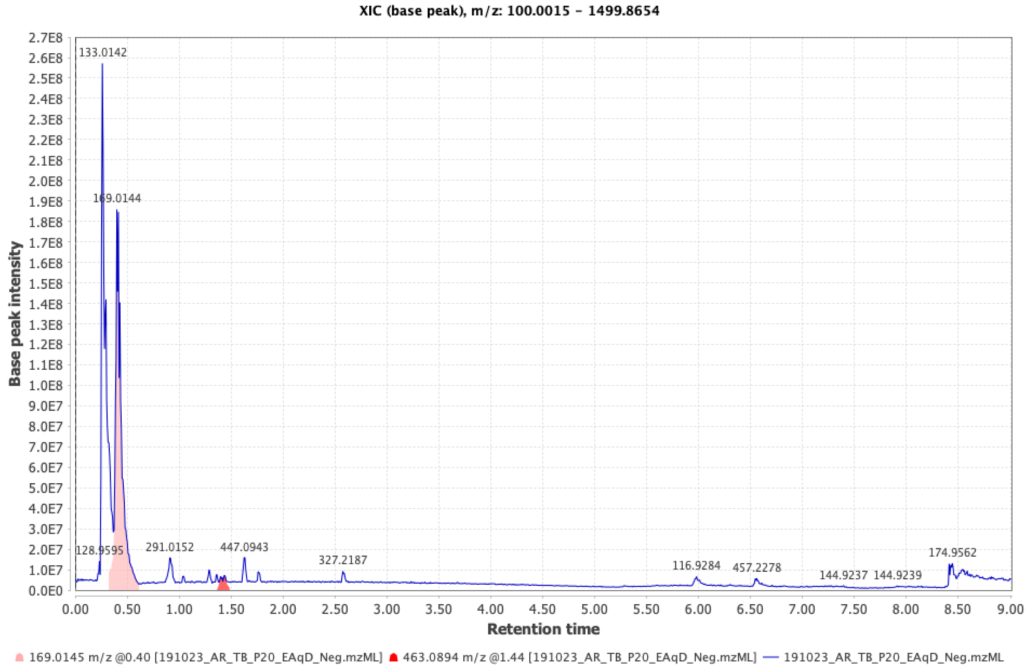

Chromatogram of the decoction of *N. lotus* highlighting compounds **1** and **7**.

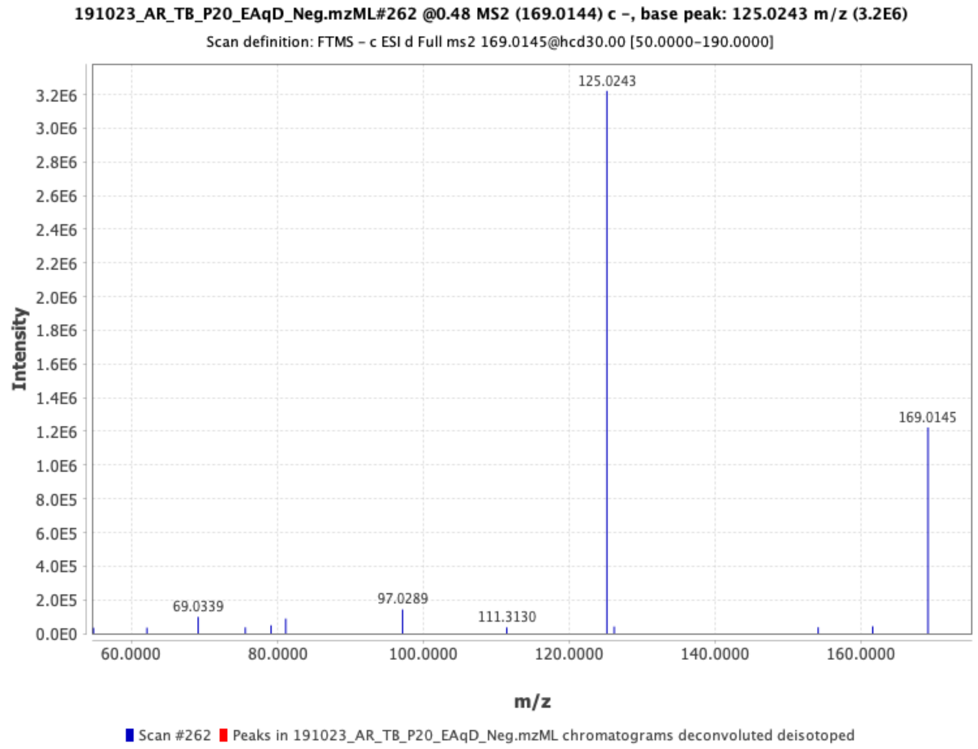

MS/MS spectra of compound **1** in the decoction of *N. lotus*.

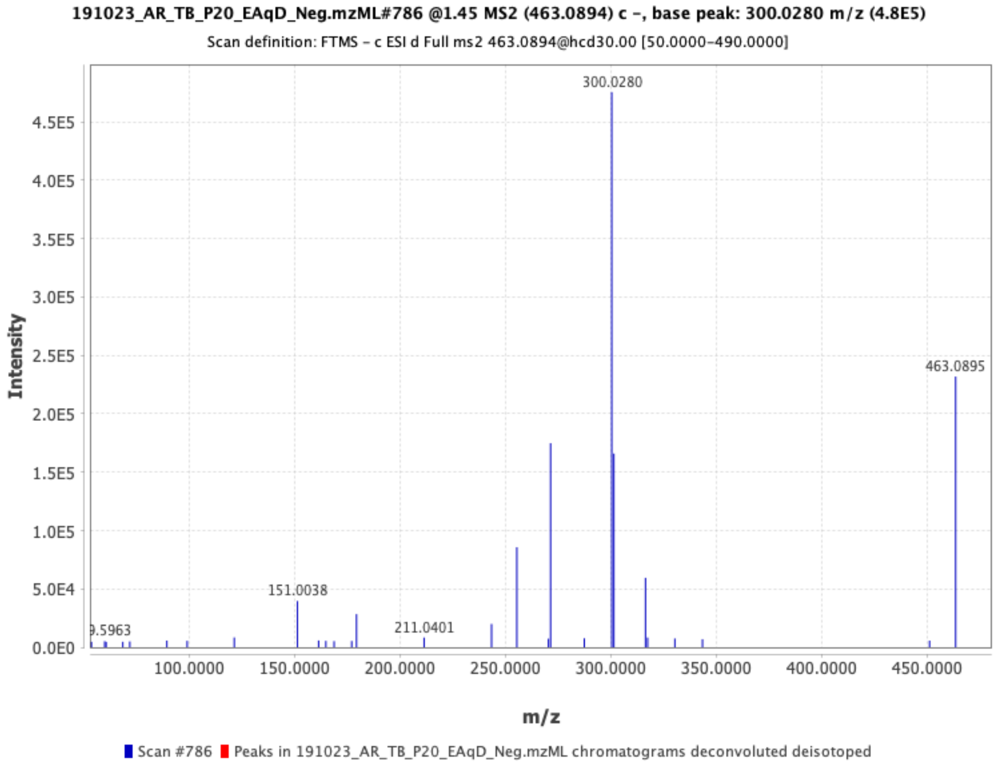

MS/MS spectra of compound **7** in the decoction of *N. lotus*.

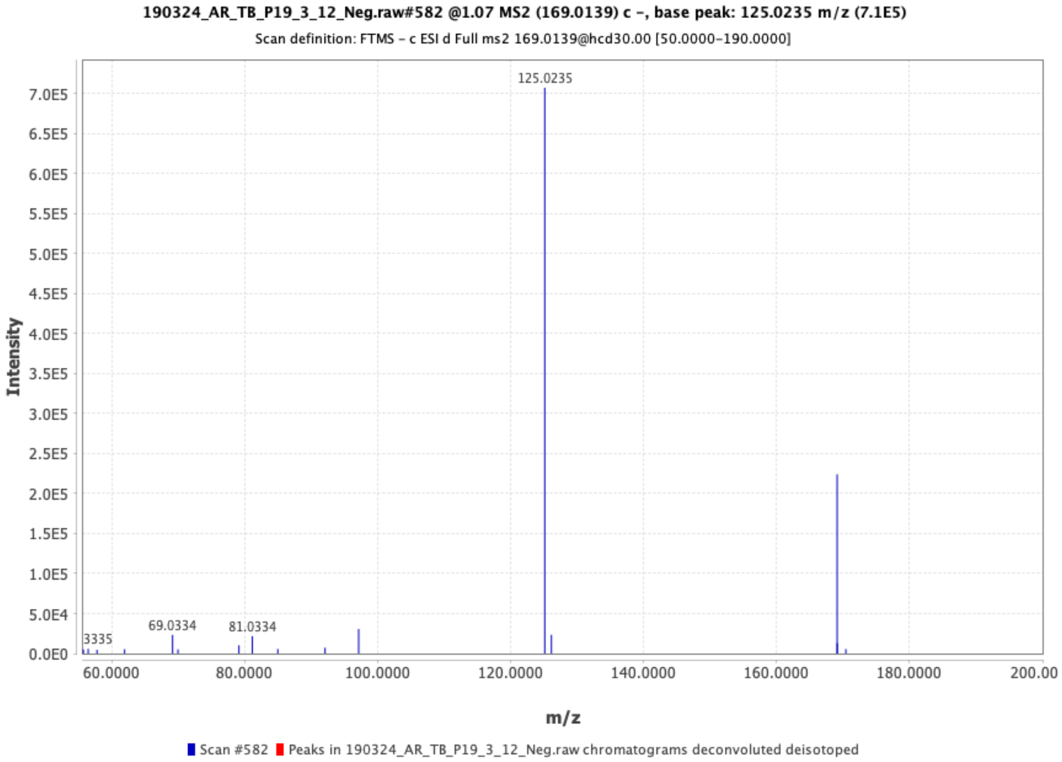

MS/MS spectra of compound **1**.

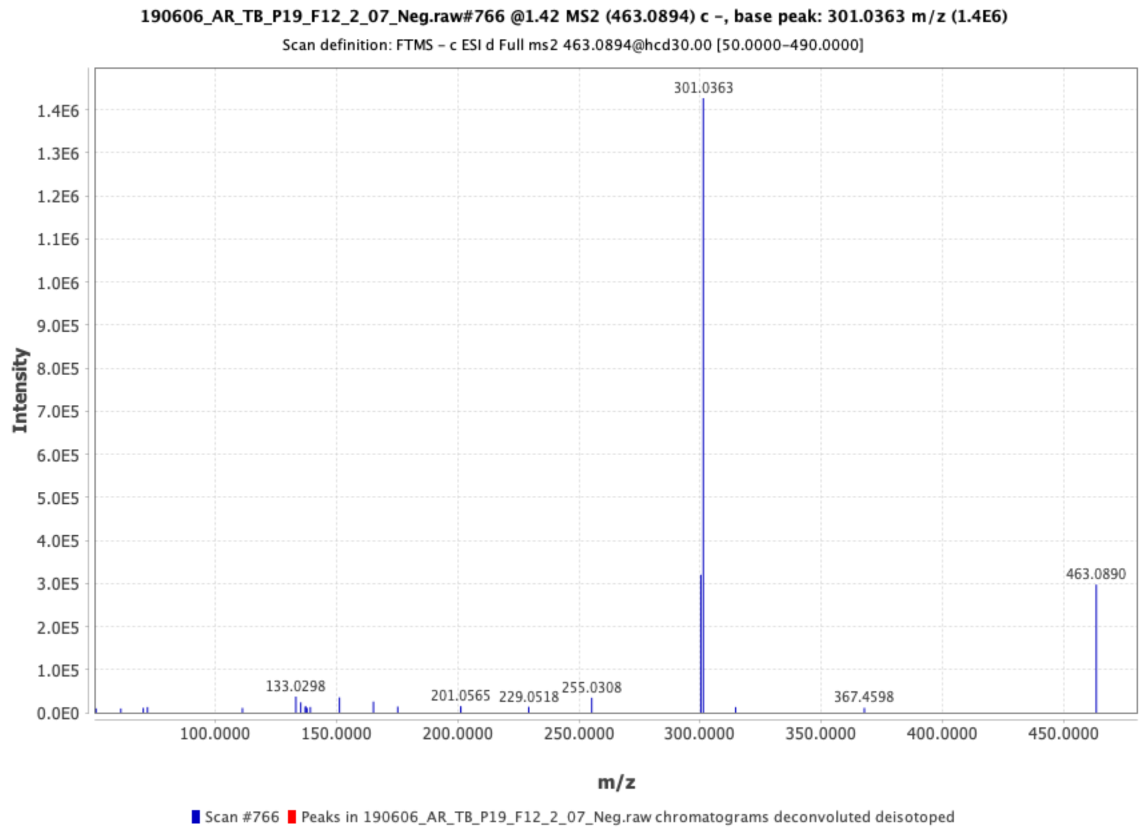

MS/MS spectra of compound **7**.

Figure S13: UHPLC-HRMS chromatogram showing the presence of compounds **1**, **2**, **5**, **6** and **7** in the decoction of *B. schreberi*.

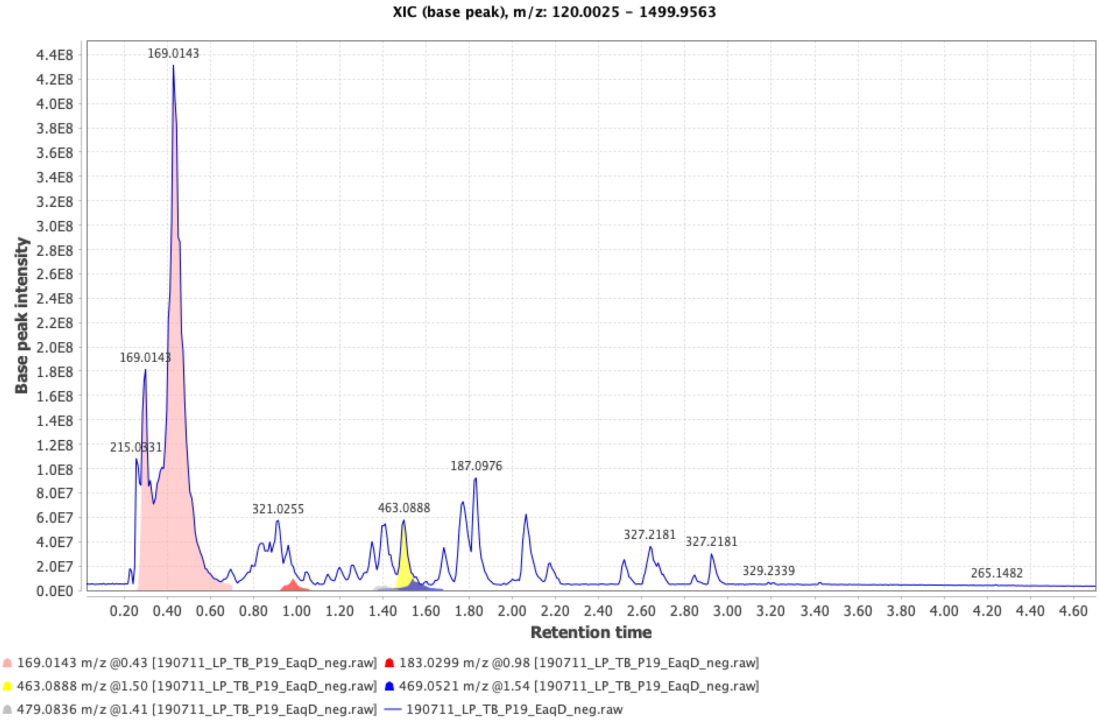

Chromatogram of the decoction of *B. schreberi* highlighting compounds **1, 2, 5, 6** and **7**.

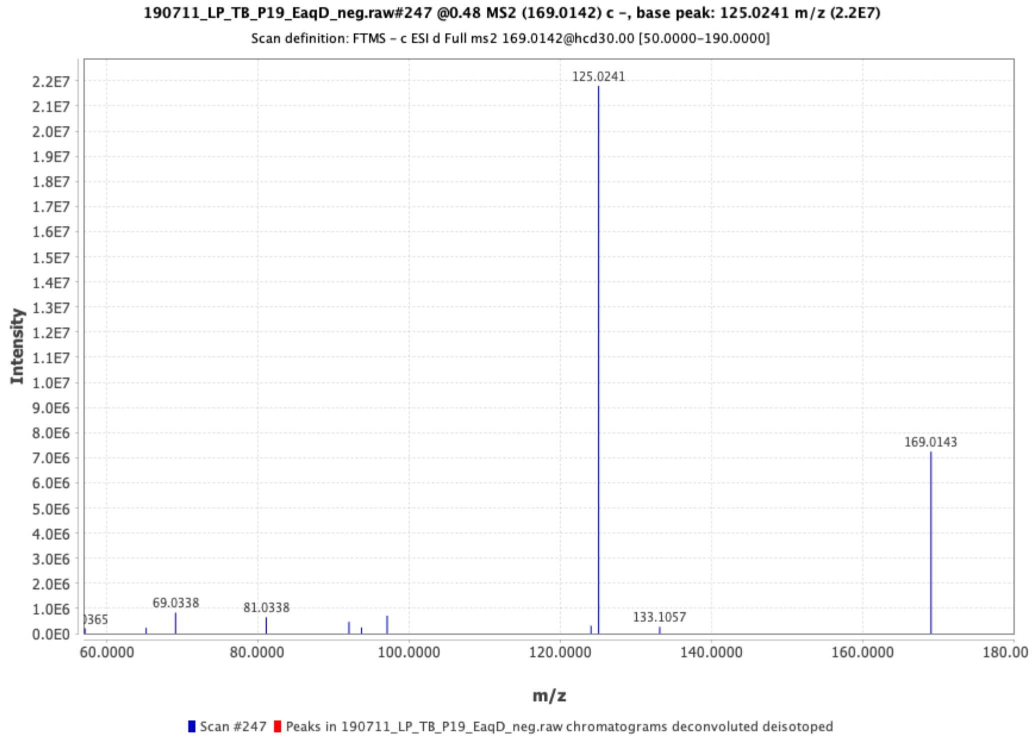

MS/MS spectra of compound **1** in the decoction of *B. schreberi*.

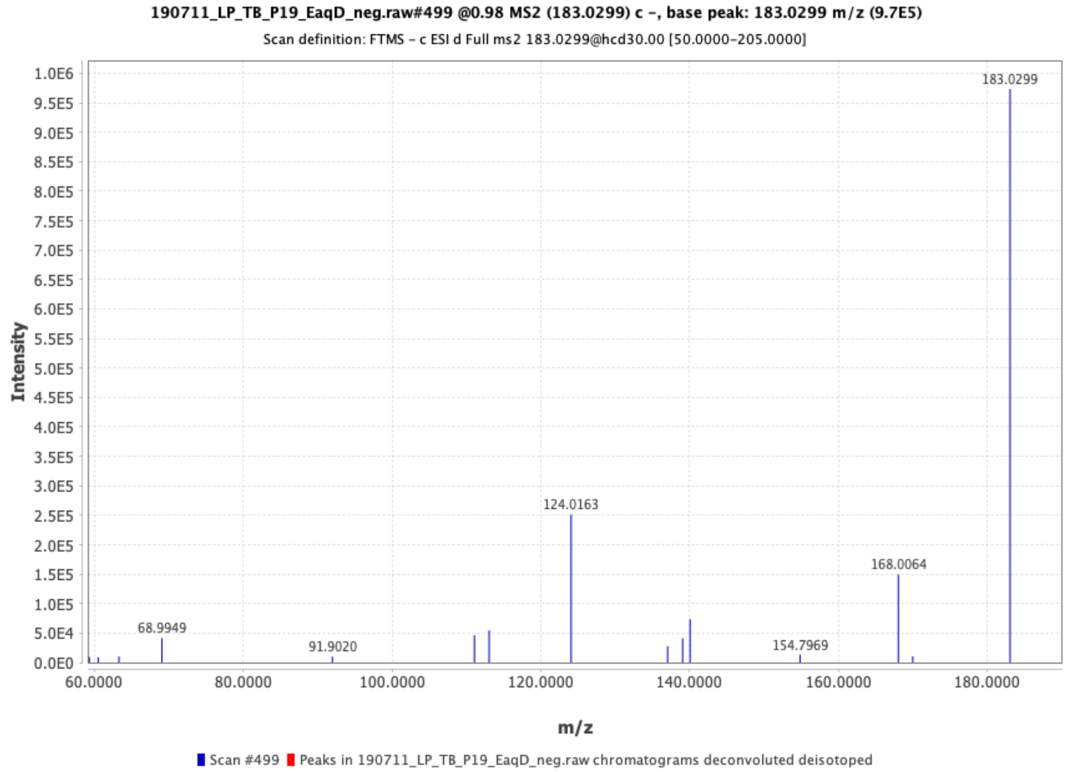

MS/MS spectra of compound **2** in the decoction of *B. schreberi*.

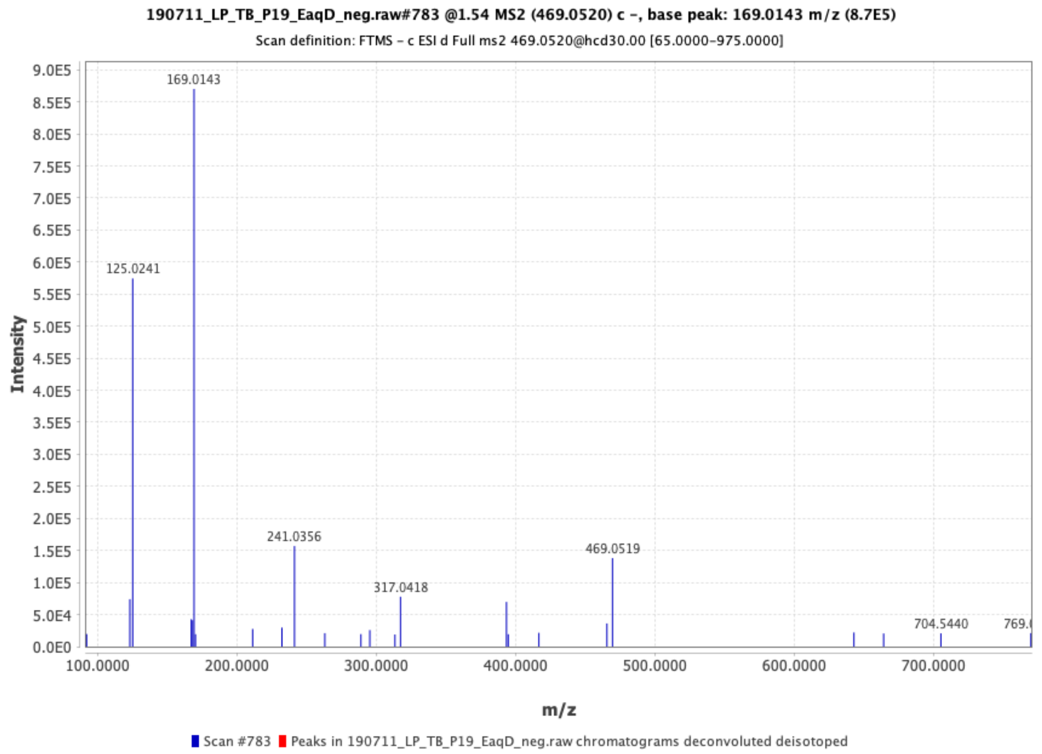

MS/MS spectra of compound **5** in the decoction of *B. schreberi*, only observed as *m/z* 469.0521 $[M-H]^{2-}$.

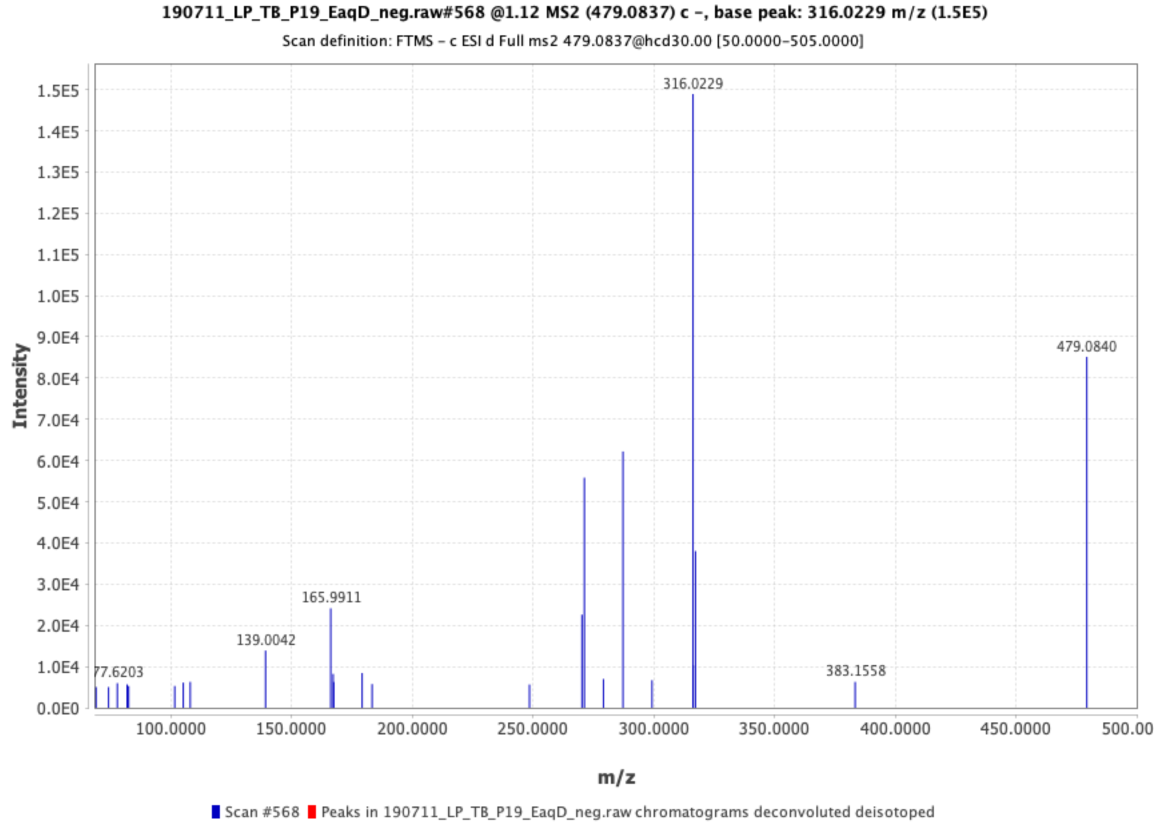

MS/MS spectra of compound **6** in the decoction of *B. schreberi*.

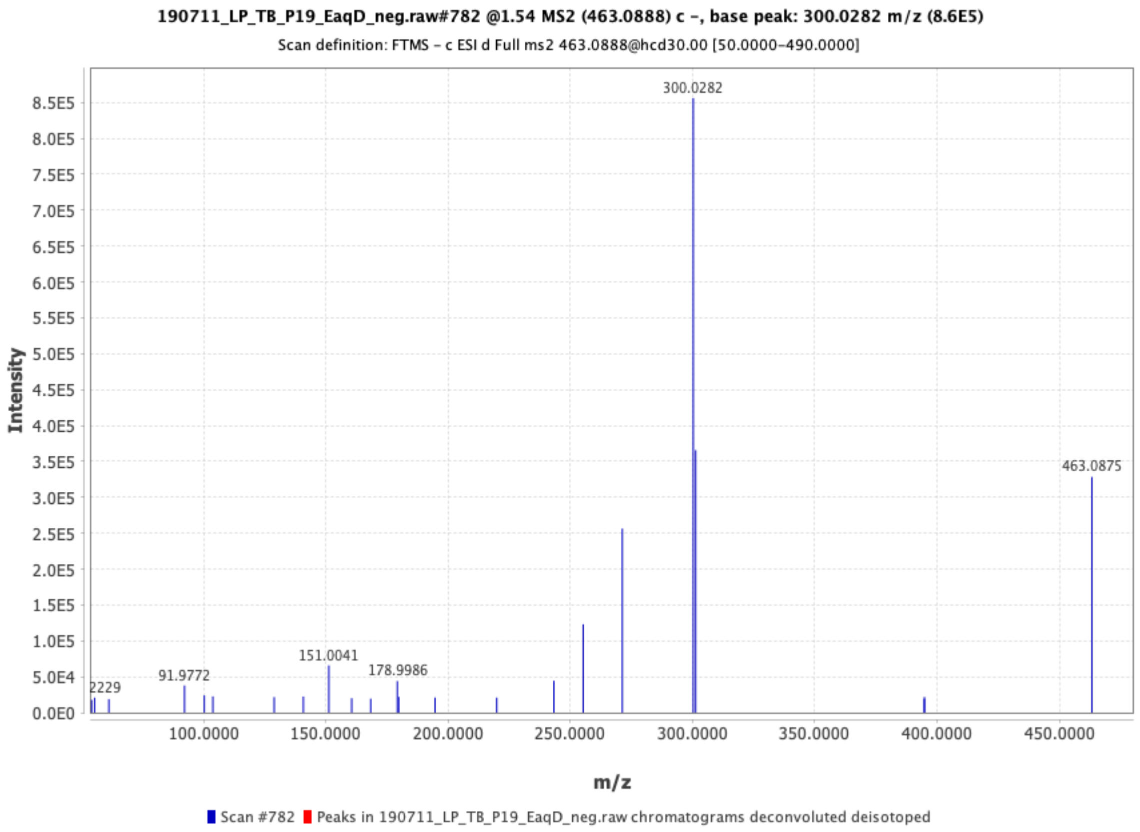

MS/MS spectra of compound **7** in the decoction of *B. schreberi.*

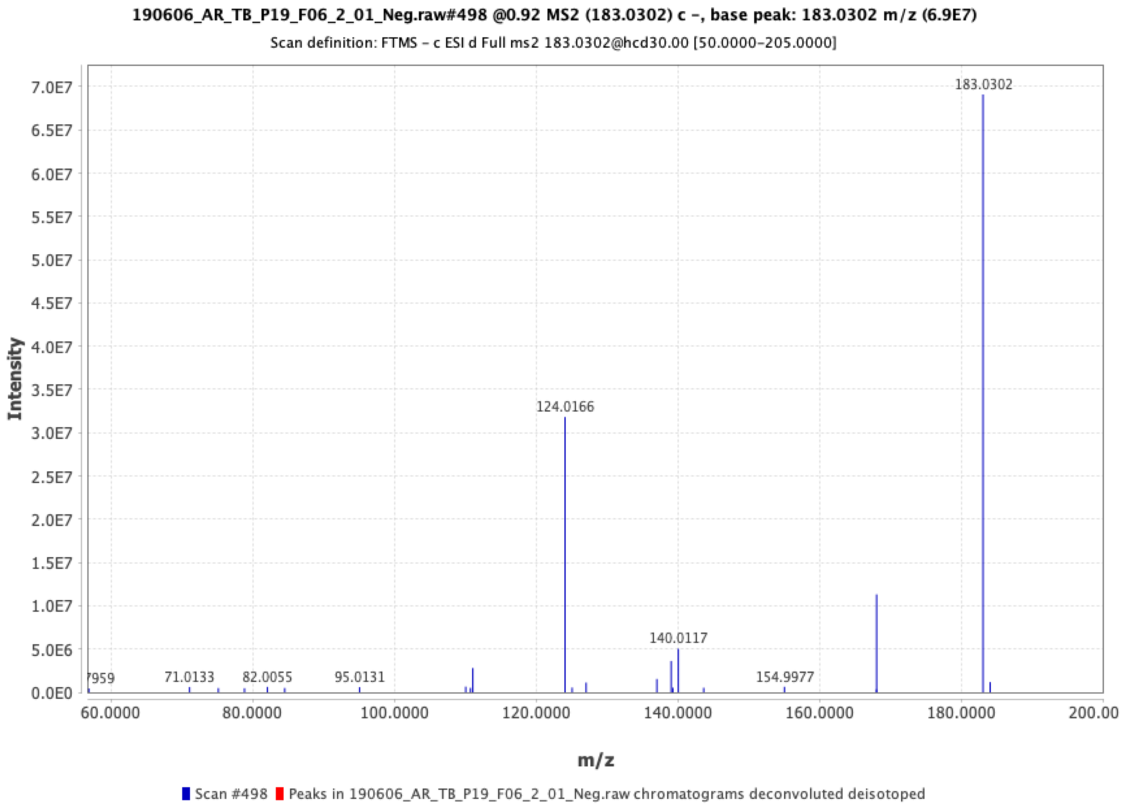

MS/MS spectra of compound **2**.

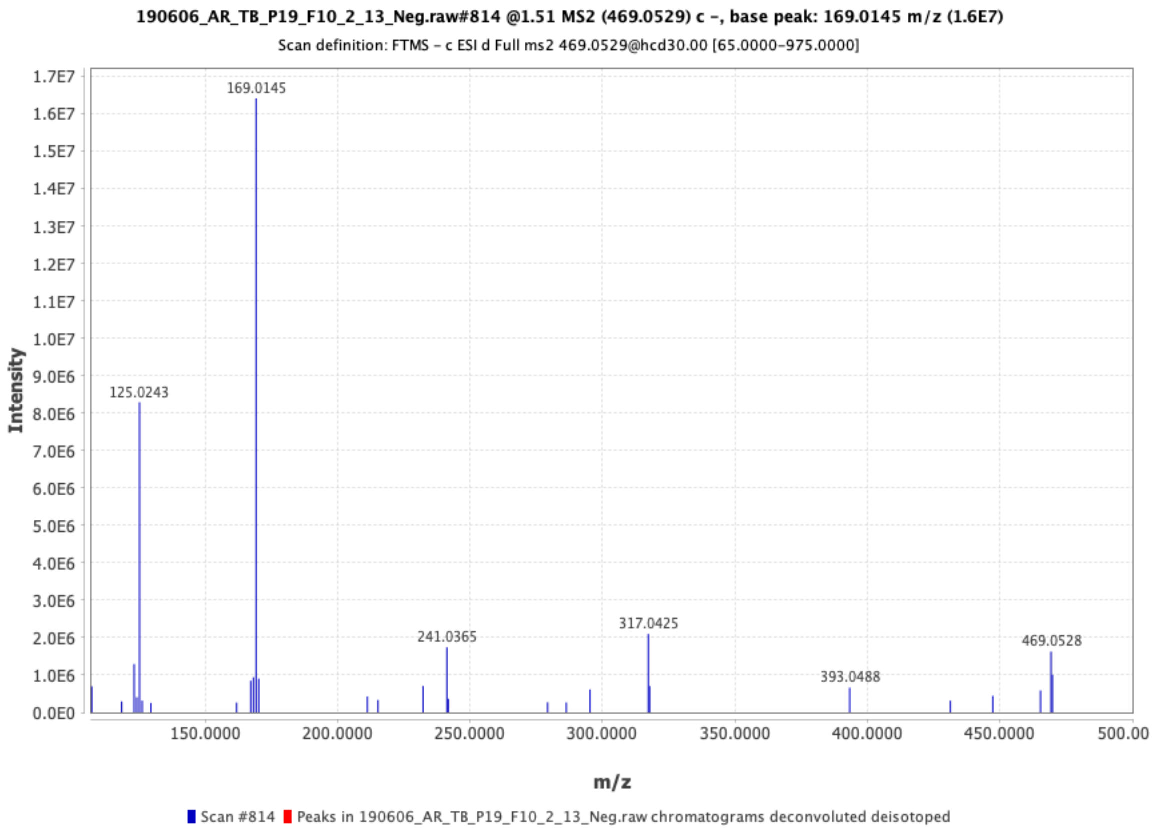

MS/MS spectra of compound **5**.

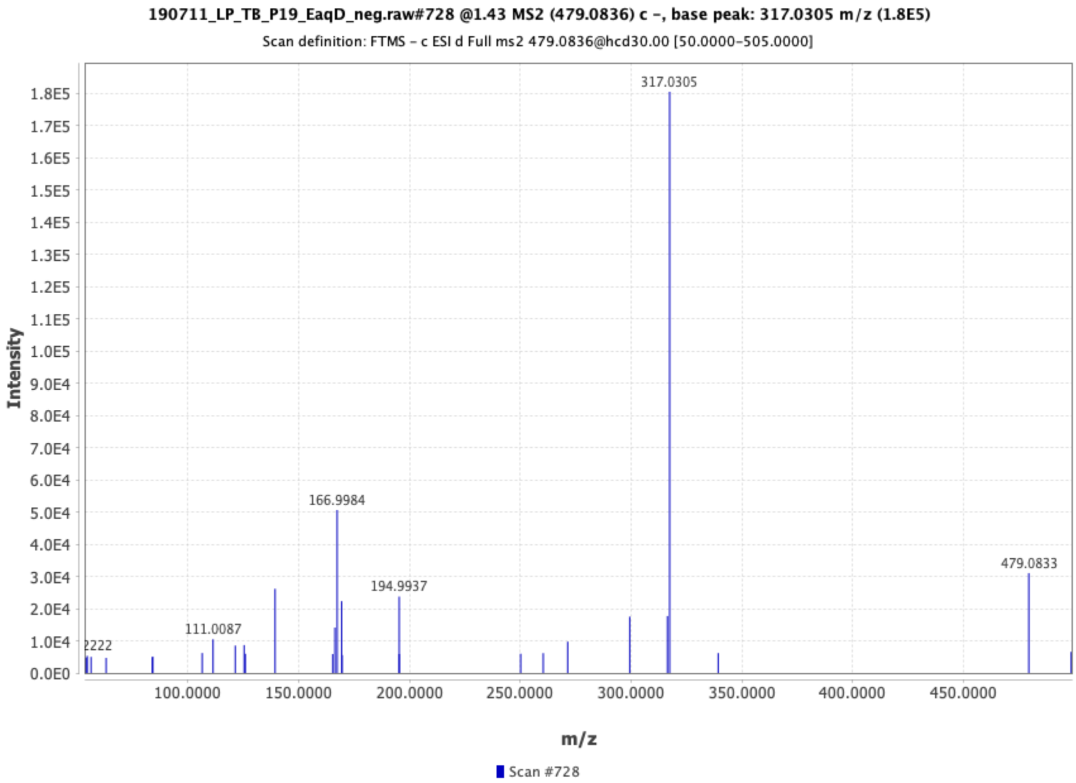

MS/MS spectra of compound **6**.

References :

Barrow, R.A., Capon, R.J., Alkyl and alkenyl resorcinols from an Australian marine sponge, *Haliclona* sp. (Haplosclerida: haliclonidae). *Aust. J. Chem.* **1991** *44*, 1393-1405.

Cui, C.B., et al., Two new and four known polyphenolics obtained as new cell-cycle inhibitors from *Rubus aleaefolius* Poir. *J Asian Nat Prod Res* **2002** *4*, 243-252.

Kamatham, S., et al., Isolation and characterization of gallic acid and methyl gallate from the seed coats of *Givotia rottleriformis* Griff. and their anti-proliferative effect on human epidermoid carcinoma A431 cells. *Toxicol Rep* **2015** *2*, 520-529.

Leela, V.; Saraswathy, A. Isolation and characterization of phytoconstituents from *Acacia leucophloea flowers* (Roxb) wild. *Int Res J Pharm* **2013**, 4, 107-109.

Tanaka, T., et al., Tannins and Related-Compounds .14. 7-*O*-Galloyl-(+)-Catechin and 3-*O*-Galloylprocyanidin-B-3 from *Sanguisorba officinalis*. *Phytochemistry* **1983** *22*, 2575-2578.

Yang, C., et al., Chemical constituents of *Pyrrosia petiolosa*. *J Asian Nat Prod Res* **2003** *5*, 143-150.

Zapesochnaya, G.; Pangarova, T. Hypolaetin 7-glucoside from *Caryopteris monolica*. *Chemistry of Natural Compounds* **1973**, 9, 521-521.
